# Supplementary material for: Updating genome annotation for the microbial cell factory Aspergillus niger using gene co-expression networks
Source: Nucleic Acids Res. 2018 Nov 29;47(2):559–69. doi: 10.1093/nar/gky1183 (PMC6344863; doi:10.1093/nar/gky1183)
Supplement: Supplementary Data [file gky1183_supplemental_files.zip › Suppl. File 1_sub-network size distribution.pptx]

## Slide 1
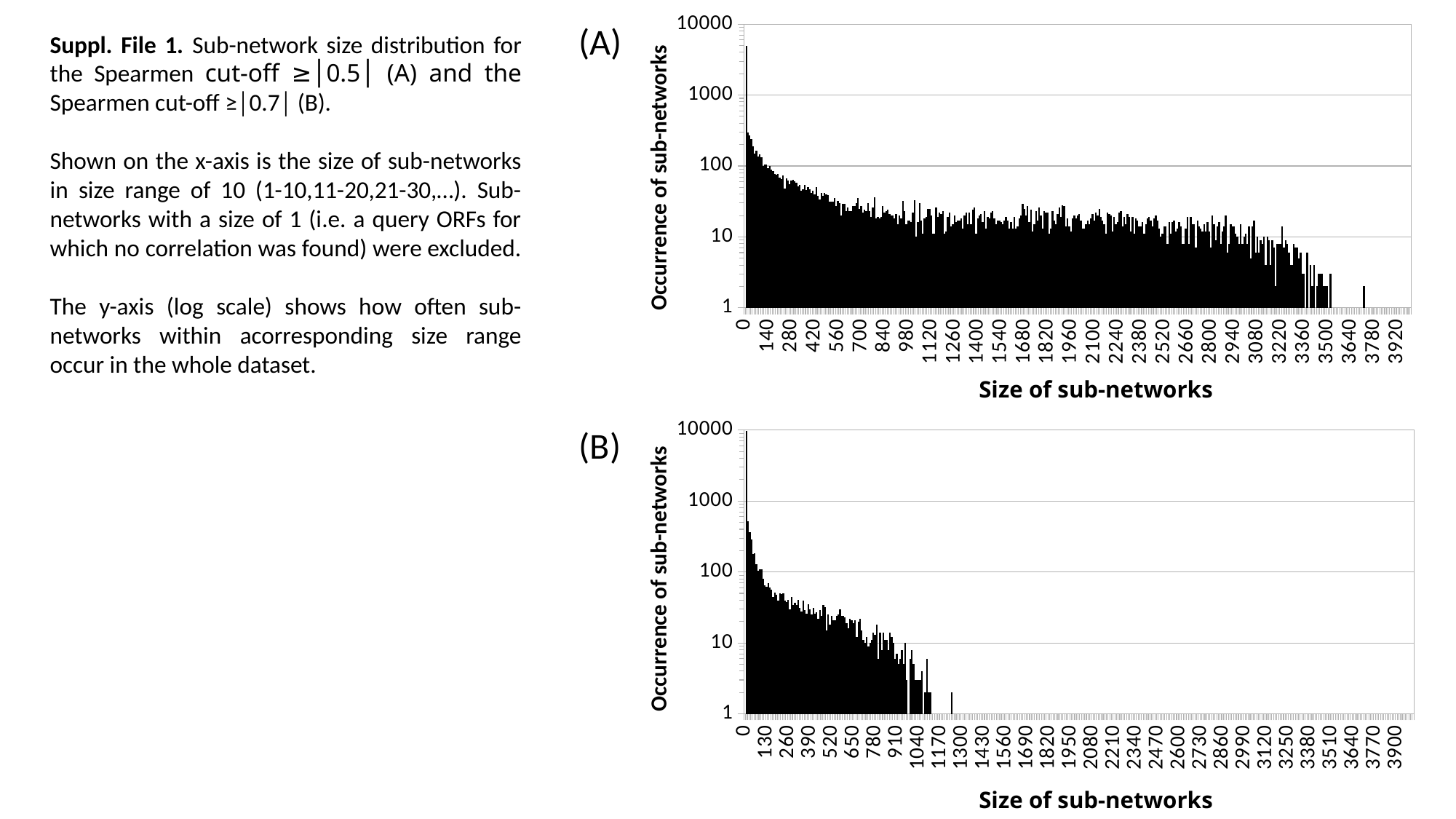

### Chart
| Category | |
|---|---|
| 0 | None |
| 10 | 4934.0 |
| 20 | 296.0 |
| 30 | 270.0 |
| 40 | 237.0 |
| 50 | 190.0 |
| 60 | 151.0 |
| 70 | 163.0 |
| 80 | 136.0 |
| 90 | 147.0 |
| 100 | 134.0 |
| 110 | 99.0 |
| 120 | 105.0 |
| 130 | 106.0 |
| 140 | 93.0 |
| 150 | 101.0 |
| 160 | 88.0 |
| 170 | 84.0 |
| 180 | 77.0 |
| 190 | 75.0 |
| 200 | 77.0 |
| 210 | 69.0 |
| 220 | 65.0 |
| 230 | 73.0 |
| 240 | 48.0 |
| 250 | 67.0 |
| 260 | 62.0 |
| 270 | 55.0 |
| 280 | 63.0 |
| 290 | 64.0 |
| 300 | 61.0 |
| 310 | 58.0 |
| 320 | 52.0 |
| 330 | 54.0 |
| 340 | 45.0 |
| 350 | 47.0 |
| 360 | 54.0 |
| 370 | 46.0 |
| 380 | 50.0 |
| 390 | 47.0 |
| 400 | 42.0 |
| 410 | 45.0 |
| 420 | 40.0 |
| 430 | 50.0 |
| 440 | 38.0 |
| 450 | 34.0 |
| 460 | 42.0 |
| 470 | 38.0 |
| 480 | 42.0 |
| 490 | 40.0 |
| 500 | 39.0 |
| 510 | 31.0 |
| 520 | 31.0 |
| 530 | 31.0 |
| 540 | 35.0 |
| 550 | 27.0 |
| 560 | 32.0 |
| 570 | 30.0 |
| 580 | 20.0 |
| 590 | 29.0 |
| 600 | 29.0 |
| 610 | 23.0 |
| 620 | 26.0 |
| 630 | 23.0 |
| 640 | 23.0 |
| 650 | 27.0 |
| 660 | 27.0 |
| 670 | 30.0 |
| 680 | 35.0 |
| 690 | 25.0 |
| 700 | 27.0 |
| 710 | 22.0 |
| 720 | 24.0 |
| 730 | 23.0 |
| 740 | 30.0 |
| 750 | 23.0 |
| 760 | 19.0 |
| 770 | 26.0 |
| 780 | 36.0 |
| 790 | 18.0 |
| 800 | 19.0 |
| 810 | 18.0 |
| 820 | 19.0 |
| 830 | 27.0 |
| 840 | 22.0 |
| 850 | 23.0 |
| 860 | 24.0 |
| 870 | 21.0 |
| 880 | 20.0 |
| 890 | 20.0 |
| 900 | 18.0 |
| 910 | 21.0 |
| 920 | 15.0 |
| 930 | 20.0 |
| 940 | 18.0 |
| 950 | 32.0 |
| 960 | 23.0 |
| 970 | 15.0 |
| 980 | 17.0 |
| 990 | 17.0 |
| 1000 | 16.0 |
| 1010 | 22.0 |
| 1020 | 33.0 |
| 1030 | 10.0 |
| 1040 | 16.0 |
| 1050 | 30.0 |
| 1060 | 17.0 |
| 1070 | 11.0 |
| 1080 | 18.0 |
| 1090 | 19.0 |
| 1100 | 25.0 |
| 1110 | 25.0 |
| 1120 | 20.0 |
| 1130 | 11.0 |
| 1140 | 11.0 |
| 1150 | 26.0 |
| 1160 | 19.0 |
| 1170 | 22.0 |
| 1180 | 21.0 |
| 1190 | 23.0 |
| 1200 | 11.0 |
| 1210 | 12.0 |
| 1220 | 19.0 |
| 1230 | 22.0 |
| 1240 | 14.0 |
| 1250 | 15.0 |
| 1260 | 20.0 |
| 1270 | 16.0 |
| 1280 | 17.0 |
| 1290 | 17.0 |
| 1300 | 18.0 |
| 1310 | 13.0 |
| 1320 | 20.0 |
| 1330 | 22.0 |
| 1340 | 15.0 |
| 1350 | 22.0 |
| 1360 | 15.0 |
| 1370 | 24.0 |
| 1380 | 26.0 |
| 1390 | 11.0 |
| 1400 | 18.0 |
| 1410 | 20.0 |
| 1420 | 21.0 |
| 1430 | 16.0 |
| 1440 | 23.0 |
| 1450 | 13.0 |
| 1460 | 19.0 |
| 1470 | 18.0 |
| 1480 | 22.0 |
| 1490 | 23.0 |
| 1500 | 18.0 |
| 1510 | 15.0 |
| 1520 | 17.0 |
| 1530 | 17.0 |
| 1540 | 16.0 |
| 1550 | 15.0 |
| 1560 | 17.0 |
| 1570 | 19.0 |
| 1580 | 17.0 |
| 1590 | 13.0 |
| 1600 | 16.0 |
| 1610 | 13.0 |
| 1620 | 19.0 |
| 1630 | 13.0 |
| 1640 | 14.0 |
| 1650 | 18.0 |
| 1660 | 20.0 |
| 1670 | 29.0 |
| 1680 | 25.0 |
| 1690 | 20.0 |
| 1700 | 27.0 |
| 1710 | 16.0 |
| 1720 | 24.0 |
| 1730 | 12.0 |
| 1740 | 15.0 |
| 1750 | 23.0 |
| 1760 | 17.0 |
| 1770 | 26.0 |
| 1780 | 20.0 |
| 1790 | 13.0 |
| 1800 | 23.0 |
| 1810 | 22.0 |
| 1820 | 22.0 |
| 1830 | 11.0 |
| 1840 | 13.0 |
| 1850 | 23.0 |
| 1860 | 17.0 |
| 1870 | 15.0 |
| 1880 | 21.0 |
| 1890 | 26.0 |
| 1900 | 19.0 |
| 1910 | 28.0 |
| 1920 | 27.0 |
| 1930 | 14.0 |
| 1940 | 18.0 |
| 1950 | 14.0 |
| 1960 | 12.0 |
| 1970 | 18.0 |
| 1980 | 20.0 |
| 1990 | 18.0 |
| 2000 | 20.0 |
| 2010 | 21.0 |
| 2020 | 17.0 |
| 2030 | 13.0 |
| 2040 | 13.0 |
| 2050 | 15.0 |
| 2060 | 17.0 |
| 2070 | 15.0 |
| 2080 | 18.0 |
| 2090 | 21.0 |
| 2100 | 17.0 |
| 2110 | 22.0 |
| 2120 | 20.0 |
| 2130 | 25.0 |
| 2140 | 19.0 |
| 2150 | 17.0 |
| 2160 | 15.0 |
| 2170 | 11.0 |
| 2180 | 22.0 |
| 2190 | 21.0 |
| 2200 | 20.0 |
| 2210 | 12.0 |
| 2220 | 19.0 |
| 2230 | 15.0 |
| 2240 | 16.0 |
| 2250 | 22.0 |
| 2260 | 23.0 |
| 2270 | 14.0 |
| 2280 | 19.0 |
| 2290 | 15.0 |
| 2300 | 21.0 |
| 2310 | 19.0 |
| 2320 | 12.0 |
| 2330 | 19.0 |
| 2340 | 11.0 |
| 2350 | 18.0 |
| 2360 | 17.0 |
| 2370 | 14.0 |
| 2380 | 14.0 |
| 2390 | 16.0 |
| 2400 | 11.0 |
| 2410 | 15.0 |
| 2420 | 18.0 |
| 2430 | 19.0 |
| 2440 | 17.0 |
| 2450 | 14.0 |
| 2460 | 18.0 |
| 2470 | 20.0 |
| 2480 | 17.0 |
| 2490 | 13.0 |
| 2500 | 10.0 |
| 2510 | 11.0 |
| 2520 | 14.0 |
| 2530 | 14.0 |
| 2540 | 8.0 |
| 2550 | 16.0 |
| 2560 | 11.0 |
| 2570 | 16.0 |
| 2580 | 17.0 |
| 2590 | 12.0 |
| 2600 | 13.0 |
| 2610 | 16.0 |
| 2620 | 14.0 |
| 2630 | 8.0 |
| 2640 | 8.0 |
| 2650 | 13.0 |
| 2660 | 19.0 |
| 2670 | 8.0 |
| 2680 | 19.0 |
| 2690 | 15.0 |
| 2700 | 15.0 |
| 2710 | 7.0 |
| 2720 | 17.0 |
| 2730 | 14.0 |
| 2740 | 13.0 |
| 2750 | 12.0 |
| 2760 | 15.0 |
| 2770 | 12.0 |
| 2780 | 16.0 |
| 2790 | 12.0 |
| 2800 | 7.0 |
| 2810 | 20.0 |
| 2820 | 15.0 |
| 2830 | 9.0 |
| 2840 | 14.0 |
| 2850 | 16.0 |
| 2860 | 8.0 |
| 2870 | 12.0 |
| 2880 | 14.0 |
| 2890 | 20.0 |
| 2900 | 6.0 |
| 2910 | 8.0 |
| 2920 | 15.0 |
| 2930 | 14.0 |
| 2940 | 14.0 |
| 2950 | 11.0 |
| 2960 | 10.0 |
| 2970 | 8.0 |
| 2980 | 15.0 |
| 2990 | 8.0 |
| 3000 | 10.0 |
| 3010 | 11.0 |
| 3020 | 8.0 |
| 3030 | 14.0 |
| 3040 | 5.0 |
| 3050 | 14.0 |
| 3060 | 17.0 |
| 3070 | 6.0 |
| 3080 | 10.0 |
| 3090 | 6.0 |
| 3100 | 9.0 |
| 3110 | 8.0 |
| 3120 | 10.0 |
| 3130 | 4.0 |
| 3140 | 10.0 |
| 3150 | 9.0 |
| 3160 | 4.0 |
| 3170 | 9.0 |
| 3180 | 7.0 |
| 3190 | 2.0 |
| 3200 | 8.0 |
| 3210 | 8.0 |
| 3220 | 8.0 |
| 3230 | 14.0 |
| 3240 | 7.0 |
| 3250 | 9.0 |
| 3260 | 8.0 |
| 3270 | 6.0 |
| 3280 | 4.0 |
| 3290 | 4.0 |
| 3300 | 8.0 |
| 3310 | 7.0 |
| 3320 | 7.0 |
| 3330 | 5.0 |
| 3340 | 6.0 |
| 3350 | 3.0 |
| 3360 | 3.0 |
| 3370 | 0.0 |
| 3380 | 6.0 |
| 3390 | 1.0 |
| 3400 | 4.0 |
| 3410 | 2.0 |
| 3420 | 4.0 |
| 3430 | 1.0 |
| 3440 | 2.0 |
| 3450 | 3.0 |
| 3460 | 3.0 |
| 3470 | 3.0 |
| 3480 | 2.0 |
| 3490 | 2.0 |
| 3500 | 2.0 |
| 3510 | 0.0 |
| 3520 | 3.0 |
| 3530 | 0.0 |
| 3540 | 0.0 |
| 3550 | 0.0 |
| 3560 | 1.0 |
| 3570 | 1.0 |
| 3580 | 0.0 |
| 3590 | 0.0 |
| 3600 | 0.0 |
| 3610 | 0.0 |
| 3620 | 0.0 |
| 3630 | 1.0 |
| 3640 | 0.0 |
| 3650 | 0.0 |
| 3660 | 0.0 |
| 3670 | 0.0 |
| 3680 | 1.0 |
| 3690 | 0.0 |
| 3700 | 0.0 |
| 3710 | 0.0 |
| 3720 | 2.0 |
| 3730 | 0.0 |
| 3740 | 0.0 |
| 3750 | 0.0 |
| 3760 | 0.0 |
| 3770 | 0.0 |
| 3780 | 0.0 |
| 3790 | 1.0 |
| 3800 | 0.0 |
| 3810 | 0.0 |
| 3820 | 0.0 |
| 3830 | 0.0 |
| 3840 | 0.0 |
| 3850 | 0.0 |
| 3860 | 0.0 |
| 3870 | 0.0 |
| 3880 | 0.0 |
| 3890 | 0.0 |
| 3900 | 0.0 |
| 3910 | 0.0 |
| 3920 | 0.0 |
| 3930 | 0.0 |
| 3940 | 0.0 |
| 3950 | 0.0 |
| 3960 | 0.0 |
| 3970 | 0.0 |
| 3980 | 0.0 |
| 3990 | 0.0 |
| 4000 | 0.0 |(A)
Suppl. File 1. Sub-network size distribution for the Spearmen cut-off ≥│0.5│ (A) and the Spearmen cut-off ≥│0.7│ (B).
Shown on the x-axis is the size of sub-networks in size range of 10 (1-10,11-20,21-30,…). Sub-networks with a size of 1 (i.e. a query ORFs for which no correlation was found) were excluded.
The y-axis (log scale) shows how often sub-networks within acorresponding size range occur in the whole dataset.
Occurrence of sub-networks
Size of sub-networks
### Chart
| Category | |
|---|---|
| 0 | None |
| 10 | 9677.0 |
| 20 | 522.0 |
| 30 | 362.0 |
| 40 | 290.0 |
| 50 | 181.0 |
| 60 | 182.0 |
| 70 | 128.0 |
| 80 | 105.0 |
| 90 | 108.0 |
| 100 | 110.0 |
| 110 | 80.0 |
| 120 | 65.0 |
| 130 | 62.0 |
| 140 | 69.0 |
| 150 | 61.0 |
| 160 | 56.0 |
| 170 | 44.0 |
| 180 | 51.0 |
| 190 | 48.0 |
| 200 | 39.0 |
| 210 | 50.0 |
| 220 | 49.0 |
| 230 | 50.0 |
| 240 | 39.0 |
| 250 | 38.0 |
| 260 | 40.0 |
| 270 | 30.0 |
| 280 | 44.0 |
| 290 | 34.0 |
| 300 | 37.0 |
| 310 | 34.0 |
| 320 | 40.0 |
| 330 | 31.0 |
| 340 | 28.0 |
| 350 | 39.0 |
| 360 | 29.0 |
| 370 | 26.0 |
| 380 | 35.0 |
| 390 | 30.0 |
| 400 | 25.0 |
| 410 | 31.0 |
| 420 | 26.0 |
| 430 | 27.0 |
| 440 | 22.0 |
| 450 | 29.0 |
| 460 | 24.0 |
| 470 | 34.0 |
| 480 | 32.0 |
| 490 | 15.0 |
| 500 | 25.0 |
| 510 | 18.0 |
| 520 | 24.0 |
| 530 | 21.0 |
| 540 | 21.0 |
| 550 | 24.0 |
| 560 | 25.0 |
| 570 | 30.0 |
| 580 | 24.0 |
| 590 | 24.0 |
| 600 | 23.0 |
| 610 | 19.0 |
| 620 | 16.0 |
| 630 | 22.0 |
| 640 | 21.0 |
| 650 | 19.0 |
| 660 | 21.0 |
| 670 | 12.0 |
| 680 | 20.0 |
| 690 | 22.0 |
| 700 | 15.0 |
| 710 | 11.0 |
| 720 | 10.0 |
| 730 | 12.0 |
| 740 | 9.0 |
| 750 | 10.0 |
| 760 | 11.0 |
| 770 | 14.0 |
| 780 | 13.0 |
| 790 | 18.0 |
| 800 | 6.0 |
| 810 | 14.0 |
| 820 | 8.0 |
| 830 | 14.0 |
| 840 | 11.0 |
| 850 | 11.0 |
| 860 | 8.0 |
| 870 | 14.0 |
| 880 | 12.0 |
| 890 | 10.0 |
| 900 | 6.0 |
| 910 | 7.0 |
| 920 | 5.0 |
| 930 | 6.0 |
| 940 | 8.0 |
| 950 | 5.0 |
| 960 | 10.0 |
| 970 | 3.0 |
| 980 | 1.0 |
| 990 | 6.0 |
| 1000 | 8.0 |
| 1010 | 5.0 |
| 1020 | 3.0 |
| 1030 | 3.0 |
| 1040 | 3.0 |
| 1050 | 3.0 |
| 1060 | 4.0 |
| 1070 | 1.0 |
| 1080 | 2.0 |
| 1090 | 6.0 |
| 1100 | 2.0 |
| 1110 | 2.0 |
| 1120 | 1.0 |
| 1130 | 1.0 |
| 1140 | 0.0 |
| 1150 | 1.0 |
| 1160 | 0.0 |
| 1170 | 0.0 |
| 1180 | 0.0 |
| 1190 | 0.0 |
| 1200 | 0.0 |
| 1210 | 1.0 |
| 1220 | 0.0 |
| 1230 | 0.0 |
| 1240 | 2.0 |
| 1250 | 0.0 |
| 1260 | 0.0 |
| 1270 | 0.0 |
| 1280 | 0.0 |
| 1290 | 0.0 |
| 1300 | 0.0 |
| 1310 | 0.0 |
| 1320 | 0.0 |
| 1330 | 0.0 |
| 1340 | 0.0 |
| 1350 | 0.0 |
| 1360 | 0.0 |
| 1370 | 0.0 |
| 1380 | 0.0 |
| 1390 | 0.0 |
| 1400 | 0.0 |
| 1410 | 0.0 |
| 1420 | 0.0 |
| 1430 | 0.0 |
| 1440 | 0.0 |
| 1450 | 0.0 |
| 1460 | 0.0 |
| 1470 | 0.0 |
| 1480 | 0.0 |
| 1490 | 0.0 |
| 1500 | 0.0 |
| 1510 | 0.0 |
| 1520 | 0.0 |
| 1530 | 0.0 |
| 1540 | 0.0 |
| 1550 | 0.0 |
| 1560 | 0.0 |
| 1570 | 0.0 |
| 1580 | 0.0 |
| 1590 | 0.0 |
| 1600 | 0.0 |
| 1610 | 0.0 |
| 1620 | 0.0 |
| 1630 | 0.0 |
| 1640 | 0.0 |
| 1650 | 0.0 |
| 1660 | 0.0 |
| 1670 | 0.0 |
| 1680 | 0.0 |
| 1690 | 0.0 |
| 1700 | 0.0 |
| 1710 | 0.0 |
| 1720 | 0.0 |
| 1730 | 0.0 |
| 1740 | 0.0 |
| 1750 | 0.0 |
| 1760 | 0.0 |
| 1770 | 0.0 |
| 1780 | 0.0 |
| 1790 | 0.0 |
| 1800 | 0.0 |
| 1810 | 0.0 |
| 1820 | 0.0 |
| 1830 | 0.0 |
| 1840 | 0.0 |
| 1850 | 0.0 |
| 1860 | 0.0 |
| 1870 | 0.0 |
| 1880 | 0.0 |
| 1890 | 0.0 |
| 1900 | 0.0 |
| 1910 | 0.0 |
| 1920 | 0.0 |
| 1930 | 0.0 |
| 1940 | 0.0 |
| 1950 | 0.0 |
| 1960 | 0.0 |
| 1970 | 0.0 |
| 1980 | 0.0 |
| 1990 | 0.0 |
| 2000 | 0.0 |
| 2010 | 0.0 |
| 2020 | 0.0 |
| 2030 | 0.0 |
| 2040 | 0.0 |
| 2050 | 0.0 |
| 2060 | 0.0 |
| 2070 | 0.0 |
| 2080 | 0.0 |
| 2090 | 0.0 |
| 2100 | 0.0 |
| 2110 | 0.0 |
| 2120 | 0.0 |
| 2130 | 0.0 |
| 2140 | 0.0 |
| 2150 | 0.0 |
| 2160 | 0.0 |
| 2170 | 0.0 |
| 2180 | 0.0 |
| 2190 | 0.0 |
| 2200 | 0.0 |
| 2210 | 0.0 |
| 2220 | 0.0 |
| 2230 | 0.0 |
| 2240 | 0.0 |
| 2250 | 0.0 |
| 2260 | 0.0 |
| 2270 | 0.0 |
| 2280 | 0.0 |
| 2290 | 0.0 |
| 2300 | 0.0 |
| 2310 | 0.0 |
| 2320 | 0.0 |
| 2330 | 0.0 |
| 2340 | 0.0 |
| 2350 | 0.0 |
| 2360 | 0.0 |
| 2370 | 0.0 |
| 2380 | 0.0 |
| 2390 | 0.0 |
| 2400 | 0.0 |
| 2410 | 0.0 |
| 2420 | 0.0 |
| 2430 | 0.0 |
| 2440 | 0.0 |
| 2450 | 0.0 |
| 2460 | 0.0 |
| 2470 | 0.0 |
| 2480 | 0.0 |
| 2490 | 0.0 |
| 2500 | 0.0 |
| 2510 | 0.0 |
| 2520 | 0.0 |
| 2530 | 0.0 |
| 2540 | 0.0 |
| 2550 | 0.0 |
| 2560 | 0.0 |
| 2570 | 0.0 |
| 2580 | 0.0 |
| 2590 | 0.0 |
| 2600 | 0.0 |
| 2610 | 0.0 |
| 2620 | 0.0 |
| 2630 | 0.0 |
| 2640 | 0.0 |
| 2650 | 0.0 |
| 2660 | 0.0 |
| 2670 | 0.0 |
| 2680 | 0.0 |
| 2690 | 0.0 |
| 2700 | 0.0 |
| 2710 | 0.0 |
| 2720 | 0.0 |
| 2730 | 0.0 |
| 2740 | 0.0 |
| 2750 | 0.0 |
| 2760 | 0.0 |
| 2770 | 0.0 |
| 2780 | 0.0 |
| 2790 | 0.0 |
| 2800 | 0.0 |
| 2810 | 0.0 |
| 2820 | 0.0 |
| 2830 | 0.0 |
| 2840 | 0.0 |
| 2850 | 0.0 |
| 2860 | 0.0 |
| 2870 | 0.0 |
| 2880 | 0.0 |
| 2890 | 0.0 |
| 2900 | 0.0 |
| 2910 | 0.0 |
| 2920 | 0.0 |
| 2930 | 0.0 |
| 2940 | 0.0 |
| 2950 | 0.0 |
| 2960 | 0.0 |
| 2970 | 0.0 |
| 2980 | 0.0 |
| 2990 | 0.0 |
| 3000 | 0.0 |
| 3010 | 0.0 |
| 3020 | 0.0 |
| 3030 | 0.0 |
| 3040 | 0.0 |
| 3050 | 0.0 |
| 3060 | 0.0 |
| 3070 | 0.0 |
| 3080 | 0.0 |
| 3090 | 0.0 |
| 3100 | 0.0 |
| 3110 | 0.0 |
| 3120 | 0.0 |
| 3130 | 0.0 |
| 3140 | 0.0 |
| 3150 | 0.0 |
| 3160 | 0.0 |
| 3170 | 0.0 |
| 3180 | 0.0 |
| 3190 | 0.0 |
| 3200 | 0.0 |
| 3210 | 0.0 |
| 3220 | 0.0 |
| 3230 | 0.0 |
| 3240 | 0.0 |
| 3250 | 0.0 |
| 3260 | 0.0 |
| 3270 | 0.0 |
| 3280 | 0.0 |
| 3290 | 0.0 |
| 3300 | 0.0 |
| 3310 | 0.0 |
| 3320 | 0.0 |
| 3330 | 0.0 |
| 3340 | 0.0 |
| 3350 | 0.0 |
| 3360 | 0.0 |
| 3370 | 0.0 |
| 3380 | 0.0 |
| 3390 | 0.0 |
| 3400 | 0.0 |
| 3410 | 0.0 |
| 3420 | 0.0 |
| 3430 | 0.0 |
| 3440 | 0.0 |
| 3450 | 0.0 |
| 3460 | 0.0 |
| 3470 | 0.0 |
| 3480 | 0.0 |
| 3490 | 0.0 |
| 3500 | 0.0 |
| 3510 | 0.0 |
| 3520 | 0.0 |
| 3530 | 0.0 |
| 3540 | 0.0 |
| 3550 | 0.0 |
| 3560 | 0.0 |
| 3570 | 0.0 |
| 3580 | 0.0 |
| 3590 | 0.0 |
| 3600 | 0.0 |
| 3610 | 0.0 |
| 3620 | 0.0 |
| 3630 | 0.0 |
| 3640 | 0.0 |
| 3650 | 0.0 |
| 3660 | 0.0 |
| 3670 | 0.0 |
| 3680 | 0.0 |
| 3690 | 0.0 |
| 3700 | 0.0 |
| 3710 | 0.0 |
| 3720 | 0.0 |
| 3730 | 0.0 |
| 3740 | 0.0 |
| 3750 | 0.0 |
| 3760 | 0.0 |
| 3770 | 0.0 |
| 3780 | 0.0 |
| 3790 | 0.0 |
| 3800 | 0.0 |
| 3810 | 0.0 |
| 3820 | 0.0 |
| 3830 | 0.0 |
| 3840 | 0.0 |
| 3850 | 0.0 |
| 3860 | 0.0 |
| 3870 | 0.0 |
| 3880 | 0.0 |
| 3890 | 0.0 |
| 3900 | 0.0 |
| 3910 | 0.0 |
| 3920 | 0.0 |
| 3930 | 0.0 |
| 3940 | 0.0 |
| 3950 | 0.0 |
| 3960 | 0.0 |
| 3970 | 0.0 |
| 3980 | 0.0 |
| 3990 | 0.0 |
| 4000 | 0.0 |(B)
Occurrence of sub-networks
Size of sub-networks
