# Supplementary material for: Updating genome annotation for the microbial cell factory Aspergillus niger using gene co-expression networks
Source: Nucleic Acids Res. 2018 Nov 29;47(2):559–69. doi: 10.1093/nar/gky1183 (PMC6344863; doi:10.1093/nar/gky1183)
Supplement: Supplementary Data [file gky1183_supplemental_files.zip › Suppl. File 2_heatmap.pptx]

## Slide 1
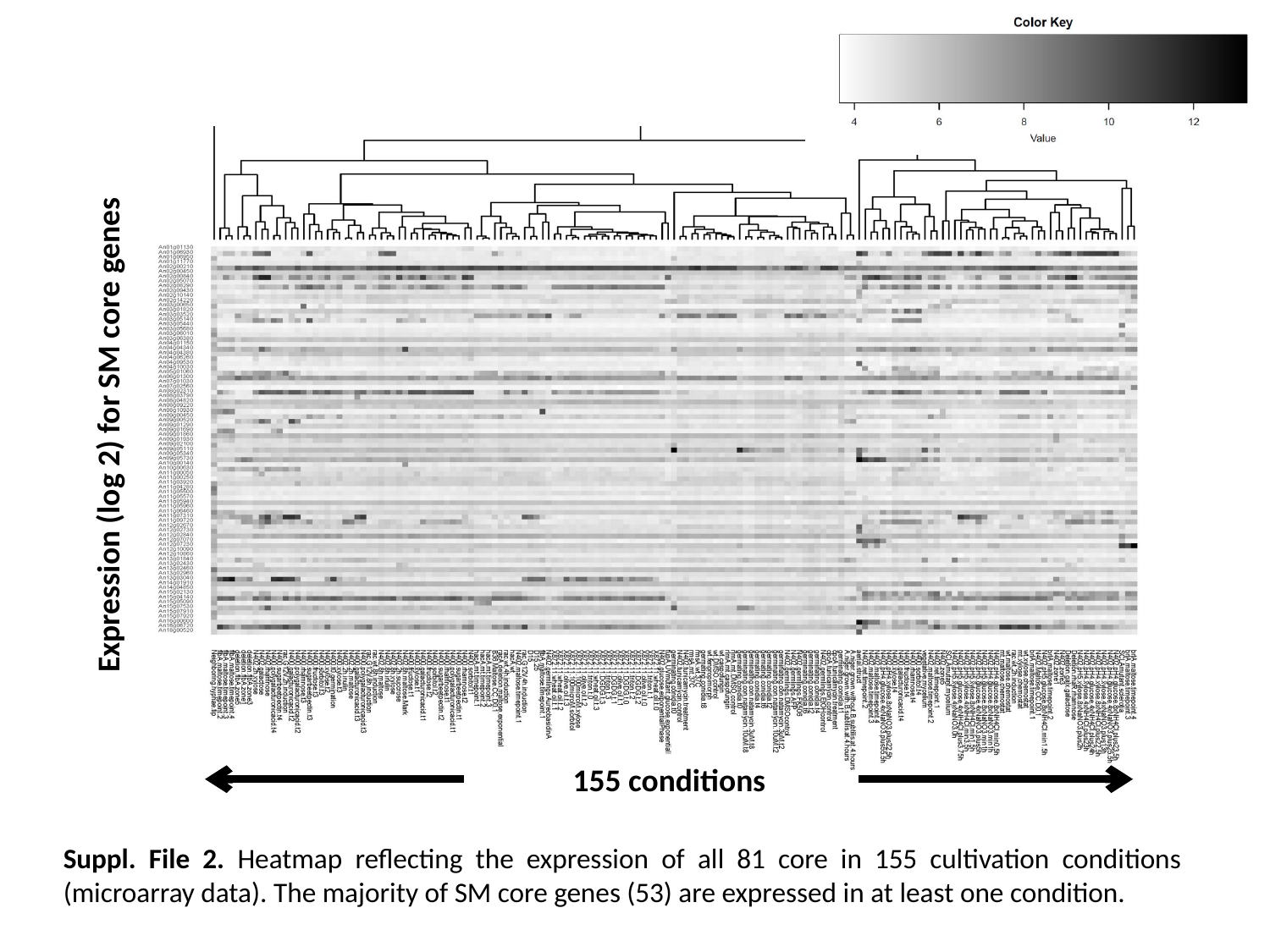

Expression (log 2) for SM core genes
155 conditions
Suppl. File 2. Heatmap reflecting the expression of all 81 core in 155 cultivation conditions (microarray data). The majority of SM core genes (53) are expressed in at least one condition.
