# Supplementary material for: Updating genome annotation for the microbial cell factory Aspergillus niger using gene co-expression networks
Source: Nucleic Acids Res. 2018 Nov 29;47(2):559–69. doi: 10.1093/nar/gky1183 (PMC6344863; doi:10.1093/nar/gky1183)
Supplement: Supplementary Data [file gky1183_supplemental_files.zip › Suppl. File 3_SM cluster coexpression.pptx]

## Slide 1
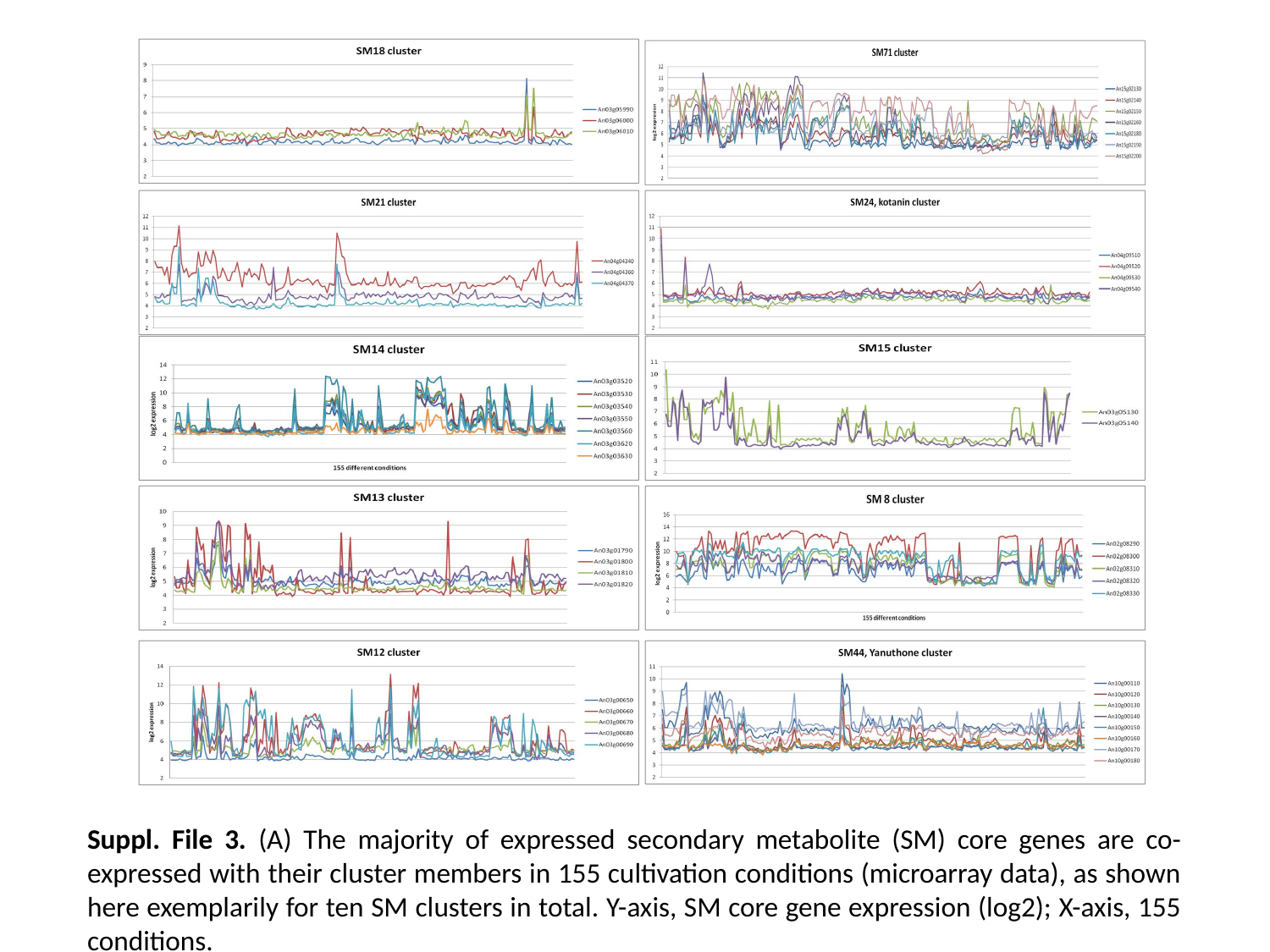

Suppl. File 3. (A) The majority of expressed secondary metabolite (SM) core genes are co-expressed with their cluster members in 155 cultivation conditions (microarray data), as shown here exemplarily for ten SM clusters in total. Y-axis, SM core gene expression (log2); X-axis, 155 conditions.

## Slide 2
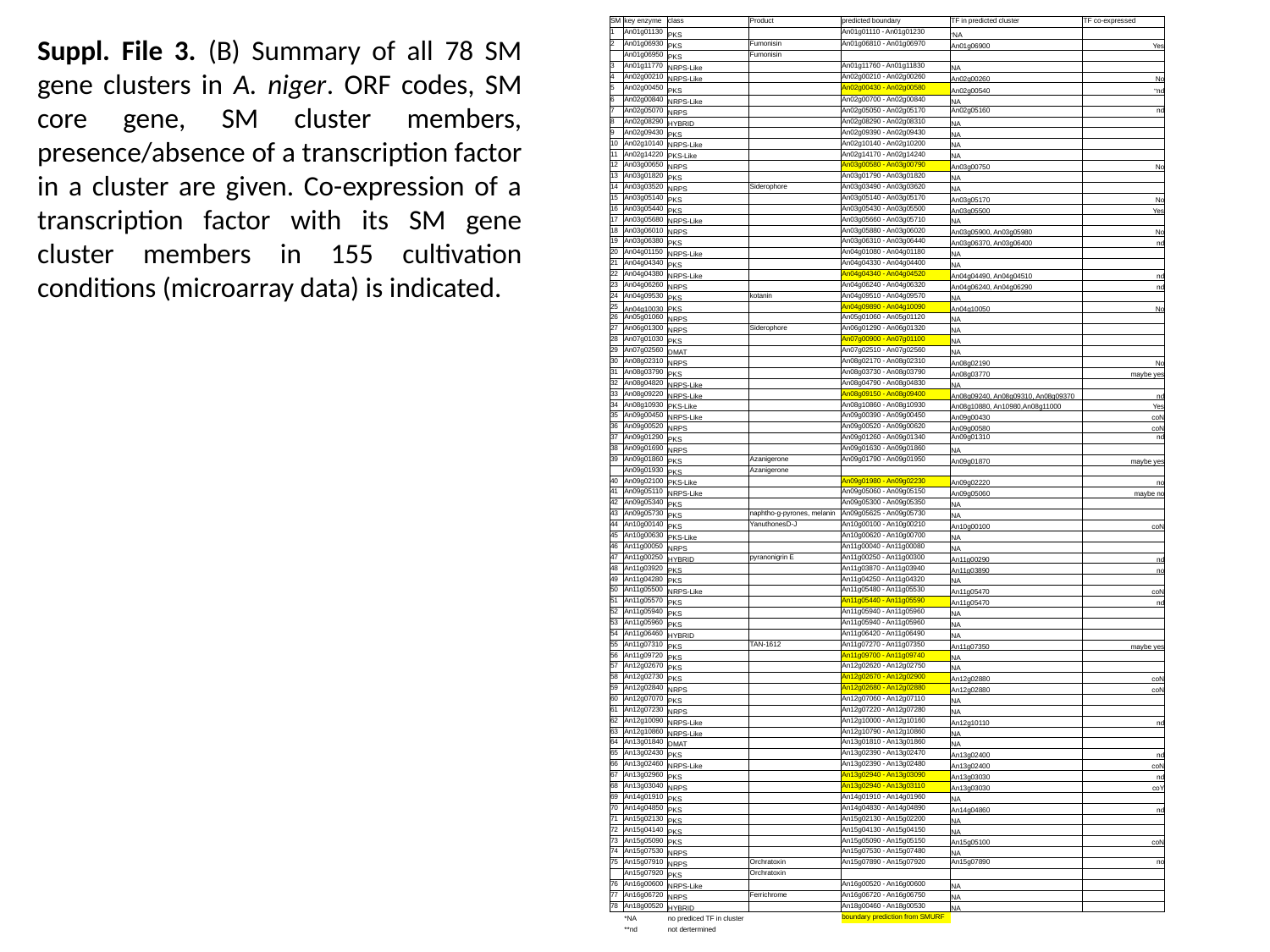

| SM | key enzyme | class | Product | predicted boundary | TF in predicted cluster | TF co-expressed |
| --- | --- | --- | --- | --- | --- | --- |
| 1 | An01g01130 | PKS | | An01g01110 - An01g01230 | \*NA | |
| 2 | An01g06930 | PKS | Fumonisin | An01g06810 - An01g06970 | An01g06900 | Yes |
| | An01g06950 | PKS | Fumonisin | | | |
| 3 | An01g11770 | NRPS-Like | | An01g11760 - An01g11830 | NA | |
| 4 | An02g00210 | NRPS-Like | | An02g00210 - An02g00260 | An02g00260 | No |
| 5 | An02g00450 | PKS | | An02g00430 - An02g00580 | An02g00540 | \*\*nd |
| 6 | An02g00840 | NRPS-Like | | An02g00700 - An02g00840 | NA | |
| 7 | An02g05070 | NRPS | | An02g05050 - An02g05170 | An02g05160 | nd |
| 8 | An02g08290 | HYBRID | | An02g08290 - An02g08310 | NA | |
| 9 | An02g09430 | PKS | | An02g09390 - An02g09430 | NA | |
| 10 | An02g10140 | NRPS-Like | | An02g10140 - An02g10200 | NA | |
| 11 | An02g14220 | PKS-Like | | An02g14170 - An02g14240 | NA | |
| 12 | An03g00650 | NRPS | | An03g00580 - An03g00790 | An03g00750 | No |
| 13 | An03g01820 | PKS | | An03g01790 - An03g01820 | NA | |
| 14 | An03g03520 | NRPS | Siderophore | An03g03490 - An03g03620 | NA | |
| 15 | An03g05140 | PKS | | An03g05140 - An03g05170 | An03g05170 | No |
| 16 | An03g05440 | PKS | | An03g05430 - An03g05500 | An03g05500 | Yes |
| 17 | An03g05680 | NRPS-Like | | An03g05660 - An03g05710 | NA | |
| 18 | An03g06010 | NRPS | | An03g05880 - An03g06020 | An03g05900, An03g05980 | No |
| 19 | An03g06380 | PKS | | An03g06310 - An03g06440 | An03g06370, An03g06400 | nd |
| 20 | An04g01150 | NRPS-Like | | An04g01080 - An04g01180 | NA | |
| 21 | An04g04340 | PKS | | An04g04330 - An04g04400 | NA | |
| 22 | An04g04380 | NRPS-Like | | An04g04340 - An04g04520 | An04g04490, An04g04510 | nd |
| 23 | An04g06260 | NRPS | | An04g06240 - An04g06320 | An04g06240, An04g06290 | nd |
| 24 | An04g09530 | PKS | kotanin | An04g09510 - An04g09570 | NA | |
| 25 | An04g10030 | PKS | | An04g09890 - An04g10090 | An04g10050 | No |
| 26 | An05g01060 | NRPS | | An05g01060 - An05g01120 | NA | |
| 27 | An06g01300 | NRPS | Siderophore | An06g01290 - An06g01320 | NA | |
| 28 | An07g01030 | PKS | | An07g00900 - An07g01100 | NA | |
| 29 | An07g02560 | DMAT | | An07g02510 - An07g02560 | NA | |
| 30 | An08g02310 | NRPS | | An08g02170 - An08g02310 | An08g02190 | No |
| 31 | An08g03790 | PKS | | An08g03730 - An08g03790 | An08g03770 | maybe yes |
| 32 | An08g04820 | NRPS-Like | | An08g04790 - An08g04830 | NA | |
| 33 | An08g09220 | NRPS-Like | | An08g09150 - An08g09400 | An08g09240, An08g09310, An08g09370 | nd |
| 34 | An08g10930 | PKS-Like | | An08g10860 - An08g10930 | An08g10880, An10980,An08g11000 | Yes |
| 35 | An09g00450 | NRPS-Like | | An09g00390 - An09g00450 | An09g00430 | coN |
| 36 | An09g00520 | NRPS | | An09g00520 - An09g00620 | An09g00580 | coN |
| 37 | An09g01290 | PKS | | An09g01260 - An09g01340 | An09g01310 | nd |
| 38 | An09g01690 | NRPS | | An09g01630 - An09g01860 | NA | |
| 39 | An09g01860 | PKS | Azanigerone | An09g01790 - An09g01950 | An09g01870 | maybe yes |
| | An09g01930 | PKS | Azanigerone | | | |
| 40 | An09g02100 | PKS-Like | | An09g01980 - An09g02230 | An09g02220 | no |
| 41 | An09g05110 | NRPS-Like | | An09g05060 - An09g05150 | An09g05060 | maybe no |
| 42 | An09g05340 | PKS | | An09g05300 - An09g05350 | NA | |
| 43 | An09g05730 | PKS | naphtho-g-pyrones, melanin | An09g05625 - An09g05730 | NA | |
| 44 | An10g00140 | PKS | YanuthonesD-J | An10g00100 - An10g00210 | An10g00100 | coN |
| 45 | An10g00630 | PKS-Like | | An10g00620 - An10g00700 | NA | |
| 46 | An11g00050 | NRPS | | An11g00040 - An11g00080 | NA | |
| 47 | An11g00250 | HYBRID | pyranonigrin E | An11g00250 - An11g00300 | An11g00290 | nd |
| 48 | An11g03920 | PKS | | An11g03870 - An11g03940 | An11g03890 | no |
| 49 | An11g04280 | PKS | | An11g04250 - An11g04320 | NA | |
| 50 | An11g05500 | NRPS-Like | | An11g05480 - An11g05530 | An11g05470 | coN |
| 51 | An11g05570 | PKS | | An11g05440 - An11g05590 | An11g05470 | nd |
| 52 | An11g05940 | PKS | | An11g05940 - An11g05960 | NA | |
| 53 | An11g05960 | PKS | | An11g05940 - An11g05960 | NA | |
| 54 | An11g06460 | HYBRID | | An11g06420 - An11g06490 | NA | |
| 55 | An11g07310 | PKS | TAN-1612 | An11g07270 - An11g07350 | An11g07350 | maybe yes |
| 56 | An11g09720 | PKS | | An11g09700 - An11g09740 | NA | |
| 57 | An12g02670 | PKS | | An12g02620 - An12g02750 | NA | |
| 58 | An12g02730 | PKS | | An12g02670 - An12g02900 | An12g02880 | coN |
| 59 | An12g02840 | NRPS | | An12g02680 - An12g02880 | An12g02880 | coN |
| 60 | An12g07070 | PKS | | An12g07060 - An12g07110 | NA | |
| 61 | An12g07230 | NRPS | | An12g07220 - An12g07280 | NA | |
| 62 | An12g10090 | NRPS-Like | | An12g10000 - An12g10160 | An12g10110 | nd |
| 63 | An12g10860 | NRPS-Like | | An12g10790 - An12g10860 | NA | |
| 64 | An13g01840 | DMAT | | An13g01810 - An13g01860 | NA | |
| 65 | An13g02430 | PKS | | An13g02390 - An13g02470 | An13g02400 | nd |
| 66 | An13g02460 | NRPS-Like | | An13g02390 - An13g02480 | An13g02400 | coN |
| 67 | An13g02960 | PKS | | An13g02940 - An13g03090 | An13g03030 | nd |
| 68 | An13g03040 | NRPS | | An13g02940 - An13g03110 | An13g03030 | coY |
| 69 | An14g01910 | PKS | | An14g01910 - An14g01960 | NA | |
| 70 | An14g04850 | PKS | | An14g04830 - An14g04890 | An14g04860 | nd |
| 71 | An15g02130 | PKS | | An15g02130 - An15g02200 | NA | |
| 72 | An15g04140 | PKS | | An15g04130 - An15g04150 | NA | |
| 73 | An15g05090 | PKS | | An15g05090 - An15g05150 | An15g05100 | coN |
| 74 | An15g07530 | NRPS | | An15g07530 - An15g07480 | NA | |
| 75 | An15g07910 | NRPS | Orchratoxin | An15g07890 - An15g07920 | An15g07890 | no |
| | An15g07920 | PKS | Orchratoxin | | | |
| 76 | An16g00600 | NRPS-Like | | An16g00520 - An16g00600 | NA | |
| 77 | An16g06720 | NRPS | Ferrichrome | An16g06720 - An16g06750 | NA | |
| 78 | An18g00520 | HYBRID | | An18g00460 - An18g00530 | NA | |
| | \*NA | no prediced TF in cluster | | boundary prediction from SMURF | | |
| | \*\*nd | not dertermined | | | | |
Suppl. File 3. (B) Summary of all 78 SM gene clusters in A. niger. ORF codes, SM core gene, SM cluster members, presence/absence of a transcription factor in a cluster are given. Co-expression of a transcription factor with its SM gene cluster members in 155 cultivation conditions (microarray data) is indicated.
