# Supplementary material for: Updating genome annotation for the microbial cell factory Aspergillus niger using gene co-expression networks
Source: Nucleic Acids Res. 2018 Nov 29;47(2):559–69. doi: 10.1093/nar/gky1183 (PMC6344863; doi:10.1093/nar/gky1183)
Supplement: Supplementary Data [file gky1183_supplemental_files.zip › Suppl. File 4_metabolite and gene expression.pptx]

## Slide 1
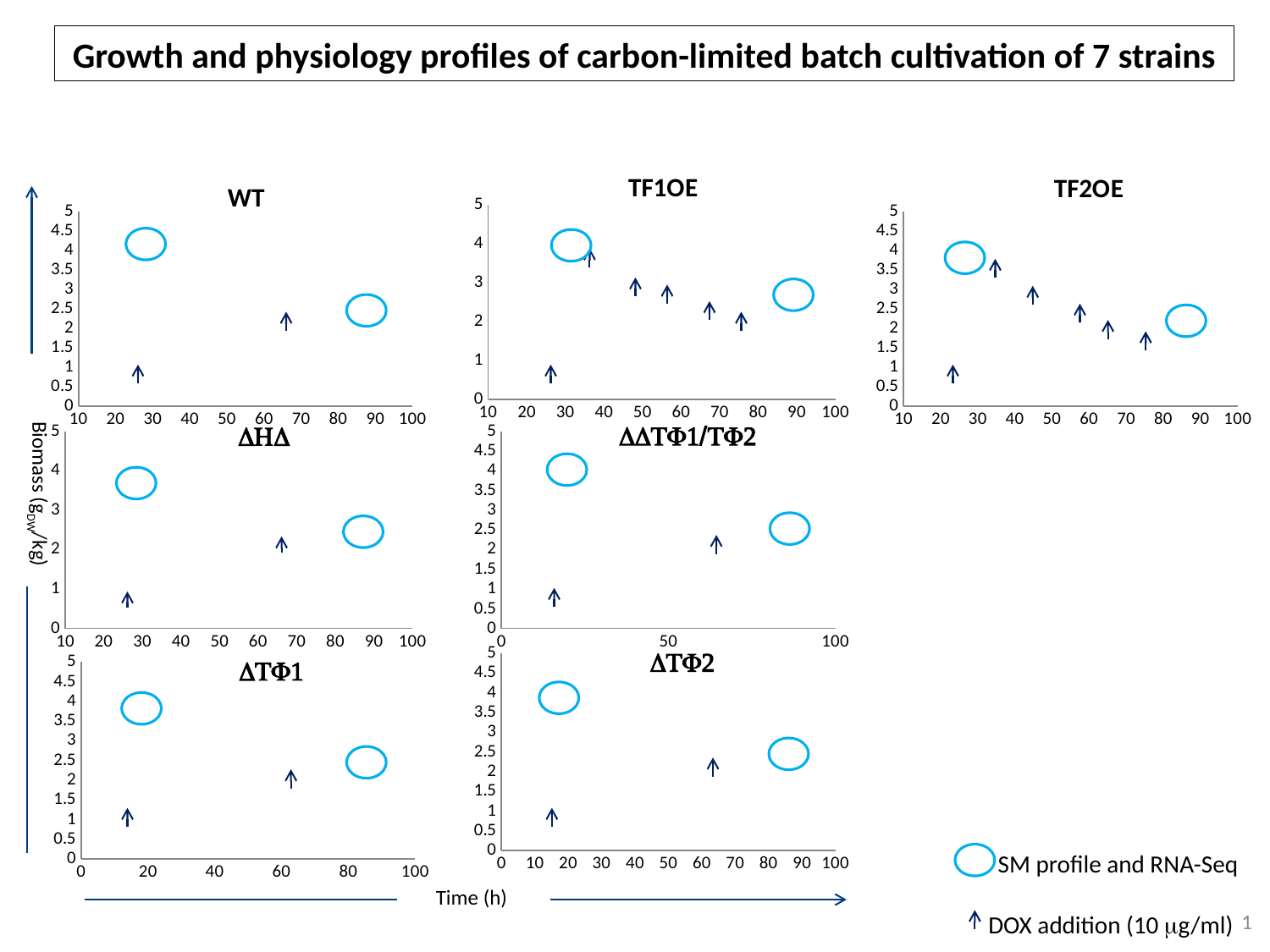

Growth and physiology profiles of carbon-limited batch cultivation of 7 strains
### Chart: TF1OE
| Category | BR85 | BR86 |
|---|---|---|
### Chart: WT
| Category | BR83 (WT) | BR84 (WT) |
|---|---|---|
### Chart: TF2OE
| Category | BR090 | BR089 |
|---|---|---|
### Chart: DHD
| Category | BR87 | BR88 |
|---|---|---|
### Chart: DDTF1/TF2
| Category | BR091 | BR092 |
|---|---|---|Biomass (gDW/kg)
### Chart: DTF2
| Category | BR095 | BR096 |
|---|---|---|
### Chart: DTF1
| Category | BR093 | BR094 |
|---|---|---|
SM profile and RNA-Seq
Time (h)
1
DOX addition (10 mg/ml)

## Slide 2
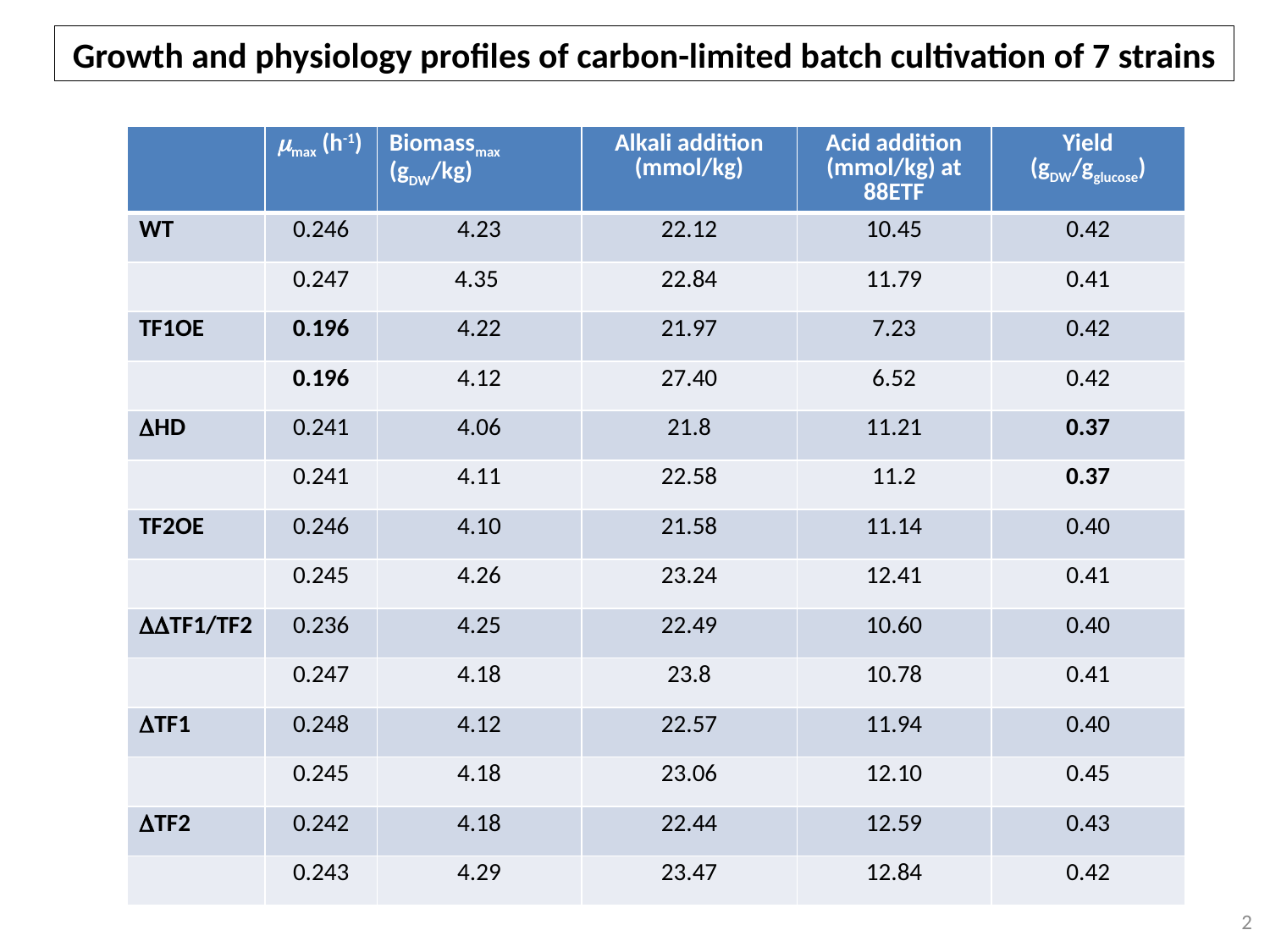

Growth and physiology profiles of carbon-limited batch cultivation of 7 strains
| | mmax (h-1) | Biomassmax (gDW/kg) | Alkali addition (mmol/kg) | Acid addition (mmol/kg) at 88ETF | Yield (gDW/gglucose) |
| --- | --- | --- | --- | --- | --- |
| WT | 0.246 | 4.23 | 22.12 | 10.45 | 0.42 |
| | 0.247 | 4.35 | 22.84 | 11.79 | 0.41 |
| TF1OE | 0.196 | 4.22 | 21.97 | 7.23 | 0.42 |
| | 0.196 | 4.12 | 27.40 | 6.52 | 0.42 |
| DHD | 0.241 | 4.06 | 21.8 | 11.21 | 0.37 |
| | 0.241 | 4.11 | 22.58 | 11.2 | 0.37 |
| TF2OE | 0.246 | 4.10 | 21.58 | 11.14 | 0.40 |
| | 0.245 | 4.26 | 23.24 | 12.41 | 0.41 |
| DDTF1/TF2 | 0.236 | 4.25 | 22.49 | 10.60 | 0.40 |
| | 0.247 | 4.18 | 23.8 | 10.78 | 0.41 |
| DTF1 | 0.248 | 4.12 | 22.57 | 11.94 | 0.40 |
| | 0.245 | 4.18 | 23.06 | 12.10 | 0.45 |
| DTF2 | 0.242 | 4.18 | 22.44 | 12.59 | 0.43 |
| | 0.243 | 4.29 | 23.47 | 12.84 | 0.42 |
2

## Slide 3
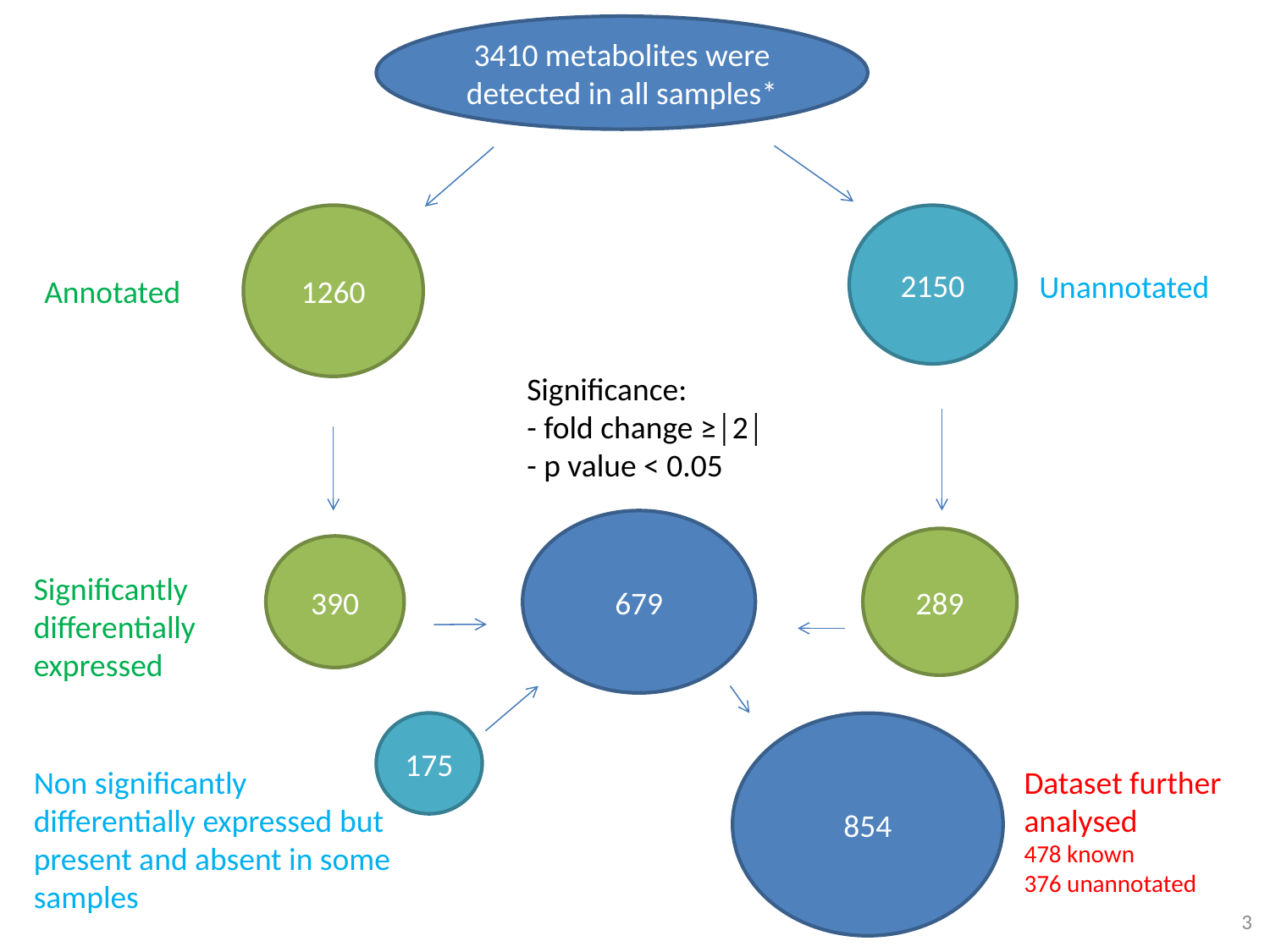

3410 metabolites were detected in all samples*
1260
2150
Unannotated
Annotated
Significance:
- fold change ≥│2│
- p value < 0.05
679
289
390
Significantly differentially expressed
175
854
Non significantly differentially expressed but
present and absent in some samples
Dataset further analysed
478 known
376 unannotated
3

## Slide 4
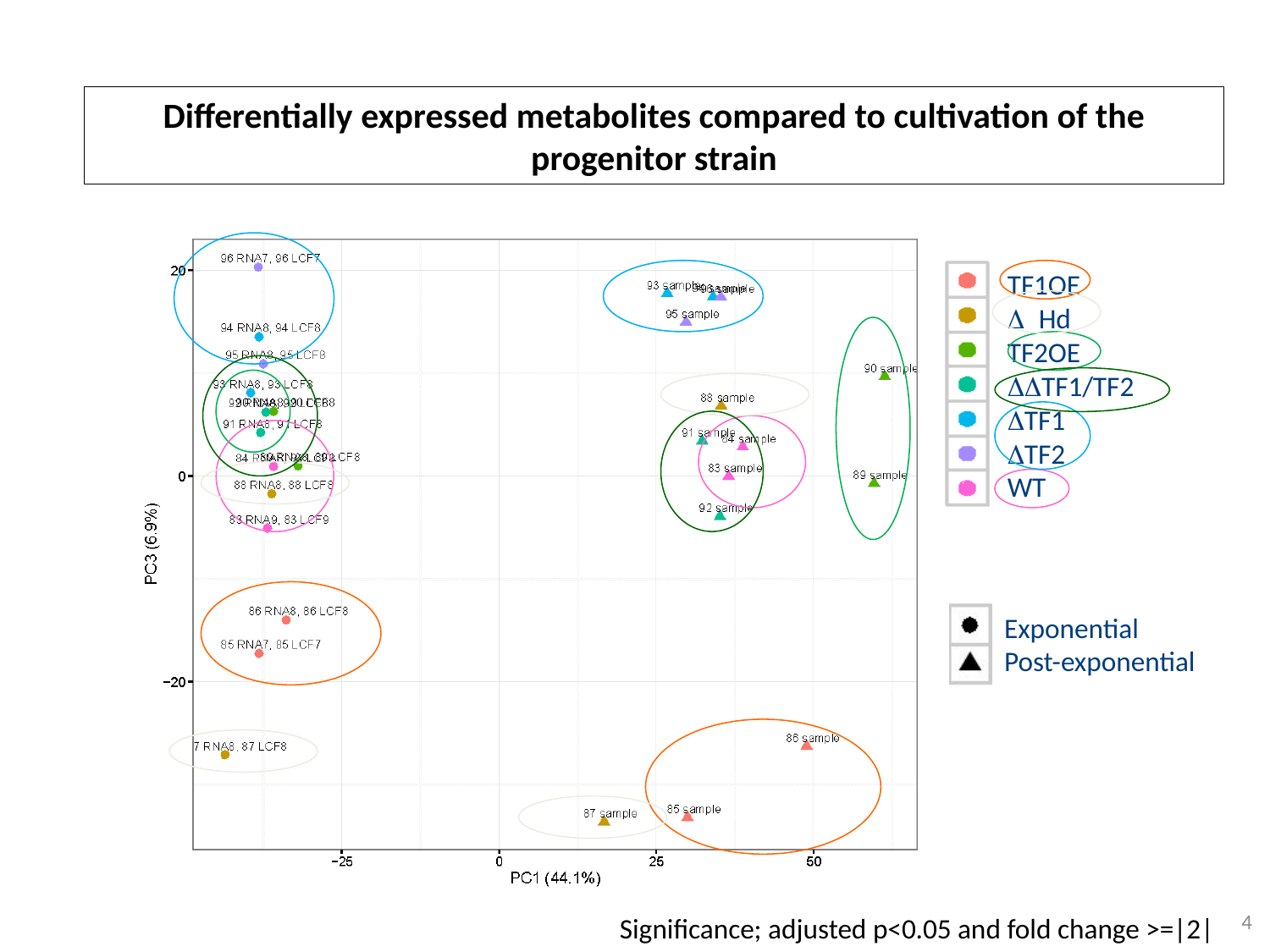

Differentially expressed metabolites compared to cultivation of the progenitor strain
TF1OE
D Hd
TF2OE
DDTF1/TF2
DTF1
DTF2
WT
Exponential
Post-exponential
4
Significance; adjusted p<0.05 and fold change >=|2|

## Slide 5
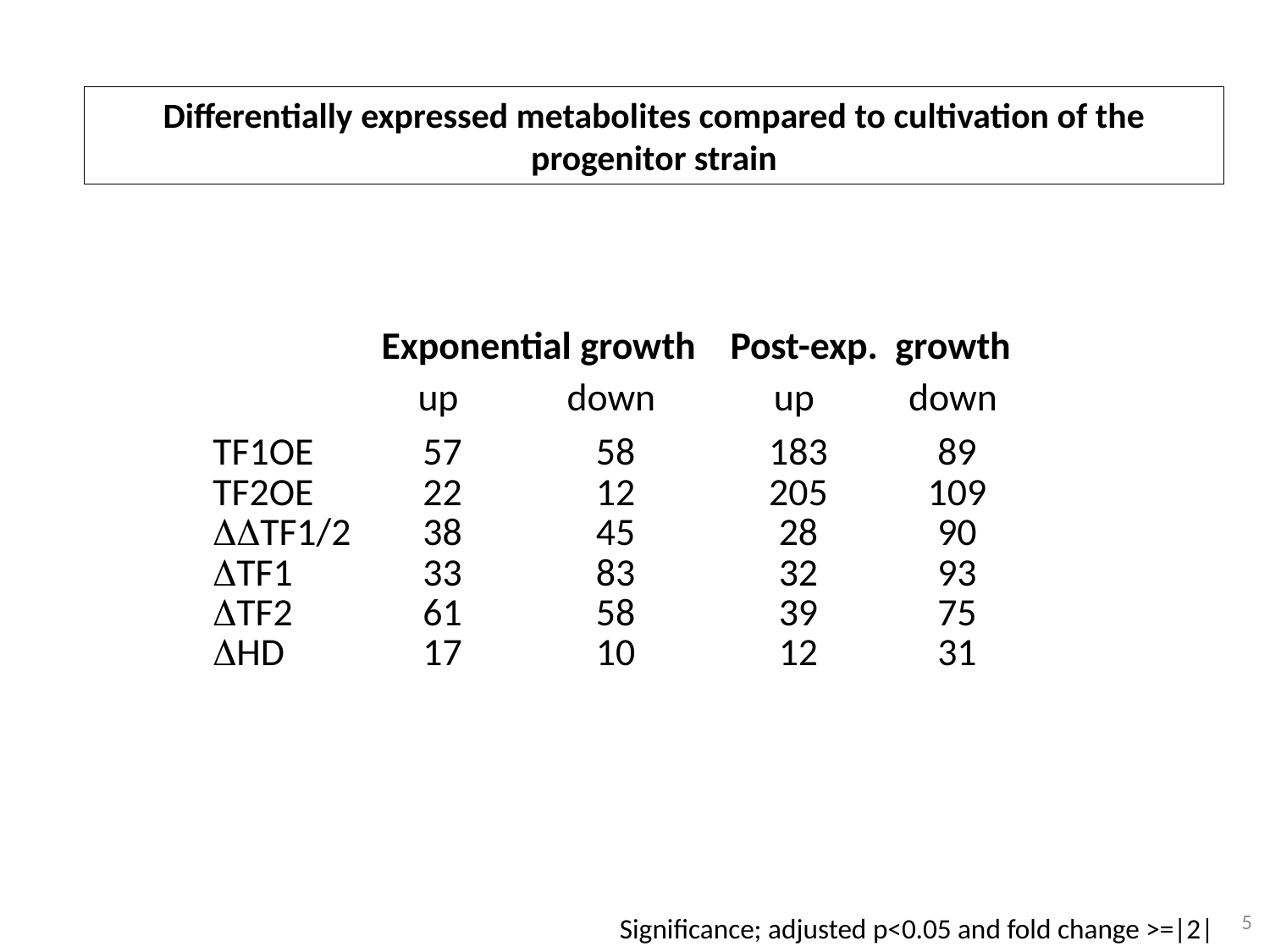

Differentially expressed metabolites compared to cultivation of the progenitor strain
| | | | | |
| --- | --- | --- | --- | --- |
| | Exponential growth | | Post-exp. growth | |
| | up | down | up | down |
| TF1OE | 57 | 58 | 183 | 89 |
| TF2OE | 22 | 12 | 205 | 109 |
| DDTF1/2 | 38 | 45 | 28 | 90 |
| DTF1 | 33 | 83 | 32 | 93 |
| DTF2 | 61 | 58 | 39 | 75 |
| DHD | 17 | 10 | 12 | 31 |
5
Significance; adjusted p<0.05 and fold change >=|2|

## Slide 6
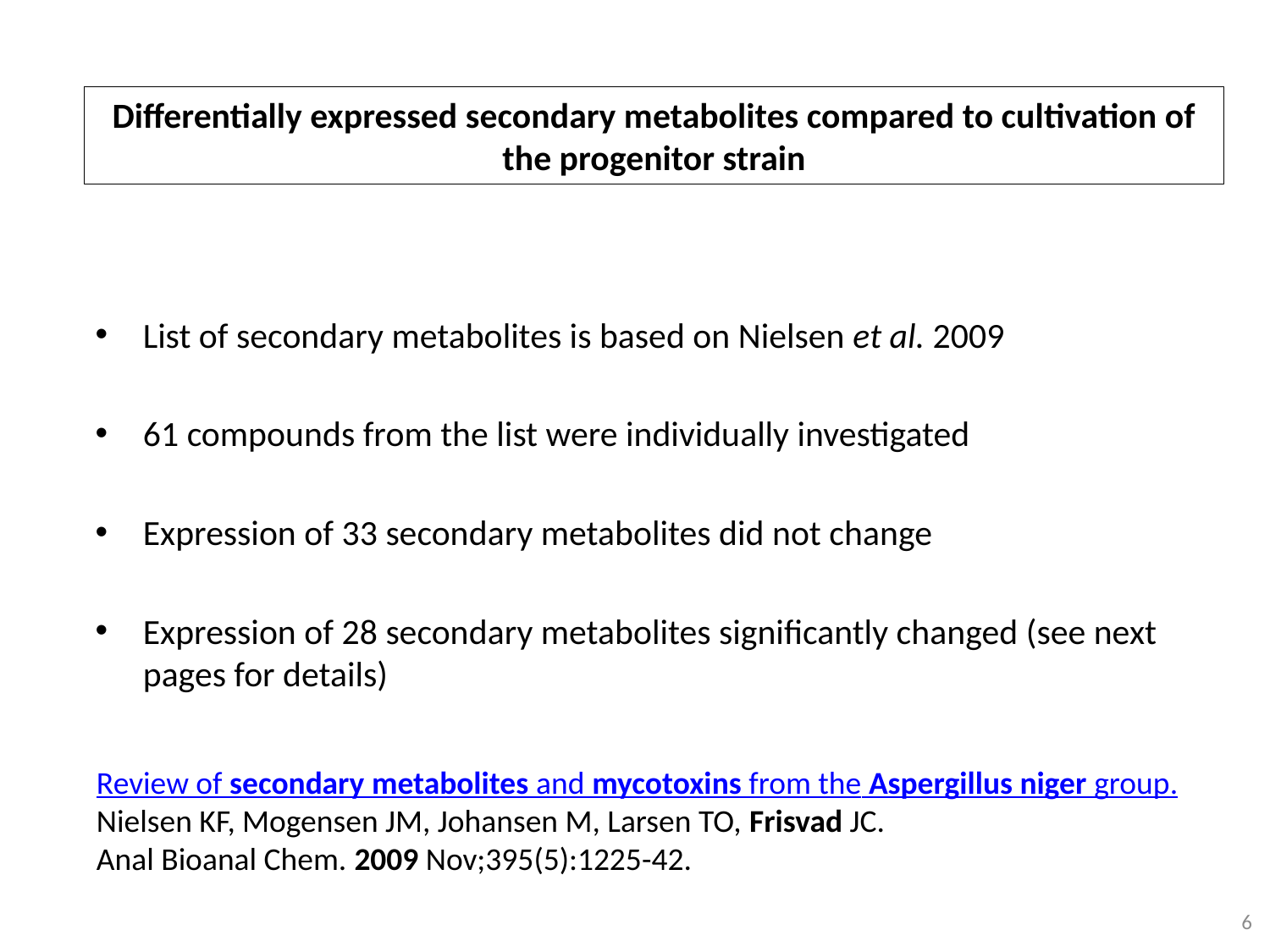

Differentially expressed secondary metabolites compared to cultivation of the progenitor strain
List of secondary metabolites is based on Nielsen et al. 2009
61 compounds from the list were individually investigated
Expression of 33 secondary metabolites did not change
Expression of 28 secondary metabolites significantly changed (see next pages for details)
Review of secondary metabolites and mycotoxins from the Aspergillus niger group.
Nielsen KF, Mogensen JM, Johansen M, Larsen TO, Frisvad JC.
Anal Bioanal Chem. 2009 Nov;395(5):1225-42.
6

## Slide 7
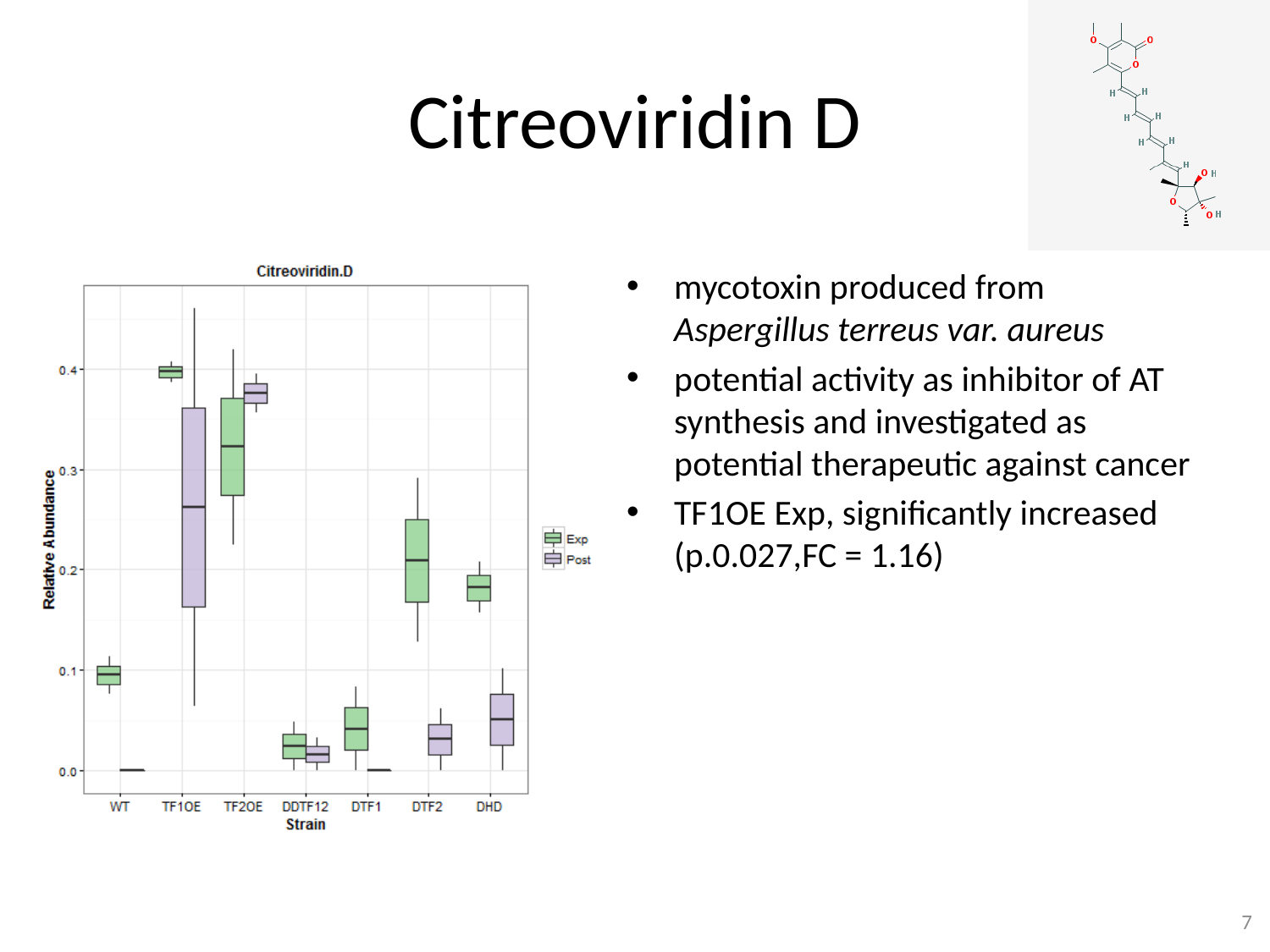

Citreoviridin D
mycotoxin produced from Aspergillus terreus var. aureus
potential activity as inhibitor of AT synthesis and investigated as potential therapeutic against cancer
TF1OE Exp, significantly increased (p.0.027,FC = 1.16)
7

## Slide 8
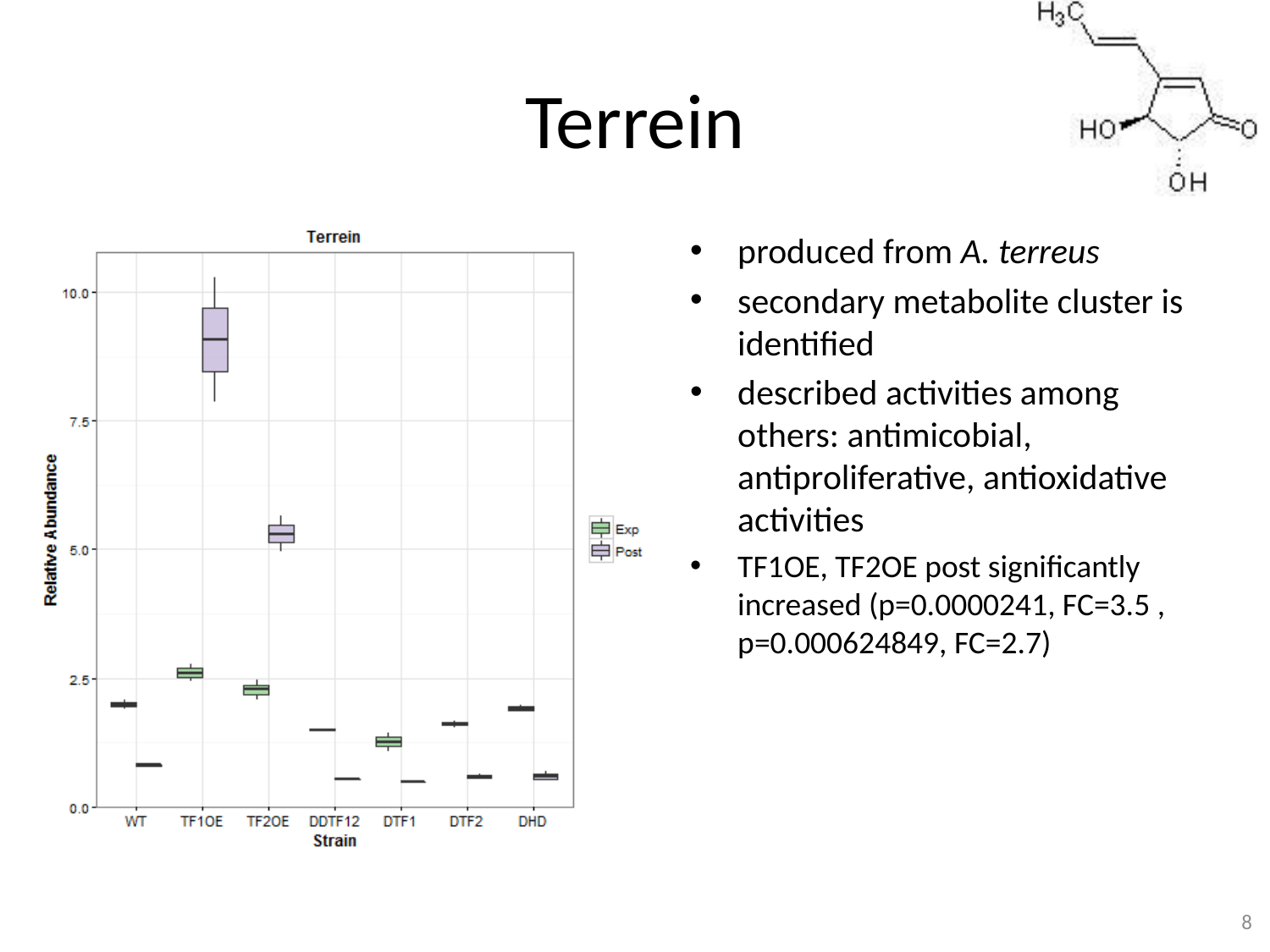

Terrein
produced from A. terreus
secondary metabolite cluster is identified
described activities among others: antimicobial, antiproliferative, antioxidative activities
TF1OE, TF2OE post significantly increased (p=0.0000241, FC=3.5 , p=0.000624849, FC=2.7)
8

## Slide 9
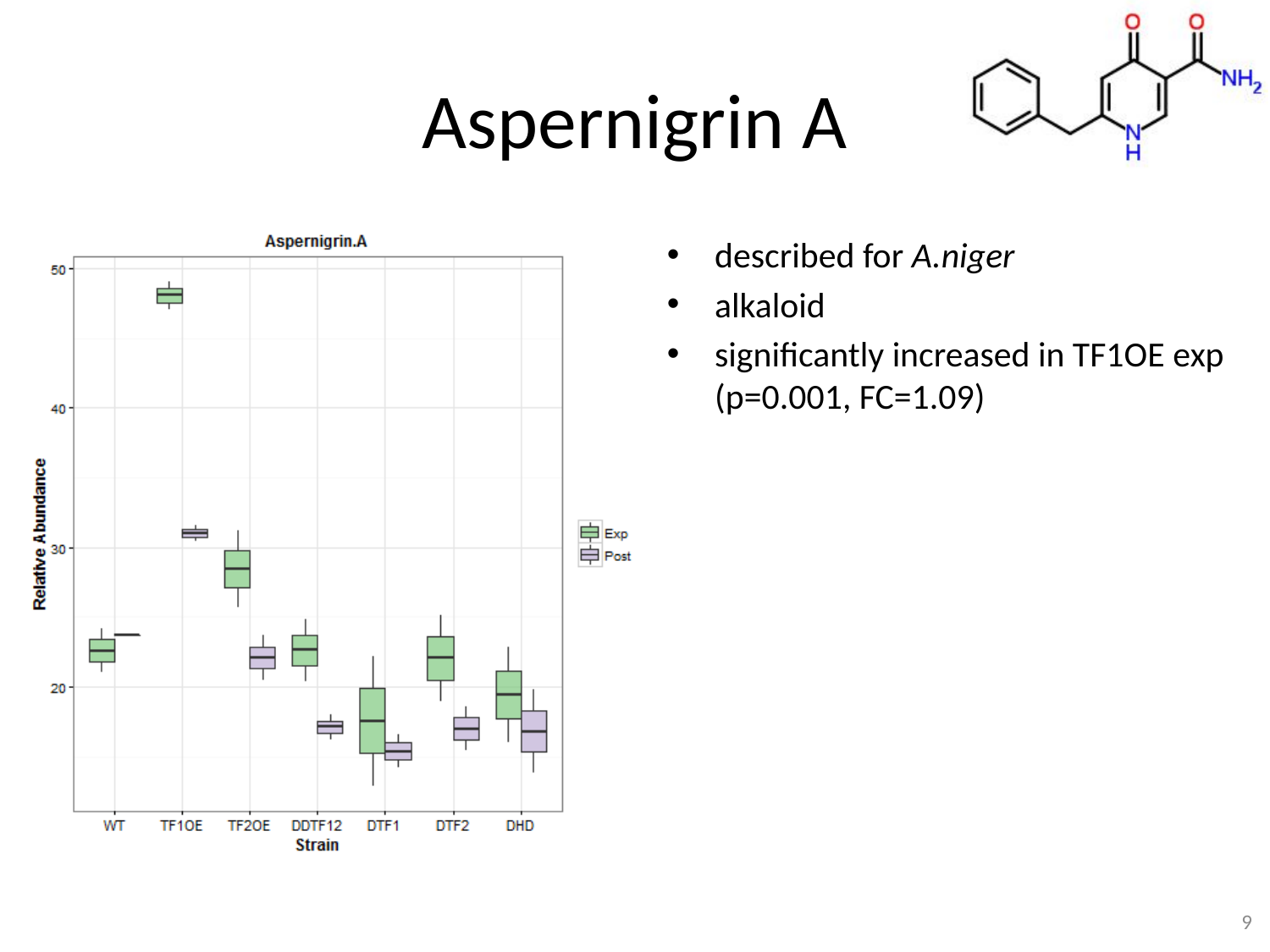

# Aspernigrin A
described for A.niger
alkaloid
significantly increased in TF1OE exp (p=0.001, FC=1.09)
9

## Slide 10
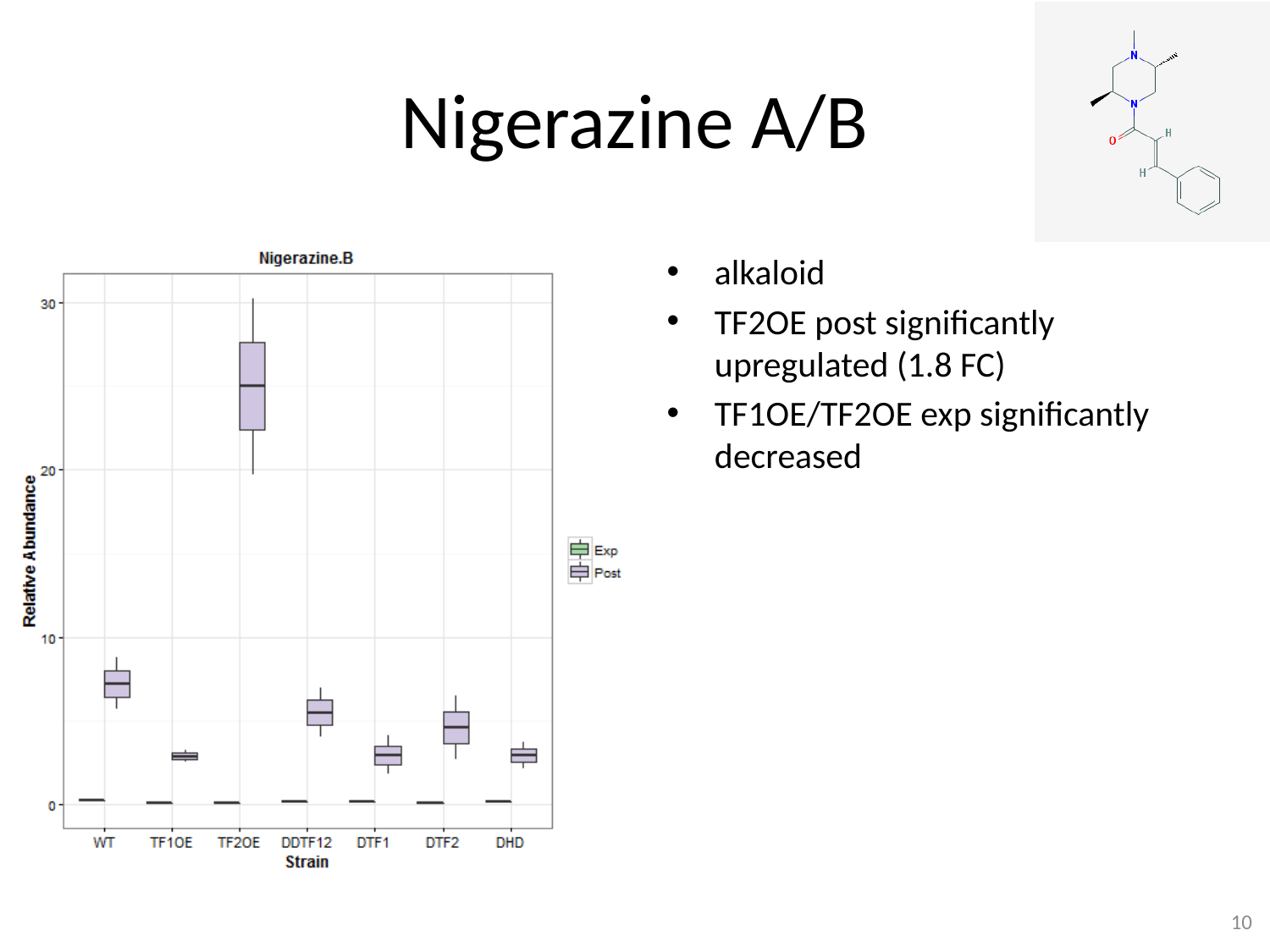

# Nigerazine A/B
alkaloid
TF2OE post significantly upregulated (1.8 FC)
TF1OE/TF2OE exp significantly decreased
10

## Slide 11
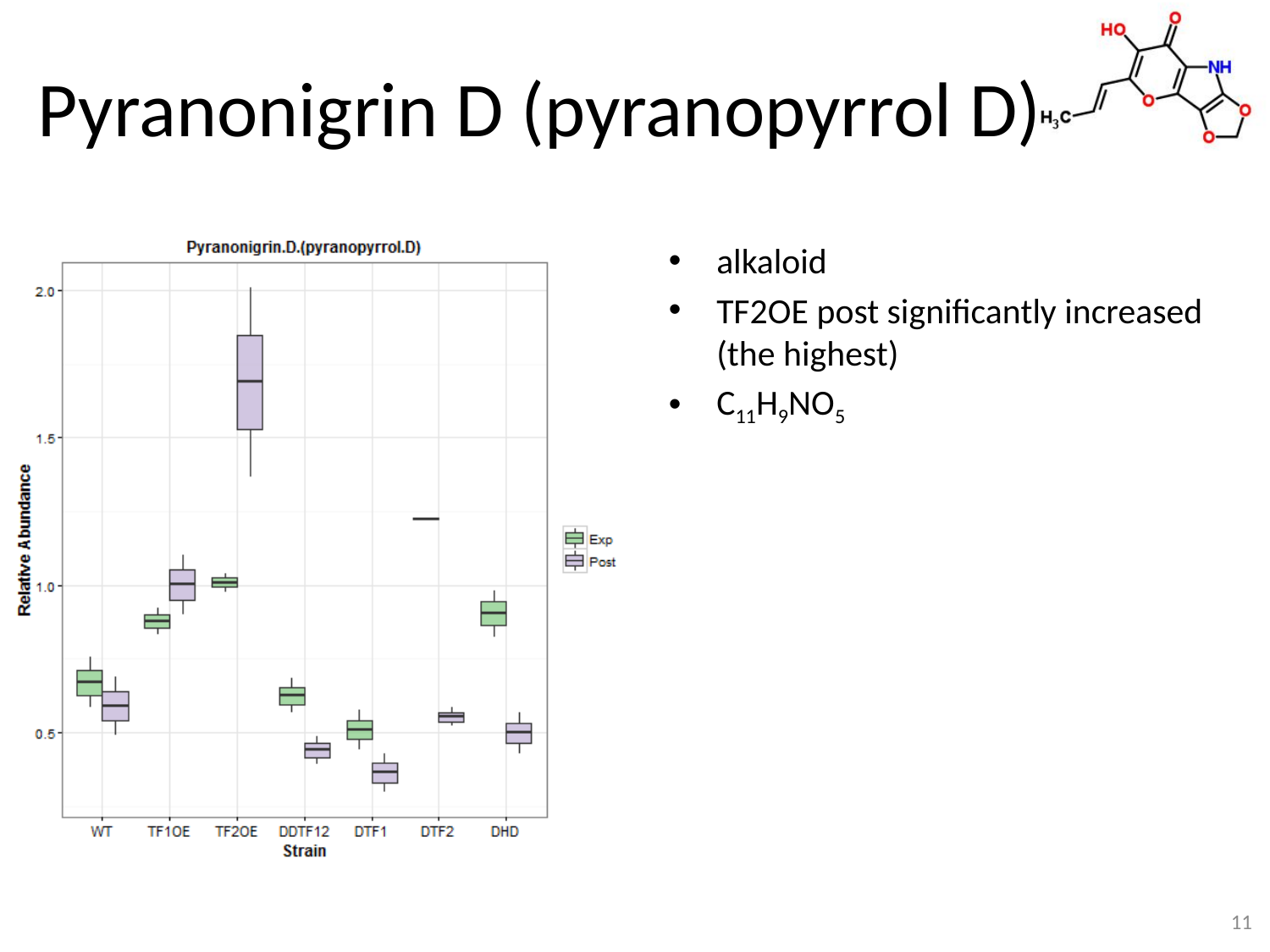

# Pyranonigrin D (pyranopyrrol D)
alkaloid
TF2OE post significantly increased (the highest)
C11H9NO5
11

## Slide 12
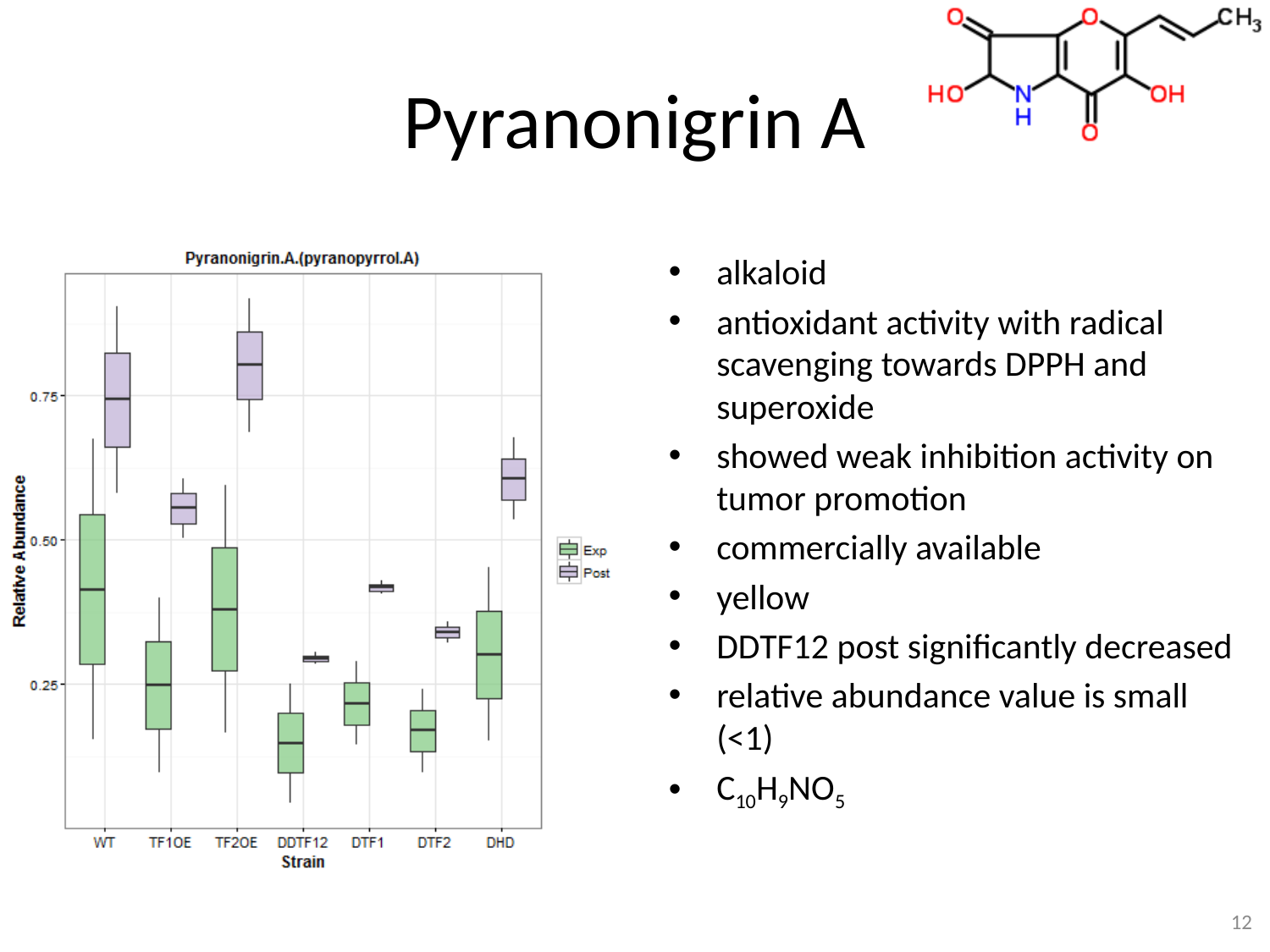

# Pyranonigrin A
alkaloid
antioxidant activity with radical scavenging towards DPPH and superoxide
showed weak inhibition activity on tumor promotion
commercially available
yellow
DDTF12 post significantly decreased
relative abundance value is small (<1)
C10H9NO5
12

## Slide 13
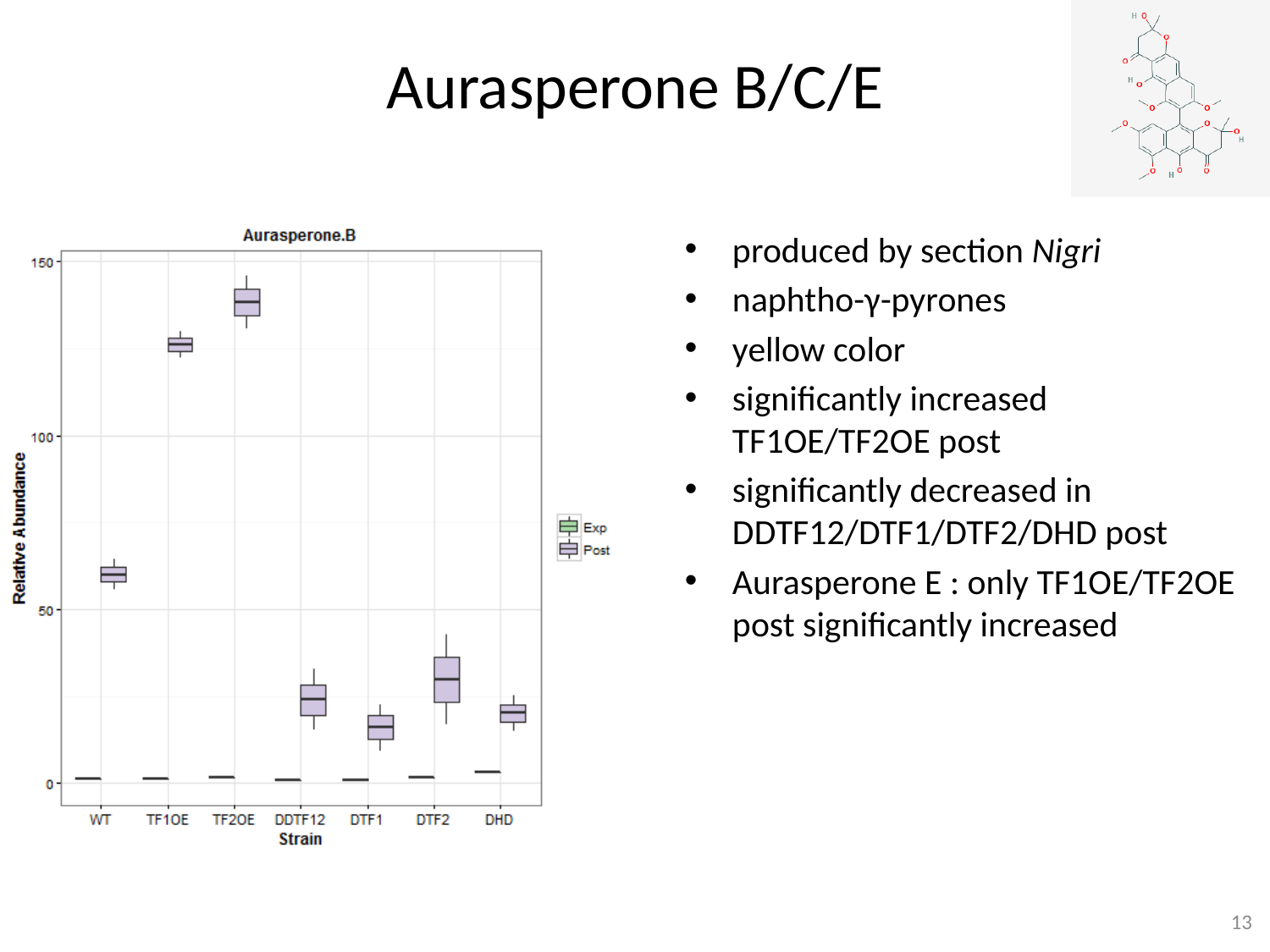

# Aurasperone B/C/E
produced by section Nigri
naphtho-γ-pyrones
yellow color
significantly increased TF1OE/TF2OE post
significantly decreased in DDTF12/DTF1/DTF2/DHD post
Aurasperone E : only TF1OE/TF2OE post significantly increased
13

## Slide 14
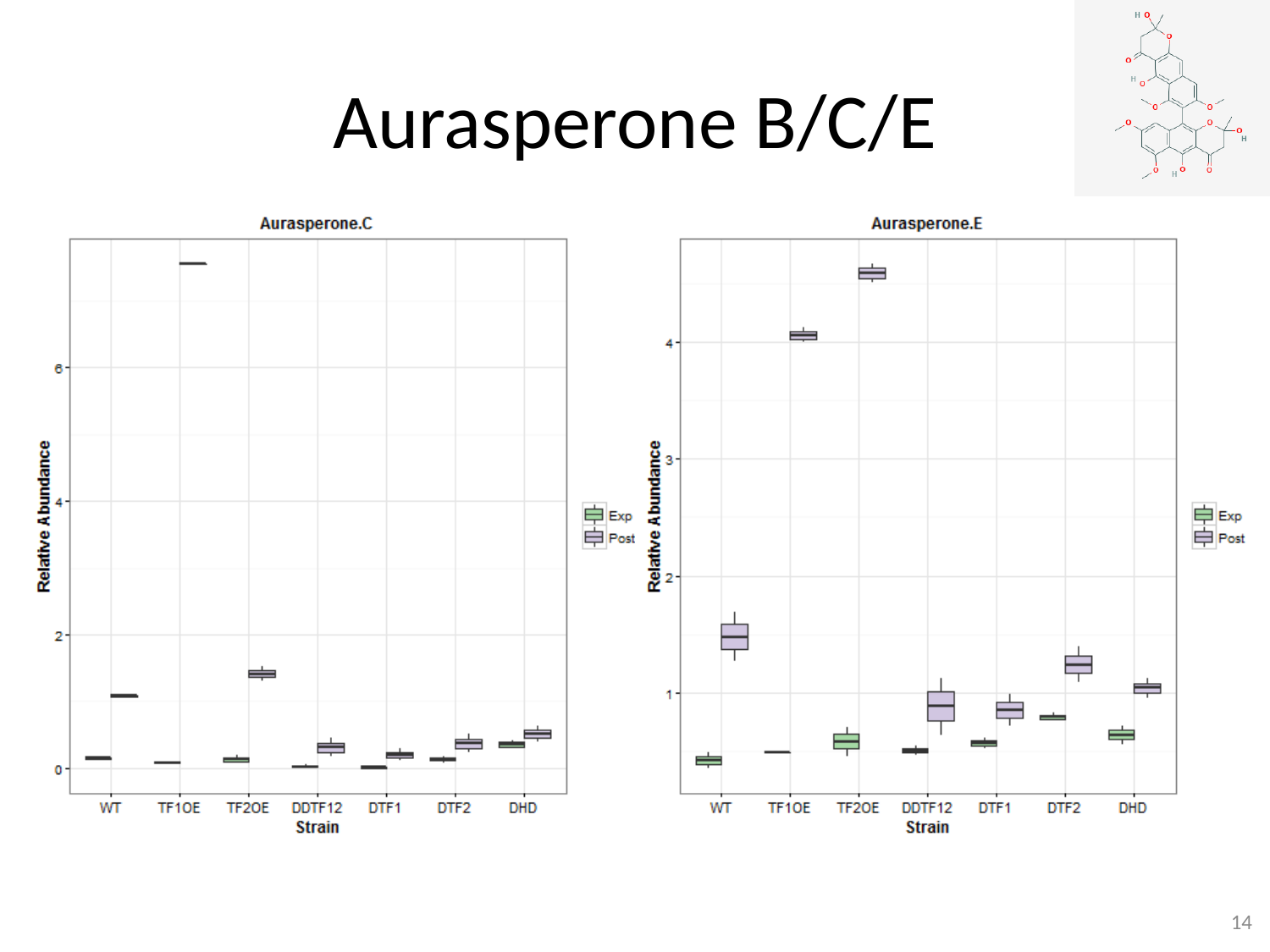

# Aurasperone B/C/E
14

## Slide 15
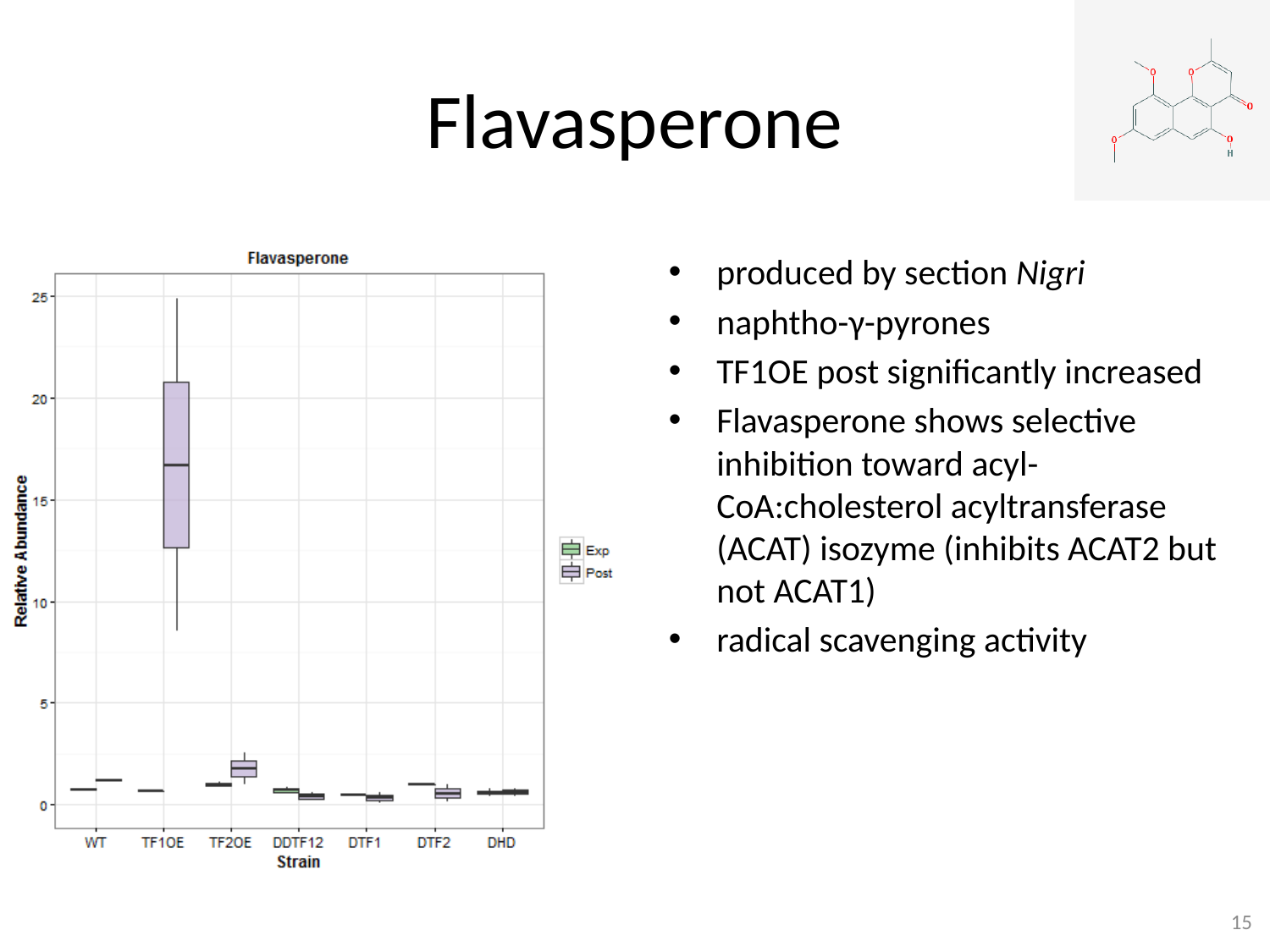

# Flavasperone
produced by section Nigri
naphtho-γ-pyrones
TF1OE post significantly increased
Flavasperone shows selective inhibition toward acyl-CoA:cholesterol acyltransferase (ACAT) isozyme (inhibits ACAT2 but not ACAT1)
radical scavenging activity
15

## Slide 16
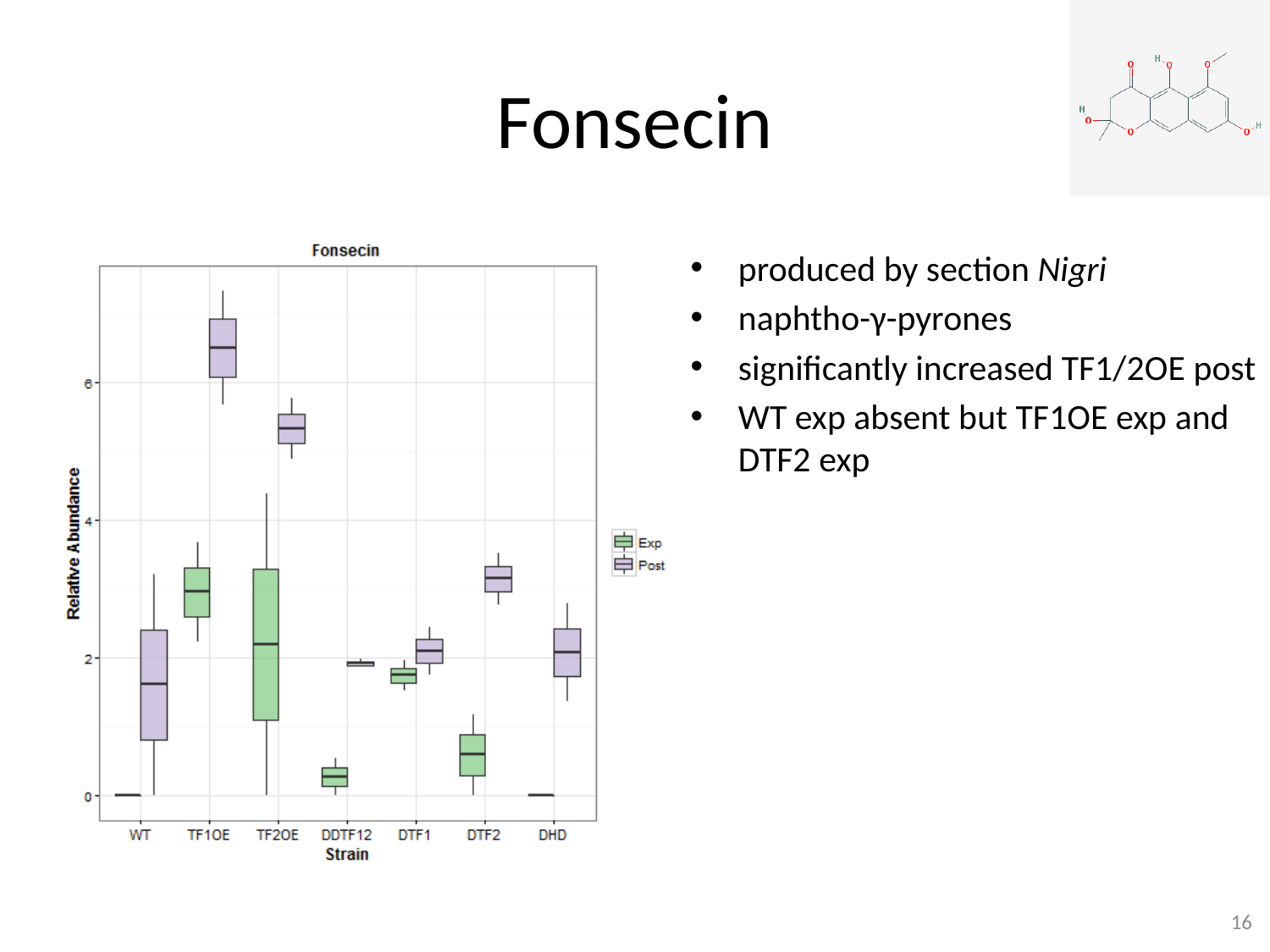

# Fonsecin
produced by section Nigri
naphtho-γ-pyrones
significantly increased TF1/2OE post
WT exp absent but TF1OE exp and DTF2 exp
16

## Slide 17
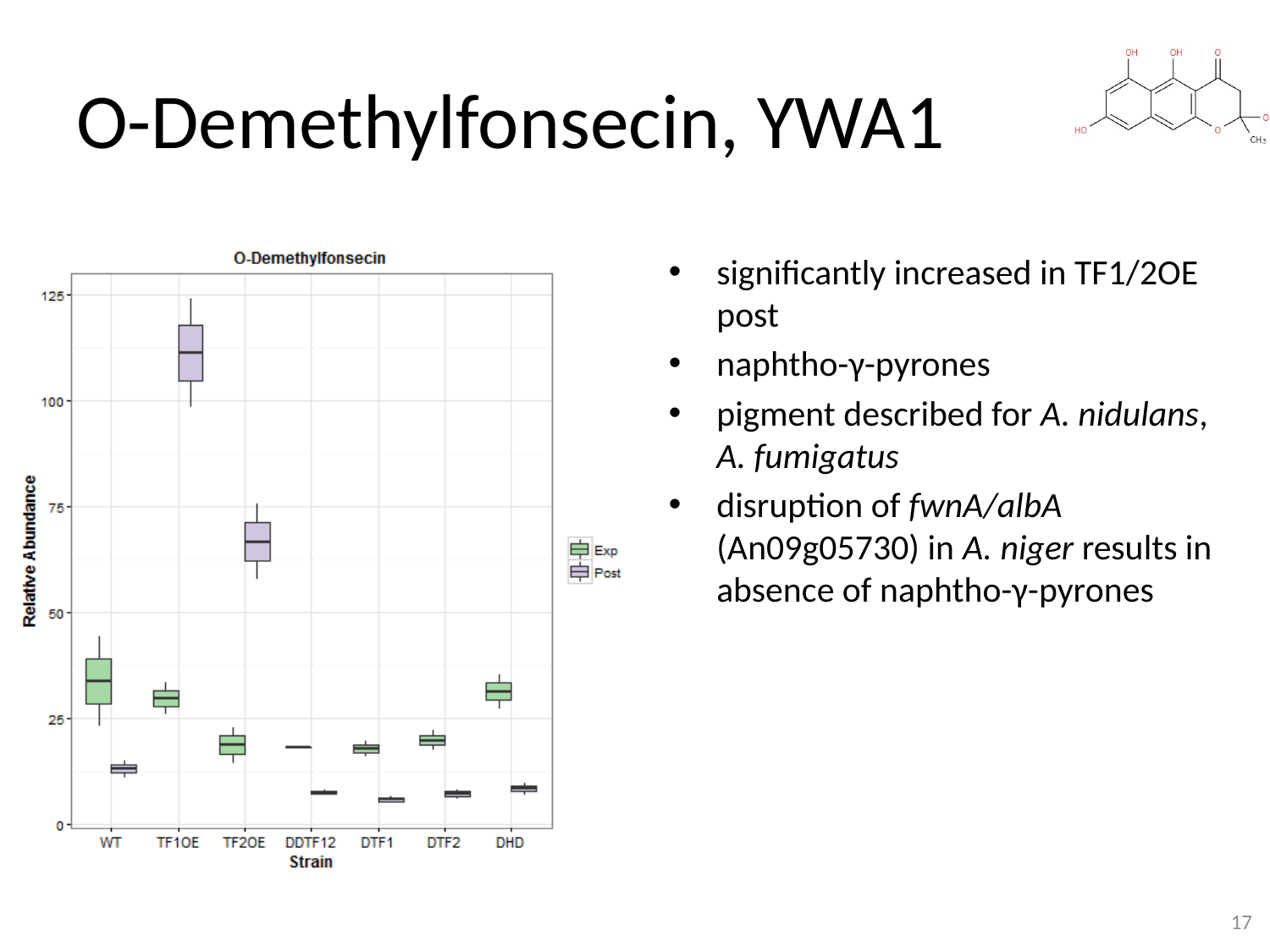

# O-Demethylfonsecin, YWA1
significantly increased in TF1/2OE post
naphtho-γ-pyrones
pigment described for A. nidulans, A. fumigatus
disruption of fwnA/albA (An09g05730) in A. niger results in absence of naphtho-γ-pyrones
17

## Slide 18
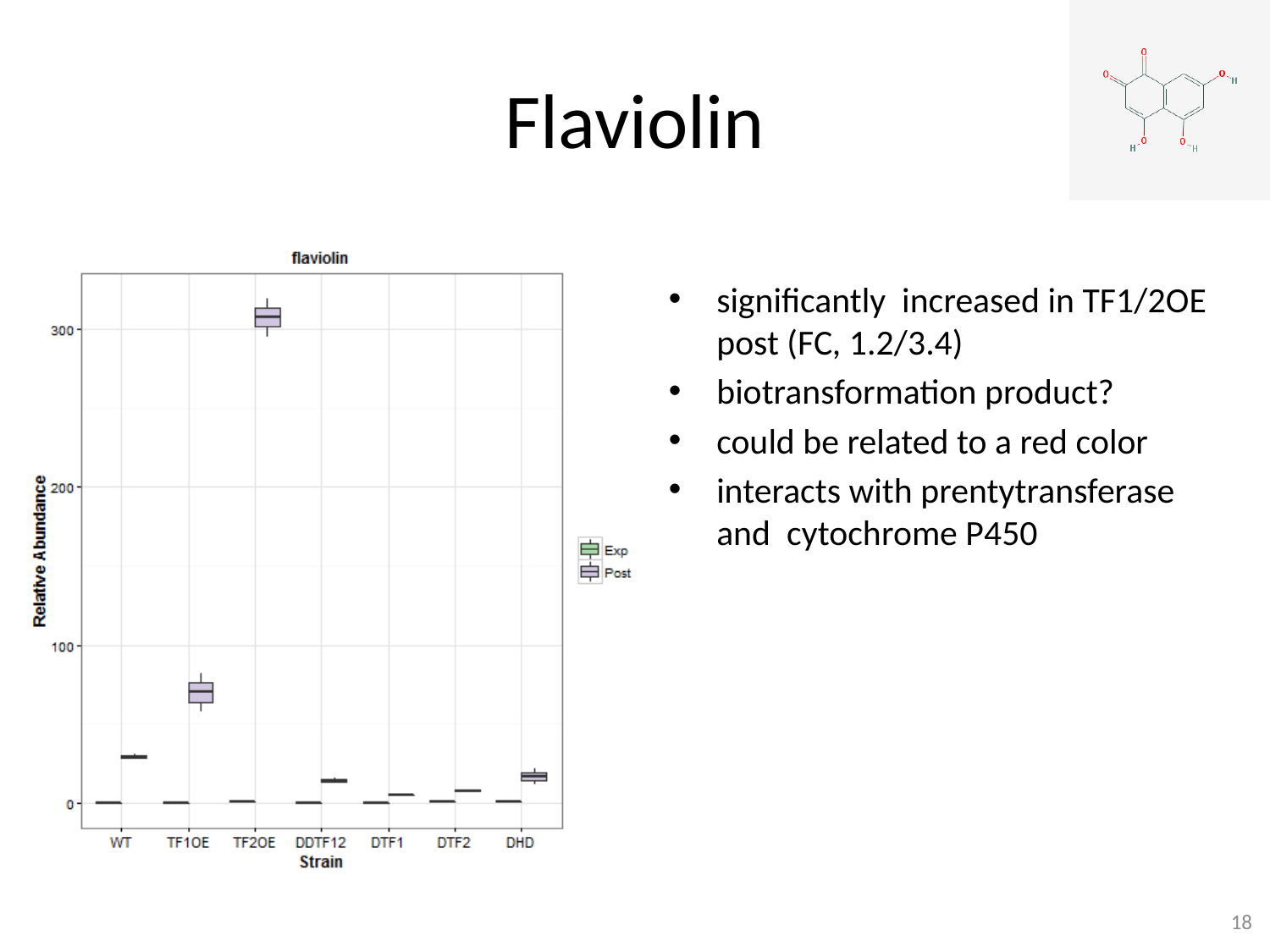

# Flaviolin
significantly increased in TF1/2OE post (FC, 1.2/3.4)
biotransformation product?
could be related to a red color
interacts with prentytransferase and cytochrome P450
18

## Slide 19
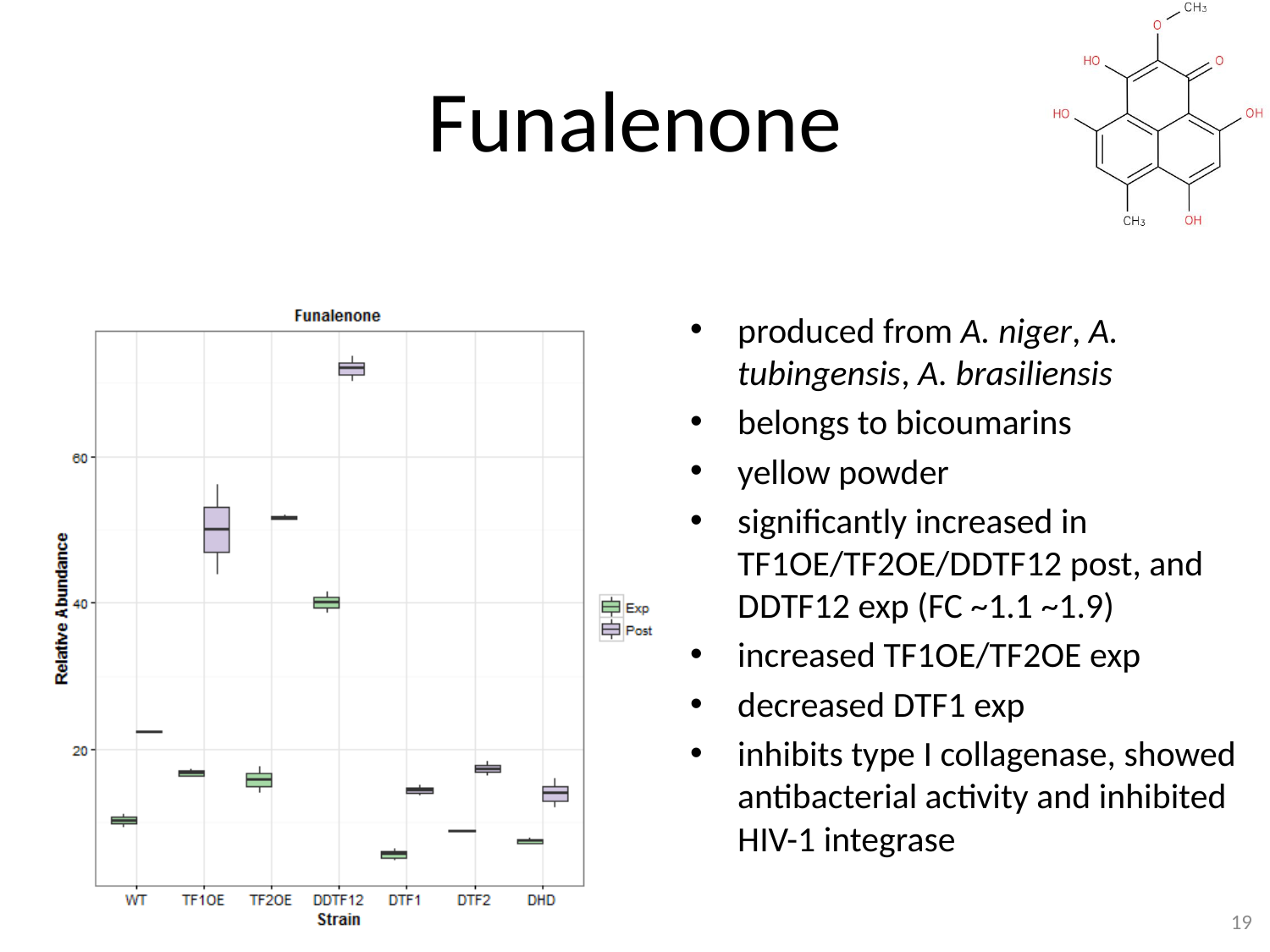

# Funalenone
produced from A. niger, A. tubingensis, A. brasiliensis
belongs to bicoumarins
yellow powder
significantly increased in TF1OE/TF2OE/DDTF12 post, and DDTF12 exp (FC ~1.1 ~1.9)
increased TF1OE/TF2OE exp
decreased DTF1 exp
inhibits type I collagenase, showed antibacterial activity and inhibited HIV-1 integrase
19

## Slide 20
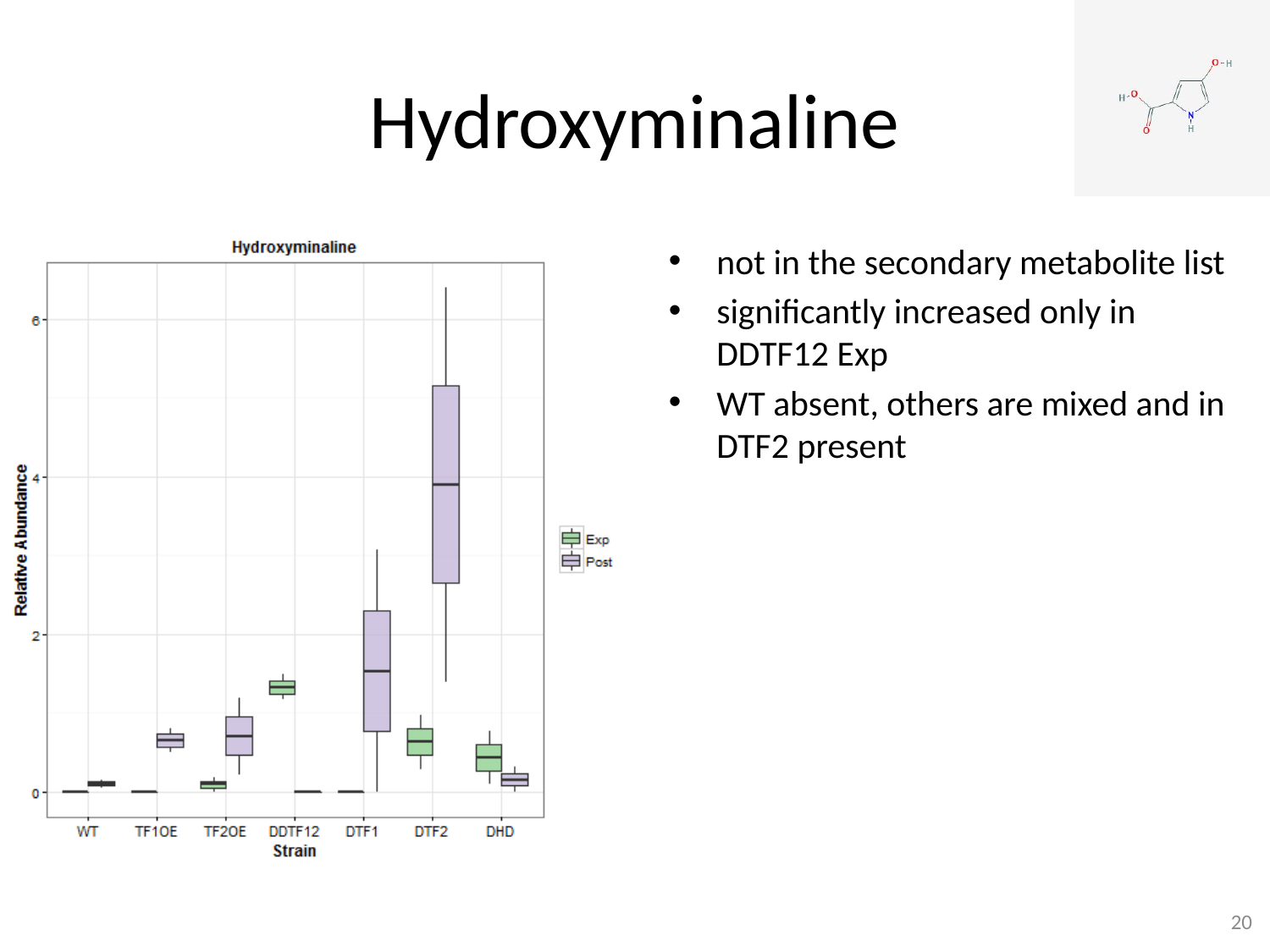

# Hydroxyminaline
not in the secondary metabolite list
significantly increased only in DDTF12 Exp
WT absent, others are mixed and in DTF2 present
20

## Slide 21
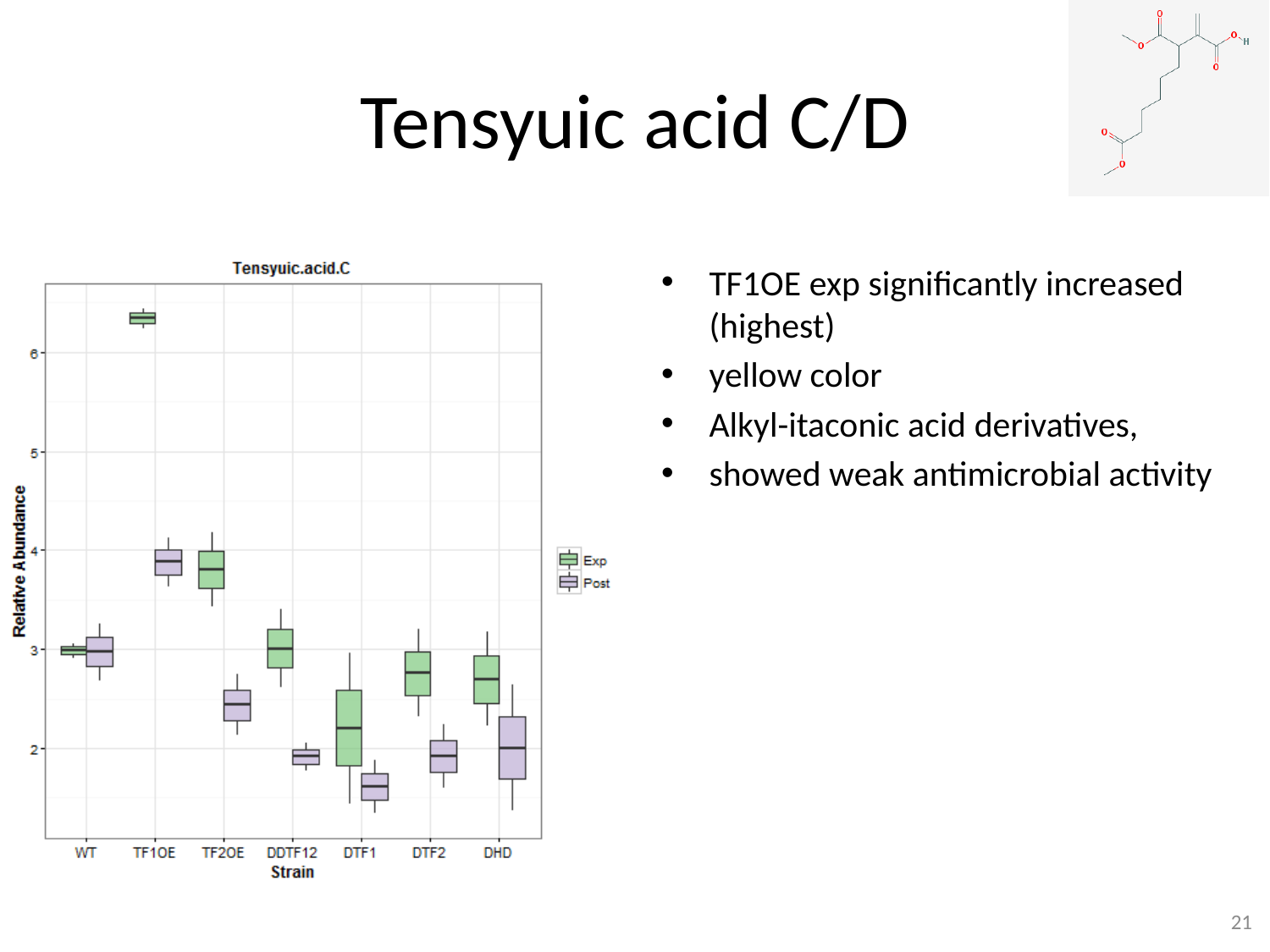

# Tensyuic acid C/D
TF1OE exp significantly increased (highest)
yellow color
Alkyl-itaconic acid derivatives,
showed weak antimicrobial activity
21

## Slide 22
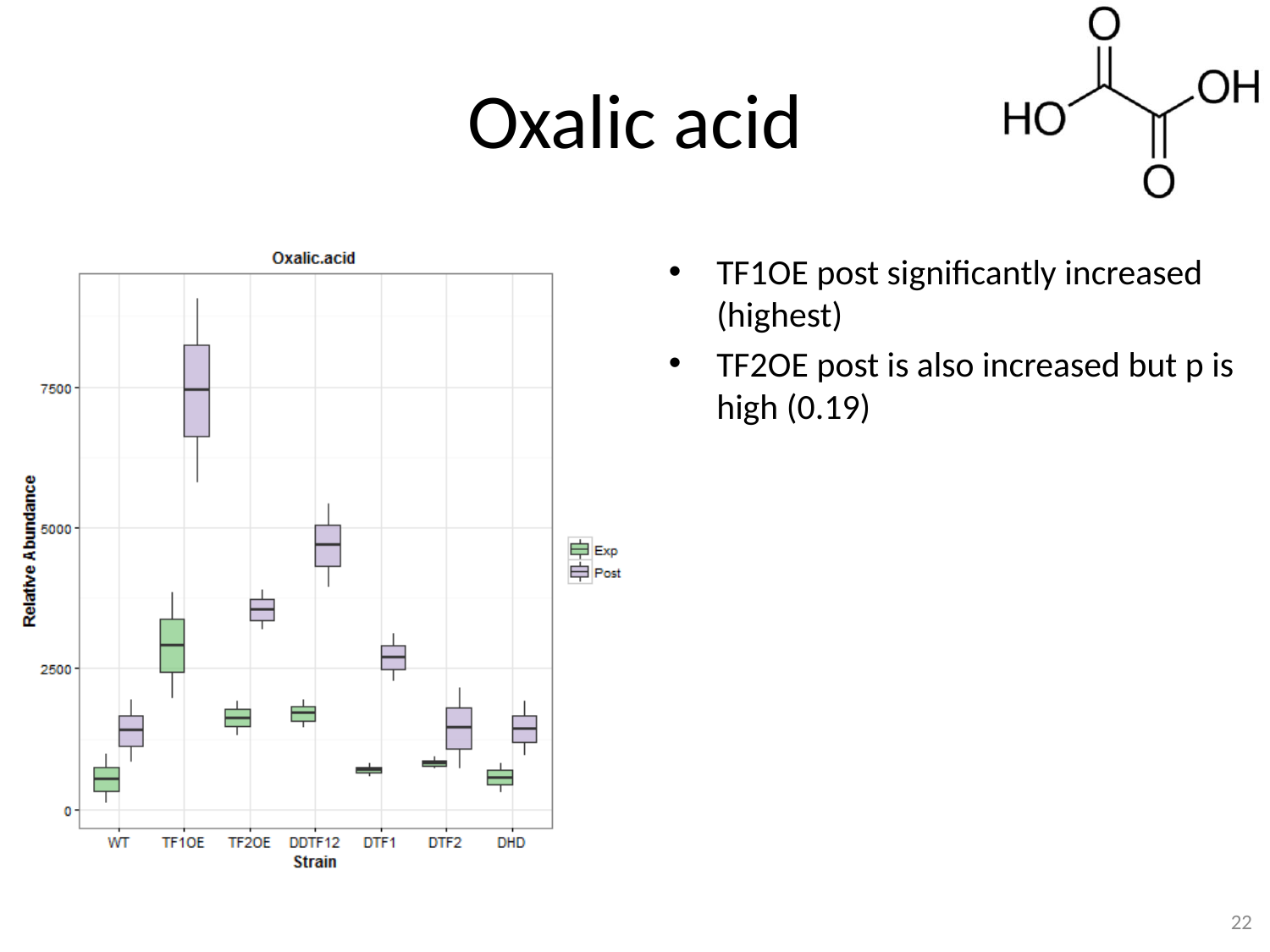

# Oxalic acid
TF1OE post significantly increased (highest)
TF2OE post is also increased but p is high (0.19)
22

## Slide 23
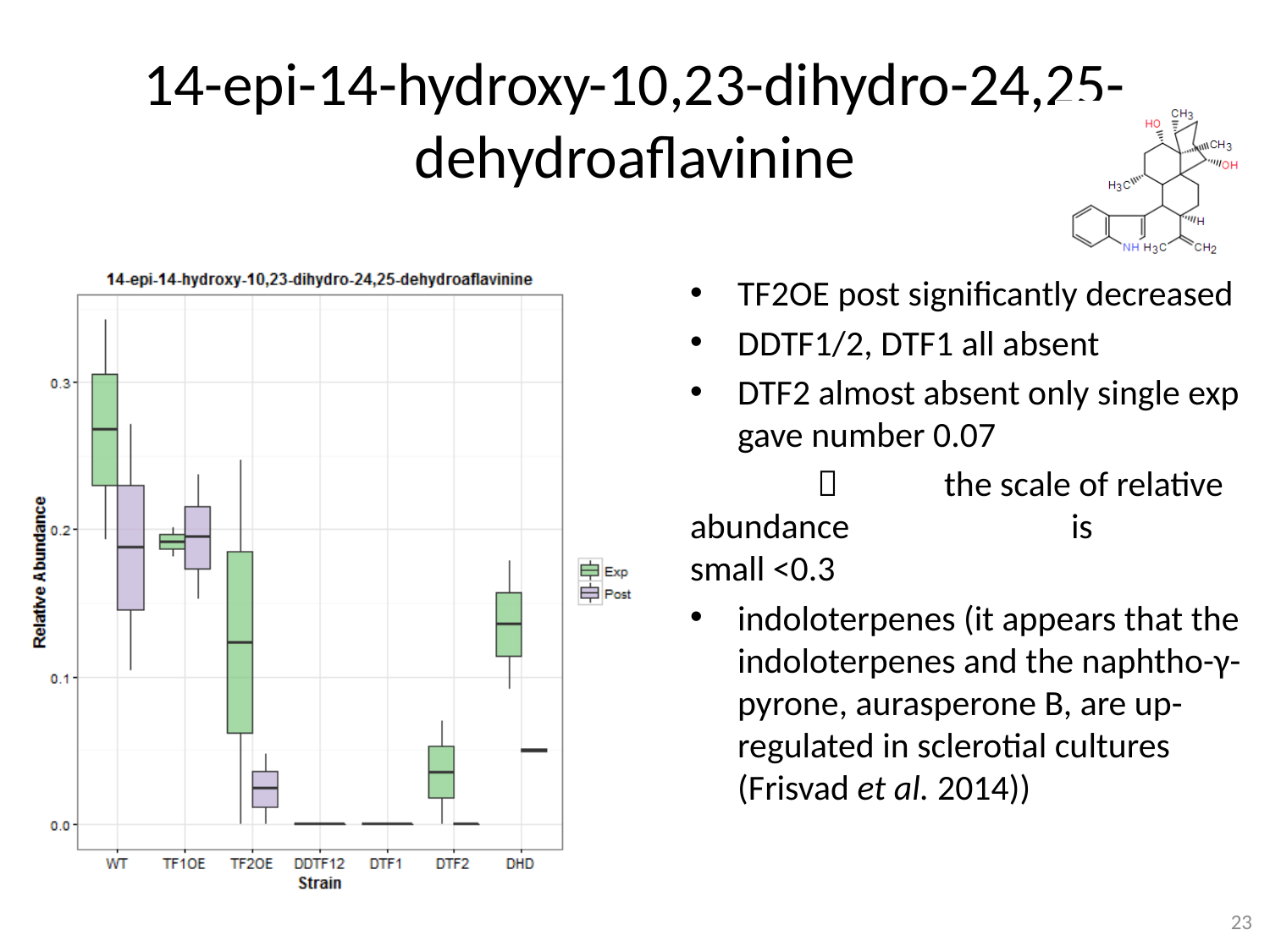

# 14-epi-14-hydroxy-10,23-dihydro-24,25-dehydroaflavinine
TF2OE post significantly decreased
DDTF1/2, DTF1 all absent
DTF2 almost absent only single exp gave number 0.07
 	 	the scale of relative abundance 		is	small <0.3
indoloterpenes (it appears that the indoloterpenes and the naphtho-γ-pyrone, aurasperone B, are up-regulated in sclerotial cultures (Frisvad et al. 2014))
23

## Slide 24
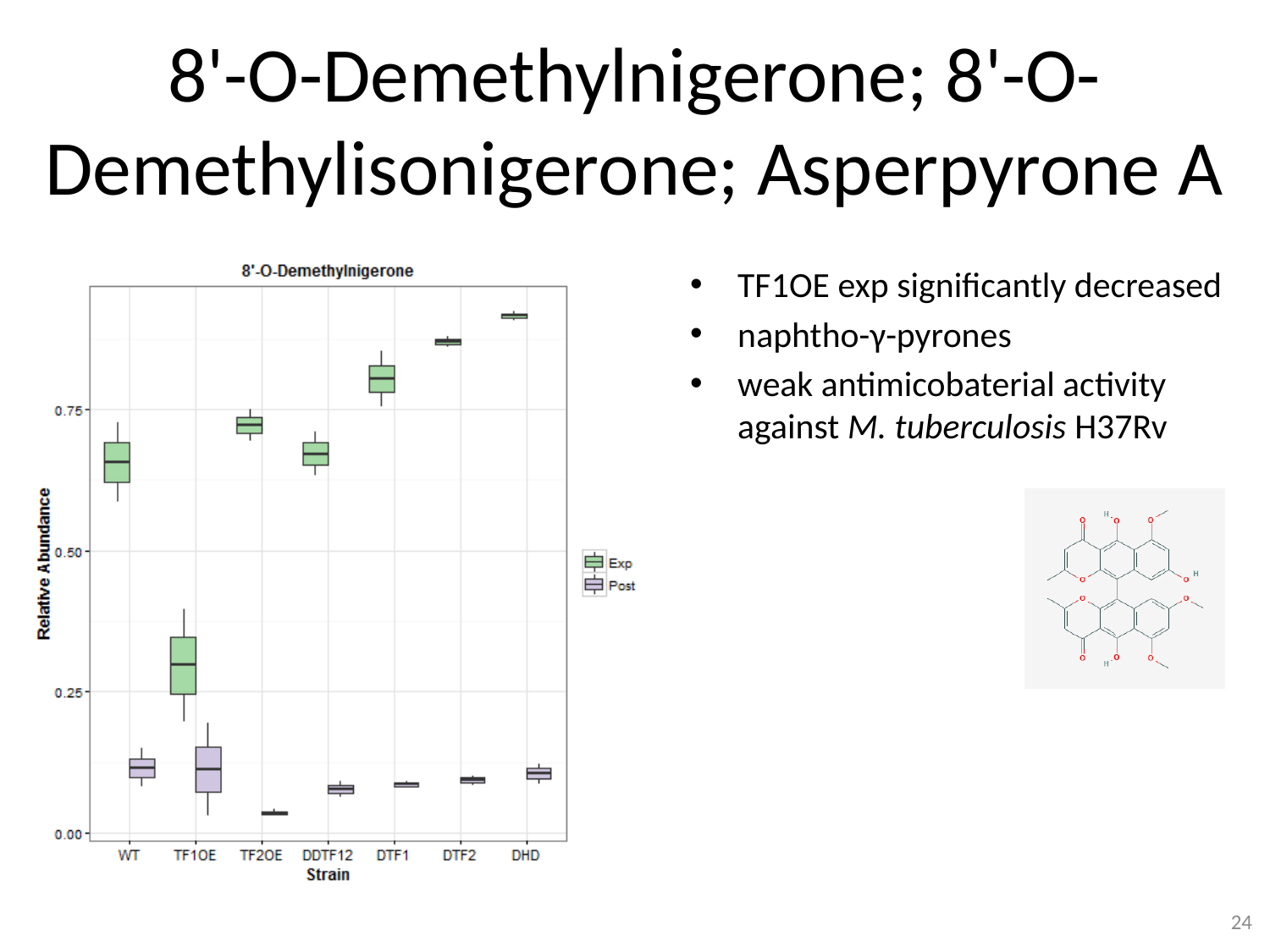

# 8'-O-Demethylnigerone; 8'-O-Demethylisonigerone; Asperpyrone A
TF1OE exp significantly decreased
naphtho-γ-pyrones
weak antimicobaterial activity against M. tuberculosis H37Rv
24

## Slide 25
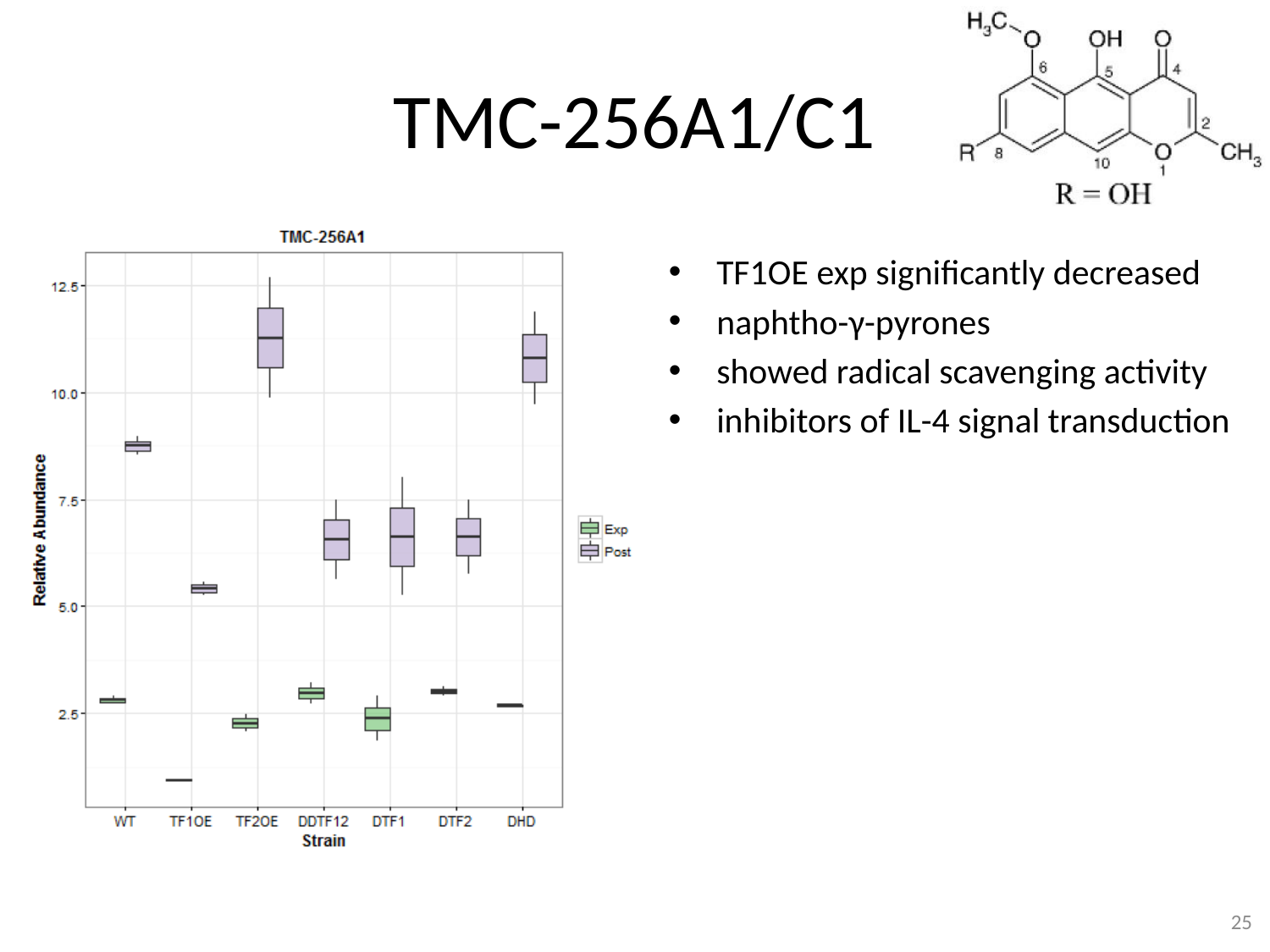

# TMC-256A1/C1
TF1OE exp significantly decreased
naphtho-γ-pyrones
showed radical scavenging activity
inhibitors of IL-4 signal transduction
25

## Slide 26
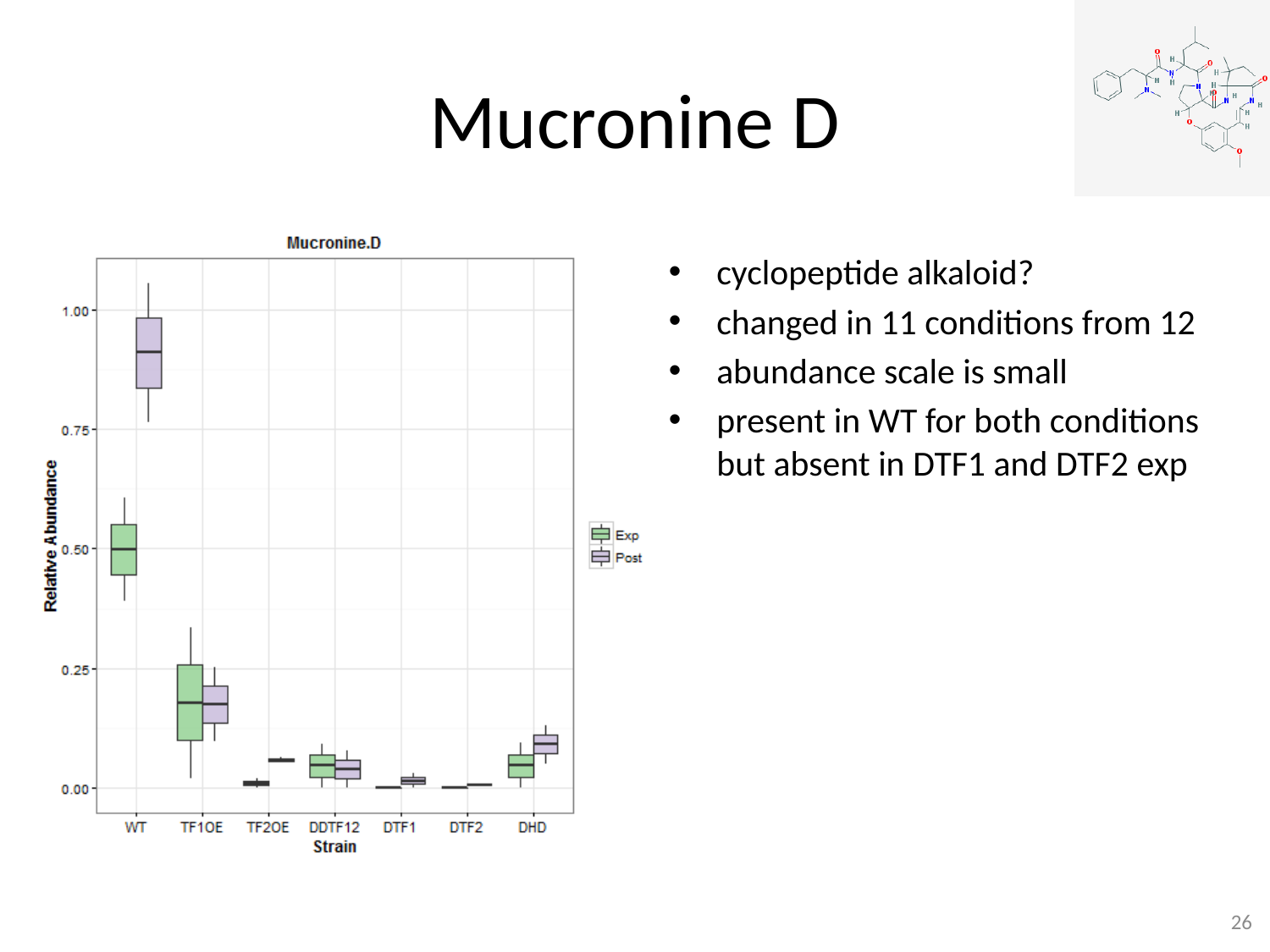

# Mucronine D
cyclopeptide alkaloid?
changed in 11 conditions from 12
abundance scale is small
present in WT for both conditions but absent in DTF1 and DTF2 exp
26

## Slide 27
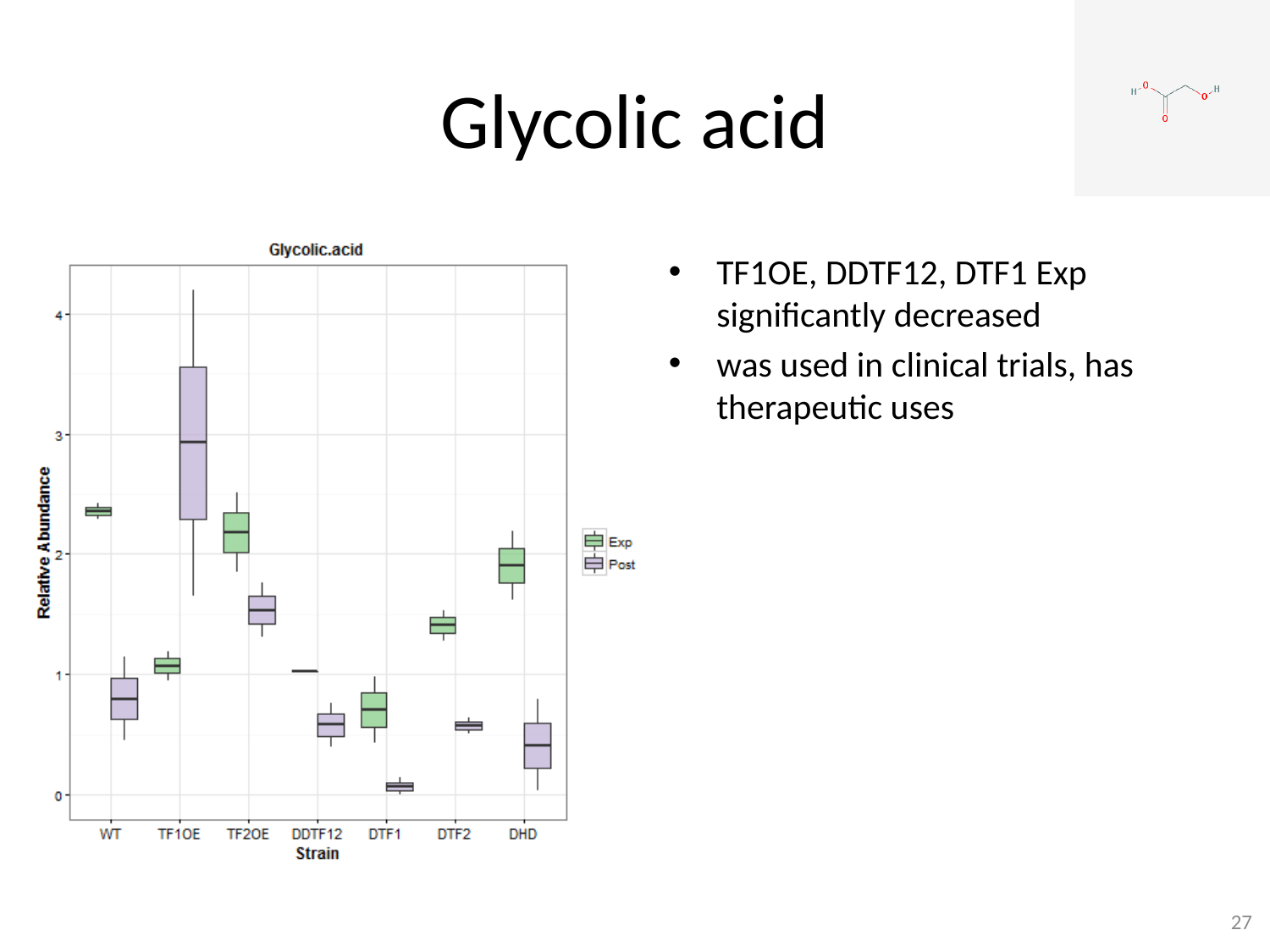

# Glycolic acid
TF1OE, DDTF12, DTF1 Exp significantly decreased
was used in clinical trials, has therapeutic uses
27

## Slide 28
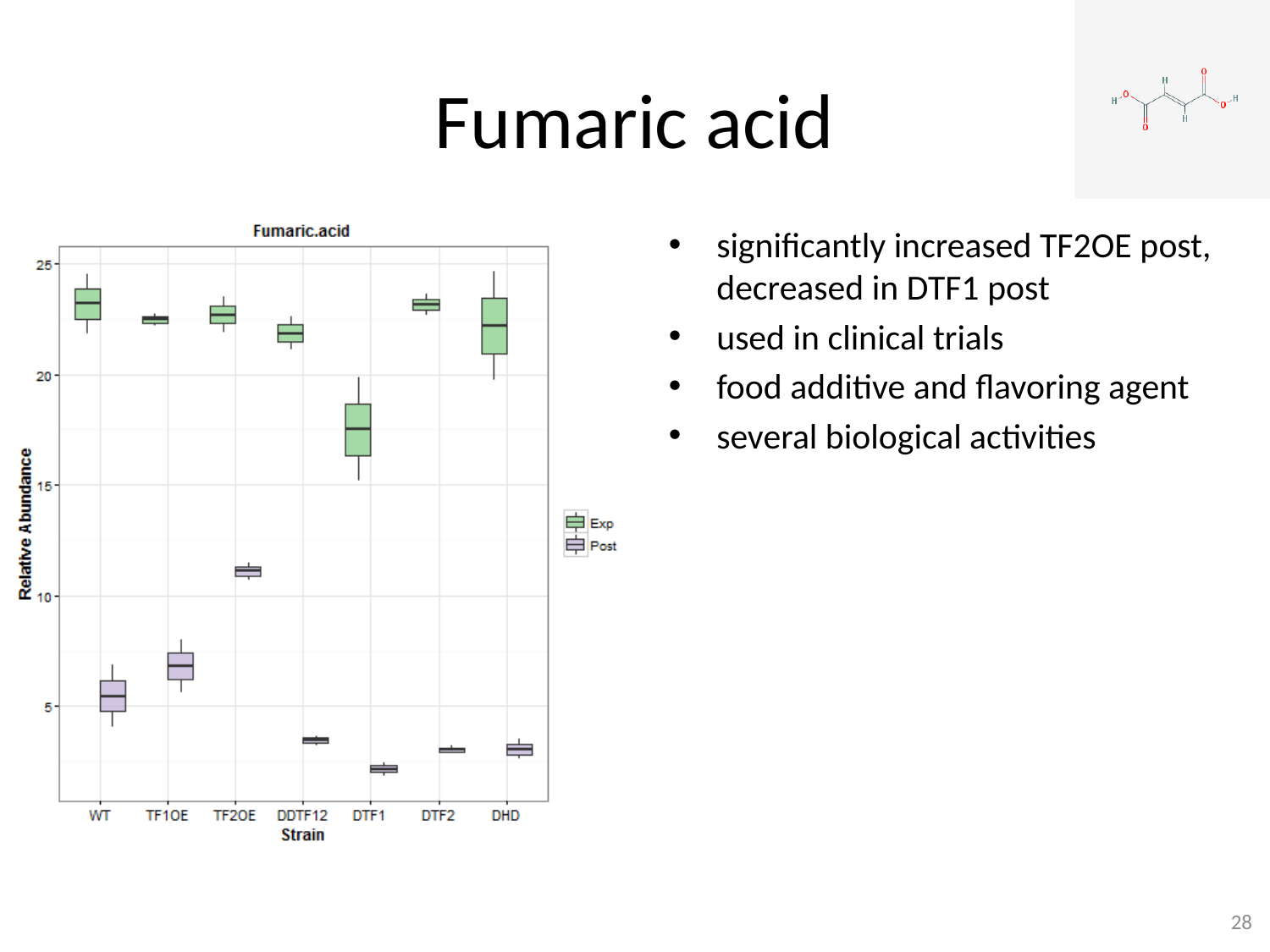

# Fumaric acid
significantly increased TF2OE post, decreased in DTF1 post
used in clinical trials
food additive and flavoring agent
several biological activities
28

## Slide 29
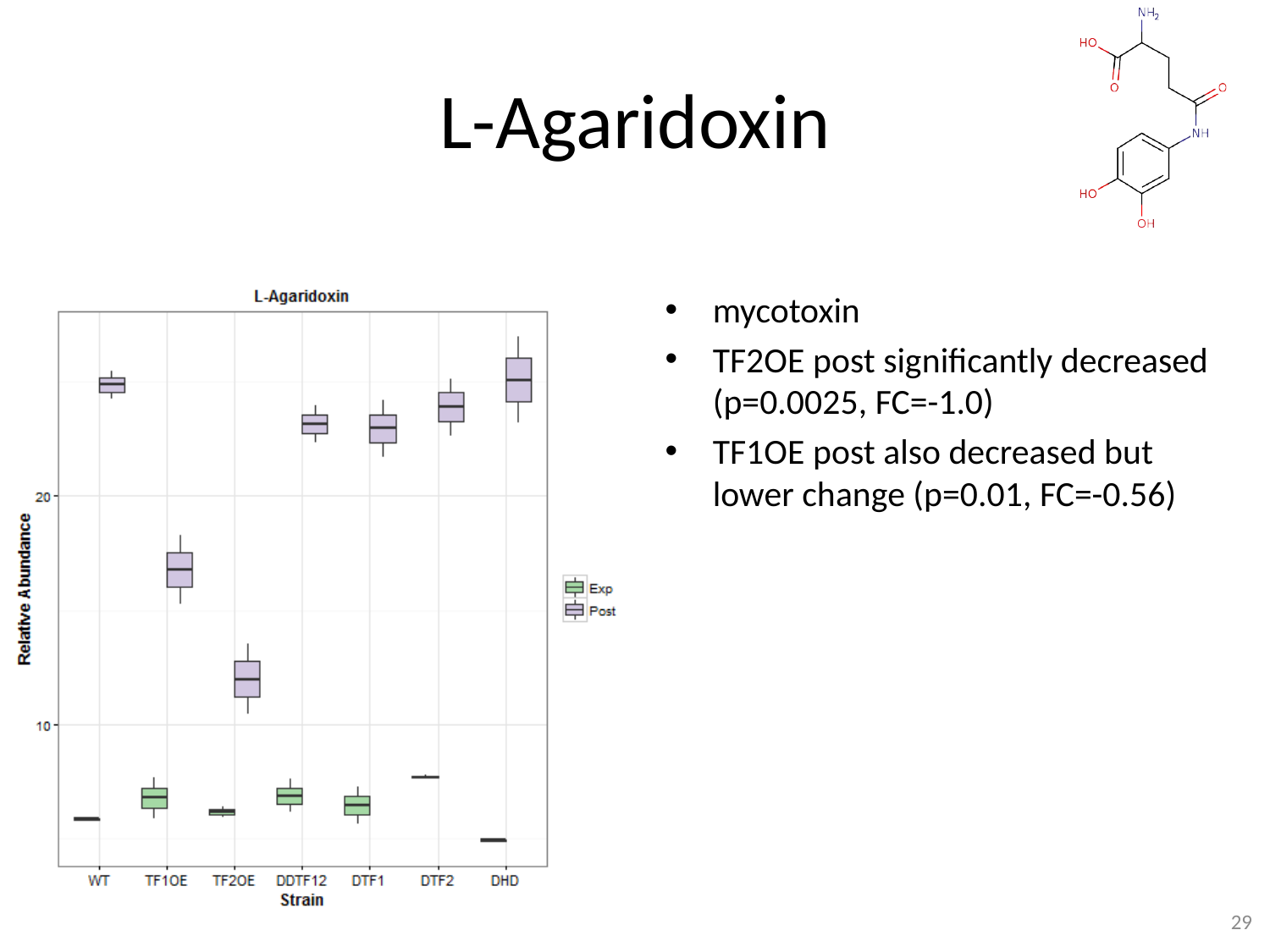

# L-Agaridoxin
mycotoxin
TF2OE post significantly decreased (p=0.0025, FC=-1.0)
TF1OE post also decreased but lower change (p=0.01, FC=-0.56)
29

## Slide 30
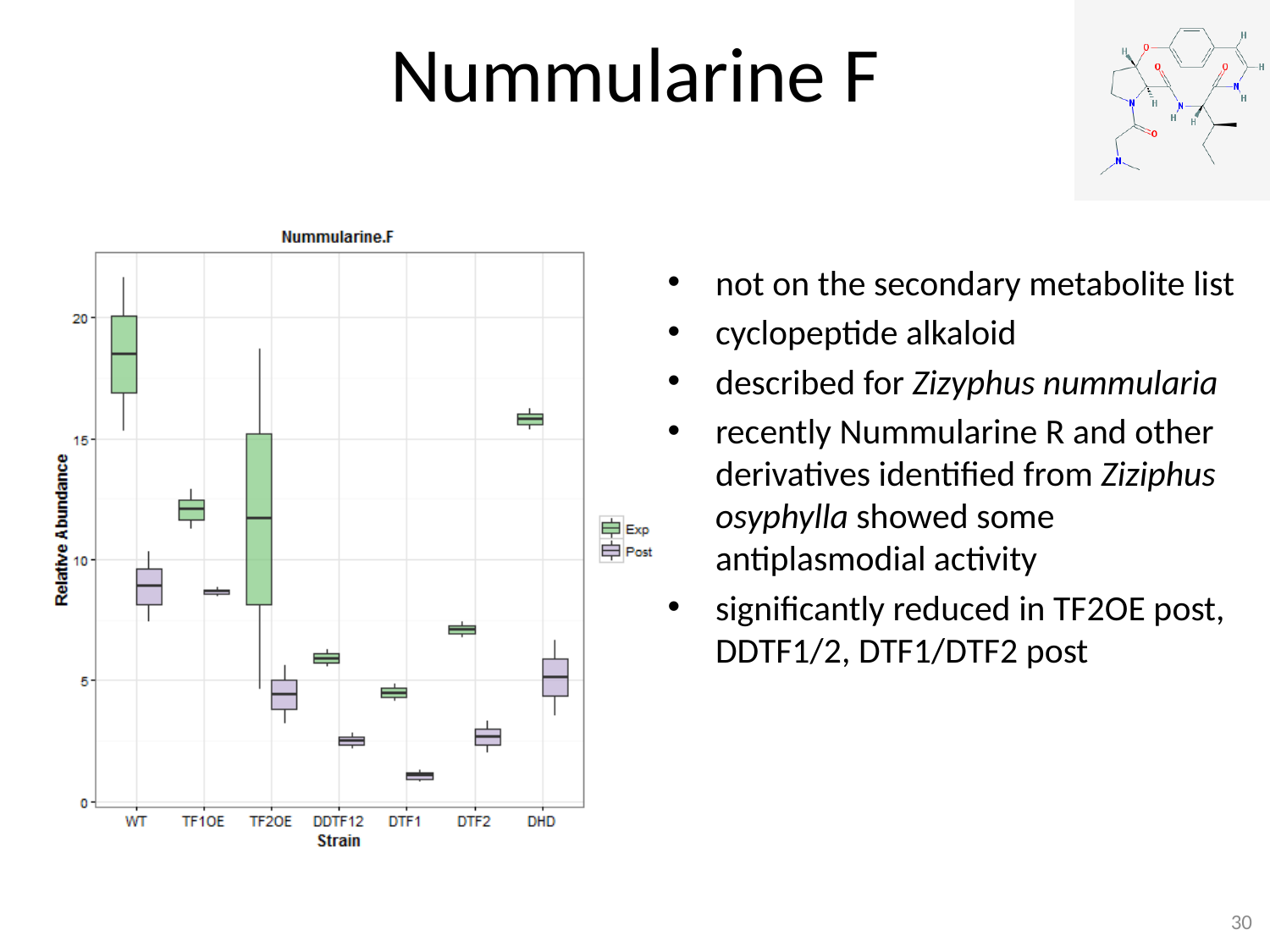

# Nummularine F
not on the secondary metabolite list
cyclopeptide alkaloid
described for Zizyphus nummularia
recently Nummularine R and other derivatives identified from Ziziphus osyphylla showed some antiplasmodial activity
significantly reduced in TF2OE post, DDTF1/2, DTF1/DTF2 post
30

## Slide 31
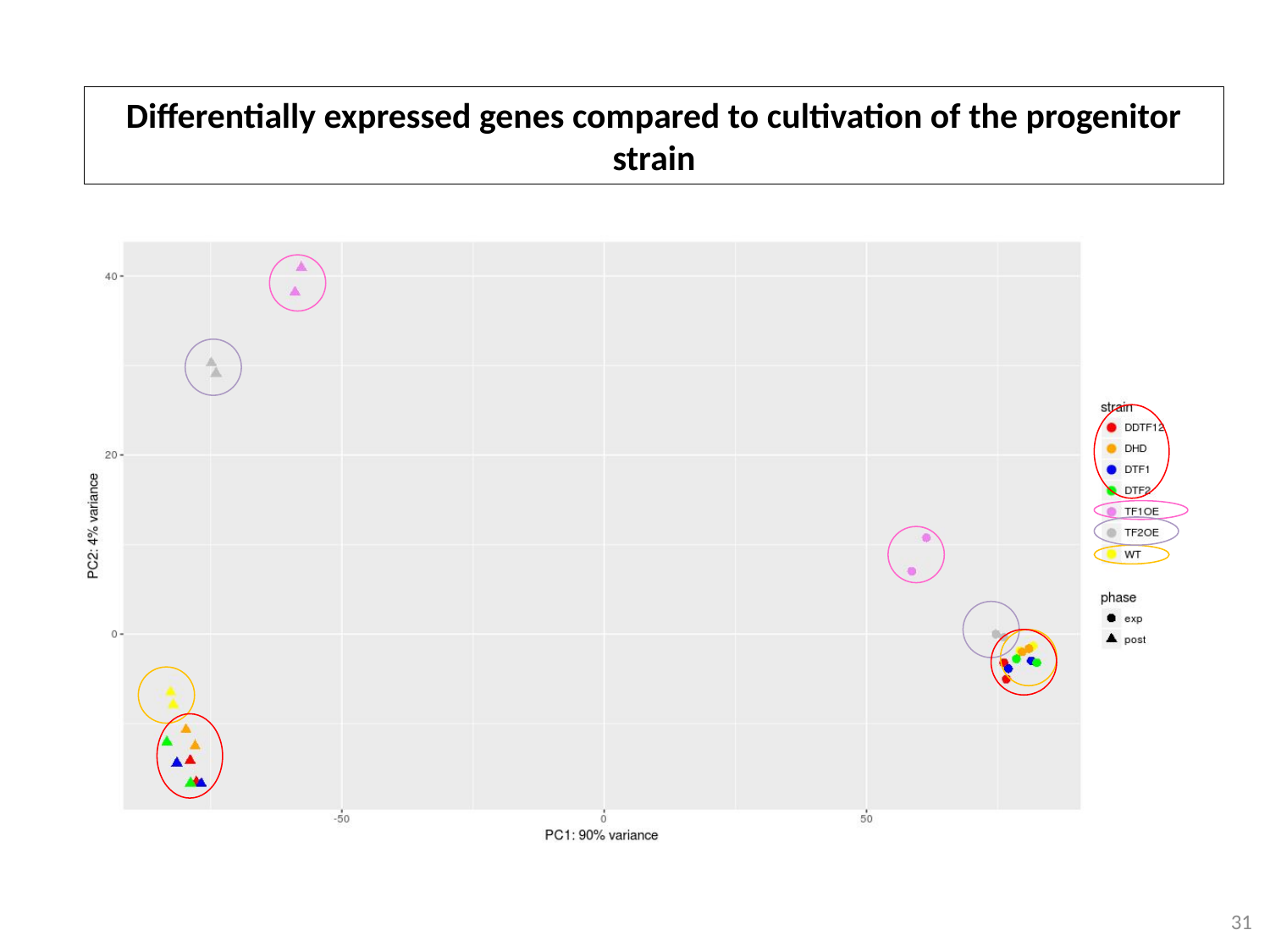

Differentially expressed genes compared to cultivation of the progenitor strain
31

## Slide 32
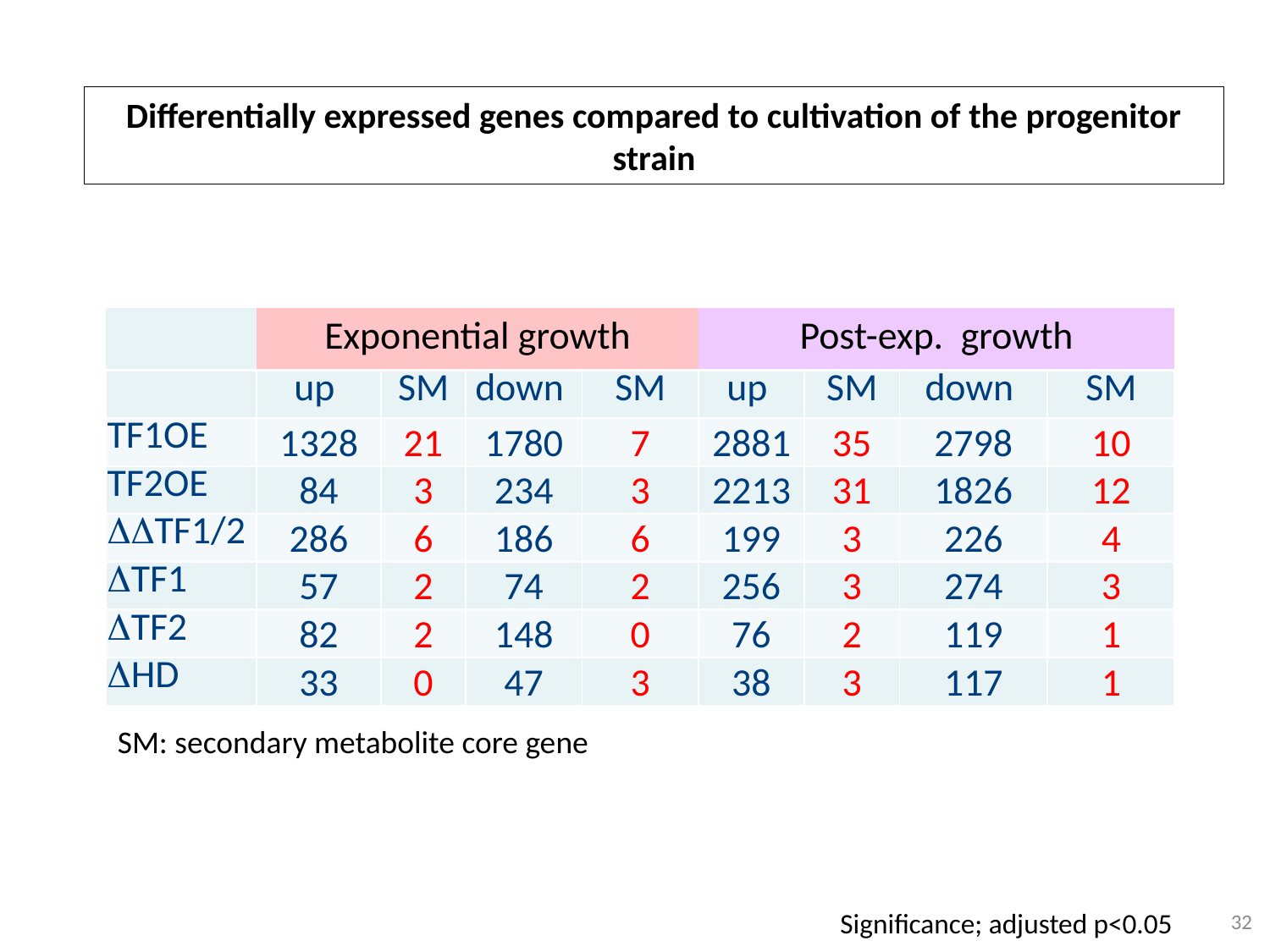

Differentially expressed genes compared to cultivation of the progenitor strain
| | | | | | | | | | |
| --- | --- | --- | --- | --- | --- | --- | --- | --- | --- |
| | Exponential growth | | | | Post-exp. growth | | | | |
| | up | SM | down | SM | up | SM | down | | SM |
| TF1OE | 1328 | 21 | 1780 | 7 | 2881 | 35 | 2798 | | 10 |
| TF2OE | 84 | 3 | 234 | 3 | 2213 | 31 | 1826 | | 12 |
| DDTF1/2 | 286 | 6 | 186 | 6 | 199 | 3 | 226 | | 4 |
| DTF1 | 57 | 2 | 74 | 2 | 256 | 3 | 274 | | 3 |
| DTF2 | 82 | 2 | 148 | 0 | 76 | 2 | 119 | | 1 |
| DHD | 33 | 0 | 47 | 3 | 38 | 3 | 117 | | 1 |
SM: secondary metabolite core gene
32
Significance; adjusted p<0.05
