# Supplementary material for: Updating genome annotation for the microbial cell factory Aspergillus niger using gene co-expression networks
Source: Nucleic Acids Res. 2018 Nov 29;47(2):559–69. doi: 10.1093/nar/gky1183 (PMC6344863; doi:10.1093/nar/gky1183)
Supplement: Supplementary Data [file gky1183_supplemental_files.zip › Suppl. Table 7_strains used_Proof edited.docx]

**Supplementary Table 7 – Strains used in this study.**

| Strain name | Relevant genotype/description | Reference |
| --- | --- | --- |
| N402 | *cspA^-^* (derivative of ATCC9029) | Bos *et al.* 1988 |
| AB4.1 | *pyrG^-^* derivative of N402 (*pyrG378*) | Van Hartingsveldt *et al.* 1987 |
| BG1.1 | Tet-on-*mluc*-T_trpC_, *AopyrG^+^*, single copy at An11g08480 locus (derivative of AB4.1) | This study |
| BG3.1 | Tet-on-*mluc*-T_trpC_, *AopyrG^+^*, single copy at An11g07580 locus (derivative of AB4.1) | This study |
| BG6.14 | Tet-on-*mluc*-T_trpC_, *AopyrG^+^*, single copy at An11g11310 locus (derivative of AB4.1) | This study |
| VG8.27 | Tet-on-*mluc*-T_trpC_, *pyrG^+^*, single copy at *pyrG* locus (An12g03570) | [30], Meyer *et al.* 2011 |
| PK2.9 | *Panafp*::*mluc, Δanafp, AopyrG^+^* (derivative of AB4.1) | [29], Paege *et al.* 2016 |
| BBA17.9 | *Panafp*::*mluc,* *Δanafp,* Δ*stuA*::*hygB* (derivative of PK2.9) | This study |
| BBA13.2 | *Panafp*::*mluc,* *Δanafp,* Δ*velC*::*hygB* (derivative of PK2.9) | This study |
| MA169.4 | Δ*kusA*::DR-*amdS*-DR, *pyrG^-^* (derivative of AB4.1) | Carvalho *et al.* 2010 |
| MF41.3 | Δ*kusA*::DR-*amdS*-DR, *pyrG^-^*, *hisB^-^* (derivative of MA169.4) | Fiedler *et al.* 2017 |
| MF42.2 | Δ*kusA*::DR-*amdS*-DR, *pyrG^+^*, *hisB^-^* (derivative of MF41.3) | Fiedler *et al.* 2017 |
| MJK10.12 | Δ*kusA*::DR-*amdS*-DR, Tet-on-*mjkA*-T_trpC_, pyrG+, multiple copy at *pyrG* locus (derivative of MA169.4) | This study |
| MJK11.17 | Δ*kusA*::DR-*amdS*-DR, Tet-on-*mjkB*-T_trpC_, pyrG+, single copy at *pyrG* locus (derivative of MA169.4) | This study |
| MJK12.11 | Δ*kusA*::DR-*amdS*-DR, Tet-on-*hdaX*-T_trpC_, pyrG+, single copy at *pyrG* locus (derivative of MA169.4) | This study |
| MJK15.4 | Δ*kusA*::DR-*amdS*-DR, *ΔmjkA*::*hisB* (derivative of MF42.2) | This study |
| MJK16.5 | Δ*kusA*::DR-*amdS*-DR, *ΔmjkB*::*hisB* (derivative of MF42.2) | This study |
| MJK14.7 | Δ*kusA*::DR-*amdS*-DR, *ΔhdaX*::*hisB* (derivative of MF42.2) | This study |
| MJK18.1 | Δ*kusA*::DR-*amdS*-DR, *ΔmjkA*::*Anidhis3,* ΔMjkB::*AopyrG* (derivative of MF41.3) | This study |
| MJK17.25 | Δ*kusA*::DR-*amdS*-DR, *pyrG^+^* (derivative of MA169.4) | This study |

Note: TF1 has been given the gene name *mjkA*, TF2 has been given the gene name *mjkB*, HD has been given the gene name *hdaX*

**References**

Bos *et al.* 1988, Curr Genet. 14:437-43.

Van Hartingsveldt *et al.* 1987, Mol Gen Genet. 206:71-5.

Carvalho *et al.* 2010, Appl Microbiol Biotechnol. 87:1463-73.

Fiedler *et al.* 2017, BMC Microbiol. 17(1):57.
