# Supplementary material for: Updating genome annotation for the microbial cell factory Aspergillus niger using gene co-expression networks
Source: Nucleic Acids Res. 2018 Nov 29;47(2):559–69. doi: 10.1093/nar/gky1183 (PMC6344863; doi:10.1093/nar/gky1183)
Supplement: Supplementary Data [file gky1183_supplemental_files.zip › Suppl._Table_2_hypothetical_ORFs_R1.docx]

**Suppl. Table 2: Assigning hypothetical genes putative biological processes based on gene co-expression network analysis.** One contig of the *A. niger* genome (Pel et al., 2007, [Nat Biotechnol.](https://www.ncbi.nlm.nih.gov/pubmed/?term=herman+pel+nature+biotechnology) 25(2):221-31) was randomly chosen, hypothetical genes extracted, and checked for how many genes functional processes based on (i) enriched gene ontology (GO) terms using Fisher’s exact (p-value >0.05), and (ii) based on co-expression with experimentally verified ORFs. Essentially, at the highest stringency of 0.7, for about 40% of hypothetical ORFs, clear biological processes can be allocated.

| **QUERY GENE** | **Description** | **Enriched GO terms from sub-network (**\|**0.7**\| **Spearman cut-off)** | **Co-expression with informant ORF** | **Spearman** | **Gene name** | **Experimentally confirmed function in *Aspergillus* spp.** |
| --- | --- | --- | --- | --- | --- | --- |
| **An04g09490** | Hypothetical Protein | GO:1902060: positive regulation of sporocarp development involved in sexual reproduction  GO:0070798: positive regulation of cleistothecium development  GO:0031155: regulation of reproductive fruiting body development | An14g02100 | 0.8 | *cwpA* | Glycosylphosphatidylinositol (GPI)-anchored cell wall mannoprotein |
|  |  |  | An04g08900 | 0.75 |  | Extracellular protein of unknown function; induced by carbon starvation-induced autophagy |
|  |  |  | An01g12550 | 0.7 | *msdS* | 1,2-alpha-mannosidase; secreted protein; fibrinogen-binding |
|  |  |  | An06g00170 | 0.7 | *aglA* | Alpha-galactosidase; alpha-N-acetylgalactosaminidase variant A; CreA regulated; extracellular |
|  |  |  | An08g01710 | 0.7 | *abfC* | Protein with alpha-arabinofuranosidase activity, involved in degradation of pectin |
| **An04g02430** | Hypothetical Protein | GO:0051278: fungal-type cell wall polysaccharide biosynthetic process  GO:0042493: response to drug  GO:0046369: galactose biosynthetic process  GO:1901356: beta-D-galactofuranose metabolic process | An12g08720 | 0.7 | *gfsA* | Galactofuranosyltransferase involved in biosynthesis of galactofuranose antigen of cell wall O-glycan |
|  |  |  | An02g08670 | 0.7 | *ugtA* | Putative UDP-galactofuranose transporter involved in import of UDP-galactofuranose into the Golgi lumen |
| **An04g03480** | Hypothetical Protein | GO:0090304: nucleic acid metabolic process  GO:0071790: establishment of spindle pole body localization to nuclear envelope  GO:0006913: nucleocytoplasmic transport  GO:0009267: cellular response to starvation | An01g14330 | 0.75 | *kapC* | Non-essential nuclear transport receptor importin-beta2 subunit; karyopherin superfamily; expression reduced after exposure to farnesol |
|  |  |  | An07g01520 | 0.75 | *nup170* | Putative nuclear pore complex protein |
|  |  |  | An11g04630 | 0.75 | *nup159* | Putative nuclear pore complex protein; localizes to the nuclear periphery during interphase and disperses throughout the cell during mitosis |
|  |  |  | An12g00690 | 0.75 | *nup85* | Putative nuclear pore complex protein with homology to Saccharomyces cerevisiae Nup85p |
|  |  |  | An07g04000 | 0.7 | *acdX* | Subunit of the SAGA transcriptional regulatory complex; mutations abolish acetate repression; WD repeat protein; locus contains the conserved upstream open reading frame (uORF) AN4670-uORF |
|  |  |  | An11g10650 | 0.7 | *nup37* | Putative nuclear pore complex protein that physically interacts with sonB |
|  |  |  | An04g01520 | 0.7 | *ndc1* | Putative transmembrane-containing nuclear pore complex protein with homology to Saccharomyces cerevisiae Ndc1p; synthetically lethal with null mutations in nup84, nup120 or nup133 |
|  |  |  | An08g00720 | 0.7 | *bop1* | Putative WD40 repeat nucleolar protein; ortholog of <I>S. cerevisiae</I> Erb1p which has role in rRNA processing and ribosomal large subunit biogenesis; expression reduced after exposure to farnesol |
| **An04g04930** | Hypothetical Protein | GO:1900196: regulation of penicillin biosynthetic process  GO:0017000: antibiotic biosynthetic process  GO:0030705: cytoskeleton-dependent intracellular transport | An09g04090 | 0.7 | *pipA* | Putative component of the EKC/KEOPS complex; interacts with PtkA, cyclin-dependent kinase, in metulae, phialides and conidia; essential; involved in hyphal growth and asexual development |
|  |  |  | An15g03350 | 0.7 | *palC* | Protein involved in a signaling pathway that activates PacC transcription factor in response to alkaline ambient pH |
| **An04g00010** | Hypothetical Protein | GO:2000397: positive regulation of ubiquitin-dependent endocytosis  GO:0070201: regulation of establishment of protein localization  GO:0006501: C-terminal protein lipidation  GO:0001907: killing by symbiont of host cells  GO:0044004: disruption by symbiont of host cell  GO:0044179: hemolysis in other organism | An04g01210 | 0.75 | *mag1* | Putative CMP glycosylase with a predicted role in nucleotide salvage pathways; plays a role in DNA damage response; multicopy suppressor of sonA1 mutation |
|  |  |  | An11g11320 | 0.75 | *atg4* | Cysteine protease required for autophagosome formation; required for conidiation |
|  |  |  | An11g01000 | 0.7 | *fos-1* | Putative histidine kinase, two-component signal transduction protein; transcript level increased significantly during infection of mice |
|  |  |  | An02g03310 | 0.7 |  | Ortholog(s) have role in intracellular sterol transport and fungal-type vacuole lumen localization |
|  |  |  | An10g00350 | 0.7 |  | Putative bifunctional GTP cyclohydrolase II; intracellular; protein abundance decreased by menadione stress |
| **An04g00290** | Hypothetical Protein | GO:1900396: positive regulation of kojic acid biosynthetic process  GO:0042181: ketone biosynthetic process  GO:0009698: phenylpropanoid metabolic process  GO:0019748: secondary metabolic process | An11g00250 | 0.75 | *pynA* | Putative polyketide synthase (PKS) - nonribosomal peptide synthase (NRPS) hybrid, encoded in a secondary metabolite gene cluster involved in production of pyranonigirin E |
|  |  |  | An01g08160 | 0.7 | *gcnE* | Subunit of the SAGA transcriptional regulatory complex with a role in nucleosome positioning |
|  |  |  | An09g05060 | 0.7 | *kojR* | Zn(II)2Cys6 transcription factor; induced by kojic acid; present in the kojic acid biosynthetic gene cluster |
|  |  |  | An08g07210 | 0.7 | *flbE* | Protein involved in regulation of conidiophore development; interacts with putative transcription factor FlbB; specific to filamentous fungi |
|  |  |  | An12g01170 | 0.7 | *sgdD* | Putative malonyl CoA synthetase; required for conidial germination |
| **An04g08570** | Hypothetical Protein | GO:0070262: peptidyl-serine dephosphorylation  GO:0019619: 3,4-dihydroxybenzoate catabolic process GO:0032075: positive regulation of nuclease activity | An12g01170 | 0.75 | *sgdD* | Putative malonyl CoA synthetase; required for conidial germination |
|  |  |  | An11g00250 | 0.7 | *pynA* | Putative polyketide synthase (PKS) - nonribosomal peptide synthase (NRPS) hybrid, encoded in a secondary metabolite gene cluster involved in production of pyranonigirin E |
|  |  |  | An11g00330 | 0.7 | *pynE* | Predicted P450-related reductase, encoded in a secondary metabolite gene cluster involved in production of pyranonigirin E |
|  |  |  | An09g02900 | 0.7 | *aipC* | Predicted receptor-mediated endocytosis and actin cortical patch localization |
| **An04g00460** | Hypothetical protein | GO:0006412: translation  GO:0043604: amide biosynthetic process  GO:0051649: establishment of localization in cell  GO:0032543: mitochondrial translation  GO:0019752: carboxylic acid metabolic process  GO:0006086: acetyl-CoA biosynthetic process from pyruvate | An18g03810 | -0.7 | *rpl16a* | Predicted ribosomal protein of the large (60S) ribosomal subunit; differentially expressed during sexual development |
|  |  |  | An17g00890 | -0.7 | *ppt* | Serine/threonine phosphatase; tetratricopeptide repeat (TPR) and catalytic domains located at the N- and C-terminal regions, respectively |
|  |  |  | An01g00100 | -0.7 | *pdhC* | Putative pyruvate dehydrogenase (lipoamide) with a predicted role in pyruvate metabolism |
|  |  |  | An12g00410 | -0.7 | *rrmA* | RNA binding protein involved in regulation of arginine catabolism; controls stability of mRNAs in response to nitrogen sources and oxidative stress |
|  |  |  | An01g06230 | -0.7 | *sgdA* | Putative eIF3b subunit of translation initiation factor 3 (eIF3); required for conidial germination |
|  |  |  | An02g12610 | -0.7 | *ranGAP* | Putative Ran GTPase activating protein (GAP) with homology to Saccharomyces cerevisiae Rna1p; endogenously tagged RanGAP is excluded from nuclei during interphase and disperses throughout the cell at mitosis |
|  |  |  | An18g03310 | -0.7 |  | Putative 40S ribosomal protein subunit; ortholog of <I>S. cerevisiae</I> Rps3p; expression reduced after exposure to farnesol |
|  |  |  | An07g01580 | -0.7 | *kapF* | Karyopherin; essential nuclear receptor |
