# Supplementary material for: Updating genome annotation for the microbial cell factory Aspergillus niger using gene co-expression networks
Source: Nucleic Acids Res. 2018 Nov 29;47(2):559–69. doi: 10.1093/nar/gky1183 (PMC6344863; doi:10.1093/nar/gky1183)
Supplement: Supplementary Data [file gky1183_supplemental_files.zip › Suppl._Table_3_regulators_R1_.docx]

**Suppl. Table 3: *A. niger* co-expression resources enable facile predictions of biological process for various regulatory genes.** The Ensembl database was interrogated for kinases, histone deacetylases, and transcription factors that lack (i) experimental characterization, (ii) informant orthologs from genomes such as *A. nidulans* or *S. cerevisiae* or (iii) predictions of biological process that add additional information to their putative molecular function. Consequently, these genes have highly limited biological processes predictions. We were able to rapidly assign predictions for all ORFs using the co-expression network and GO enrichment (Fisher’s exact p < 0.05) from either positive or negatively correlated datasets. Examples for nine ORFs are shown. The complete gene list is given in Suppl. Table 1.

|  | **Ensembl Annotation** | | | | **Co-expression annotation** | | | | |
| --- | --- | --- | --- | --- | --- | --- | --- | --- | --- |
| **Query Gene** | **Description** | **GO Biological Process** | **GO molecular Function** | **GO Cellular Component** | **GO enrichment of sub-network (**\|**0.5**\| **Spearman correlation cut-off)** | **Co-expression with informant ORFs** | **Spearman** | **Name** | **Experimentally confirmed function in either *S. cerevisiae*, *A. niger, A. nidulans, A. oryzae,* or *A. fumigatus*** |
| **An11g01250** | Has domain(s) with predicted ATP binding, protein serine/threonine kinase activity and role in protein phosphorylation | Protein phosphorylation | ATP binding, Protein serine/threonine kinase activity | Unknown | GO:0005980: glycogen catabolic process  GO:0016236: macroautophagy  GO:0016237: lysosomal microautophagy  GO:0000422: mitophagy  GO:0000045: autophagosome assembly  GO:0006508: proteolysis  GO:0007034: vacuolar transport | An07g10020 | 0.75 | *atg8* | Putative autophagy-related ubiquitin modifier; autophagosomal membrane protein; essential for autophagy |
|  |  |  |  |  |  | An02g07210 | 0.7 | *pepE* | Putative acid aspartic protease, constitutively expressed |
|  |  |  |  |  |  | An08g01060 | 0.7 | *hulA* | Putative HECT ubiquitin ligase |
|  |  |  |  |  |  | An11g11320 | 0.7 | *atg4* | Cysteine protease required for autophagosome formation; required for conidiation |
|  |  |  |  |  |  | An04g08900 | 0.75 |  | Protein of unknown function; induced by carbon starvation-induced autophagy |
|  |  |  |  |  |  | An04g05300 | 0.7 | *acuG* | Putative fructose-bisphosphatase with a predicted role in gluconeogenesis and glycolysis; intracellular; protein abundance decreased by menadione stress; expression upregulated after exposure to farnesol |
| **An09g06520** | Ortholog(s) have role in chromatin silencing at telomere, histone deacetylation | Chromatin silencing at telomere, Histone deacetylation | NAD+ binding | Unknown | GO:0019748: secondary metabolic process  GO:0044550: secondary metabolite biosynthetic process  GO:0070807: positive regulation of phialide development  GO:1902181: verruculogen biosynthetic process  GO:0033246: positive regulation of penicillin metabolic process  GO:0070828: heterochromatin organization | An12g02670 | -0.55 |  | Putative polyketide synthase (PKS), encoded in a predicted secondary metabolite gene cluster |
|  |  |  |  |  |  | An09g05110 | -0.5 |  | Protein similar to nonribosomal peptide synthases (NRPS-like), encoded in a predicted secondary metabolite gene cluster (1) |
|  |  |  |  |  |  | An06g01300 | -0.5 | *sidD* | sidC, nonribosomal peptide synthase (NRPS), encoded in a secondary metabolite cluseter |
|  |  |  |  |  |  | An11g00050 | -0.5 |  | Putative nonribosomal peptide synthase (NRPS), encoded in a predicted secondary metabolite gene cluster (1) |
| **An04g06640** | Has domain(s) with predicted DNA binding transcription factor activity, sequence-specific DNA binding activity and role in regulation of transcription, DNA-templated | Regulation of transcription, DNA-templated | DNA binding transcription factor activity | Unknown | GO:0043934: sporulation  GO:0030154: cell differentiation  GO:0003006: developmental process involved in reproduction  GO:0030584: sporocarp development  GO:0070787: conidiophore development  GO:0030448: hyphal growth  GO:0006030: chitin metabolic process  GO:0015918: sterol transport  GO:0005978: glycogen biosynthetic process  GO:0061794: conidium development  GO:0044036: cell wall macromolecule metabolic process  GO:0034221: fungal-type cell wall chitin biosynthetic process | An05g00480 | 0.85 | *stuA* | Putative APSES domain transcription factor; regulated by phoB; expression positively correlates with developmental competence in submerged culture; mutants are delayed in developmental competence |
|  |  |  |  |  |  | An09g02290 | 0.75 | *chsD* | Class IV chitin synthase involved in chitin biosynthesis |
|  |  |  |  |  |  | An18g02510 | 0.75 | *bud3* | Putative Rho guanyl nucleotide exchange factor (Rho-GEF); required for septum formation |
|  |  |  |  |  |  | An02g06660 | 0.75 | *midA* | Predicted adhesin-like protein; localized to the cell surface; required for elevated temperature tolerance and resistance to Congo Red and calcofluor white |
|  |  |  |  |  |  | An18g03040 | 0.7 | *ppsA* | Putative dual-specificity protein tyrosine/serine/threonine phosphatase |
|  |  |  |  |  |  | An03g04690 | 0.7 | *sho1* | Putative transmembrane osmosensor with homology to S. cerevisiae Sho1p; required for normal hyphal growth, condial germination, actin distribution and the response to oxidative stress; not required for invasive pulmonary infection |
|  |  |  |  |  |  | An12g00710 | 0.7 | *esdC* | Protein with a glycogen binding domain involved in sexual development; regulated by VeA and FlbA |
| **An04g03440** |  |  |  |  | GO:0030436: asexual sporulation  GO:0019953: sexual reproduction  GO:0043935: sexual sporulation resulting in formation of a cellular spore  GO:0003006: developmental process involved in reproduction  GO:0075259: spore-bearing organ development  GO:0000909: sporocarp development involved in sexual reproduction  GO:0006037: cell wall chitin metabolic process  GO:0043937: regulation of sporulation  GO:0045595: regulation of cell differentiation  GO:0006031: chitin biosynthetic process  GO:0030448: hyphal growth  GO:0010383: cell wall polysaccharide metabolic process | An18g03040 | 0.7 | *ppsA* | Putative dual-specificity protein tyrosine/serine/threonine phosphatase |
|  |  |  |  |  |  | An18g02510 | 0.7 | *bud3* | Putative Rho guanyl nucleotide exchange factor (Rho-GEF); required for septum formation |
|  |  |  |  |  |  | An05g00480 | 0.7 | *stuA* | Putative APSES domain transcription factor; regulated by phoB; expression positively correlates with developmental competence in submerged culture; mutants are delayed in developmental competence |
|  |  |  |  |  |  | An02g13750 | 0.7 | *gtaA* | Putative glutaminase A with a predicted role in glutamate and glutamine metabolism |
| **An01g07140** |  |  |  |  | GO:0006073: cellular glucan metabolic process  GO:0005982: starch metabolic process  GO:0043410: positive regulation of MAPK cascade  GO:0043207: response to external biotic stimulus  GO:0000199: activation of MAPK activity involved in cell wall organization or biogenesis  GO:0016051: carbohydrate biosynthetic process  GO:0070590: spore wall biogenesis  GO:0033692: cellular polysaccharide biosynthetic process | An04g06910 | 0.7 | *amyR* | Zn(II)2 Cys6 transcriptional activator involved in starch metabolism; responsible for induction of amylolytic genes |
|  |  |  |  |  |  | An03g06550 | 0.7 | *glaA* | Secreted glucoamylase required for starch metabolism |
| **An18g01770** |  |  |  |  | GO:0042274: ribosomal small subunit biogenesis  GO:0042273: ribosomal large subunit biogenesis  GO:0033750: ribosome localization  GO:0000054: ribosomal subunit export from nucleus  GO:0000460: maturation of 5.8S rRNA | An08g00720 | 0.7 | *bop1* | Putative WD40 repeat nucleolar protein; ortholog of <I>S. cerevisiae</I> Erb1p which has role in rRNA processing and ribosomal large subunit biogenesis; expression reduced after exposure to farnesol |
|  |  |  |  |  |  | An01g00070 | 0.7 | *trm1* | Putative 2,N2-dimethylguanosine tRNA methyltransferase; mutants are self-sterile |
|  |  |  |  |  |  | An16g08030 | 0.7 | *rpf2* | Ribosome assembly factor; component of the Rpf2-Rrs1 complex; ortholog of <i>S. cerevisiae</i> Rpf2p; expression reduced after exposure to farnesol |
| **An01g14020** |  |  |  |  | GO:0072666: establishment of protein localization to vacuole  GO:0006914: autophagy  GO:0044805: late nucleophagy  GO:1903008: organelle disassembly  GO:0016236: macroautophagy  GO:0006508: proteolysis  GO:0019954: asexual reproduction  GO:0016237: lysosomal microautophagy  GO:0007033: vacuole organization  GO:0006635: fatty acid beta-oxidation  GO:0005980: glycogen catabolic process  GO:0012501: programmed cell death | An07g10020 | 0.75 | *atg8* | Putative autophagy-related ubiquitin modifier; autophagosomal membrane protein; essential for autophagy |
|  |  |  |  |  |  | An11g11320 | 0.75 | *atg4* | Cysteine protease required for autophagosome formation; required for conidiation |
|  |  |  |  |  |  | An09g04470 | 0.7 | *casB* | Predicted metacaspase, an aspartate-specific cysteine protease involved in apoptosis; mutants show growth defects under conditions of ER stress |
| **An07g07370** |  |  |  |  | GO:0016999: antibiotic metabolic process  GO:0030436: asexual sporulation  GO:0017000: antibiotic biosynthetic process  GO:0042318: penicillin biosynthetic process  GO:0072339: cellular lactam biosynthetic process  GO:0000909: sporocarp development involved in sexual reproduction | An11g09720 | 0.75 |  | Putative polyketide synthase (PKS), encoded in a predicted secondary metabolite gene cluster (1) |
|  |  |  |  |  |  | An02g00840 | 0.6 |  | Protein similar to nonribosomal peptide synthases (NRPS-like), encoded in a predicted secondary metabolite gene cluster (1) |
|  |  |  |  |  |  | An04g04340 | 0.6 |  | Putative polyketide synthase (PKS), encoded in a predicted secondary metabolite gene cluster (1) |
|  |  |  |  |  |  | An15g02130 | 0.6 |  | Putative polyketide synthase (PKS), encoded in a predicted secondary metabolite gene cluster (1) |
|  |  |  |  |  |  | An01g06930 | 0.6 |  | Putative polyketide synthase (PKS), encoded in a predicted secondary metabolite gene cluster (1) |
|  |  |  |  |  |  | An15g07530 | 0.55 |  | Putative nonribosomal peptide synthase (NRPS), encoded in a predicted secondary metabolite gene cluster (1) |
|  |  |  |  |  |  | An08g04820 | -0.5 |  | Protein similar to nonribosomal peptide synthases (NRPS-like), encoded in a predicted secondary metabolite gene cluster (1) |
|  |  |  |  |  |  | An11g00250 | -0.5 | *pynA* | Putative polyketide synthase (PKS) - nonribosomal peptide synthase (NRPS) hybrid, encoded in a secondary metabolite gene cluster involved in production of pyranonigirin E (1, 2) |
|  |  |  |  |  |  | An03g03520 | -0.55 | *sidD* | Putative nonribosomal peptide synthase (NRPS), encoded in a predicted secondary metabolite gene cluster (2) |
|  |  |  |  |  |  | An13g01840 | -0.65 |  | Putative dimethylallyl tryptophan synthase (DMAT), encoded in a predicted secondary metabolite gene cluster (1) |
| **An12g07690** |  |  |  |  | GO:0072338: cellular lactam metabolic process  GO:0042316: penicillin metabolic process  GO:0030435: sporulation resulting in formation of a cellular spore  GO:0030436: asexual sporulation  GO:0017000: antibiotic biosynthetic process  GO:0042318: penicillin biosynthetic process  GO:0070787: conidiophore development | An11g09720 | 0.7 |  | Putative polyketide synthase (PKS), encoded in a predicted secondary metabolite gene cluster (1) |
|  |  |  |  |  |  | An02g00840 | 0.6 |  | Protein similar to nonribosomal peptide synthases (NRPS-like), encoded in a predicted secondary metabolite gene cluster (1) |
|  |  |  |  |  |  | An15g07530 | 0.55 |  | Putative nonribosomal peptide synthase (NRPS), encoded in a predicted secondary metabolite gene cluster (1) |
|  |  |  |  |  |  | An15g02130 | 0.55 |  | Putative polyketide synthase (PKS), encoded in a predicted secondary metabolite gene cluster (1) |
|  |  |  |  |  |  | An04g04340 | 0.5 |  | Putative polyketide synthase (PKS), encoded in a predicted secondary metabolite gene cluster (1) |
|  |  |  |  |  |  | An01g06930 | 0.5 |  | Putative polyketide synthase (PKS), encoded in a predicted secondary metabolite gene cluster (1) |
|  |  |  |  |  |  | An08g04820 | -0.5 |  | Protein similar to nonribosomal peptide synthases (NRPS-like), encoded in a predicted secondary metabolite gene cluster (1) |
|  |  |  |  |  |  | An13g01840 | -0.5 |  | Putative dimethylallyl tryptophan synthase (DMAT), encoded in a predicted secondary metabolite gene cluster (1) |
