# Supplementary material for: Updating genome annotation for the microbial cell factory Aspergillus niger using gene co-expression networks
Source: Nucleic Acids Res. 2018 Nov 29;47(2):559–69. doi: 10.1093/nar/gky1183 (PMC6344863; doi:10.1093/nar/gky1183)
Supplement: Supplementary Data [file gky1183_supplemental_files.zip › NAR-02626-2018_GEO_numbers.doc]

GEO Information Meyer et al., 2018 NAR-02626-2018

| **ID** | **ID** | **GEO** | **Expression** | **SD Expression** | **Publication / Remark** |
| --- | --- | --- | --- | --- | --- |
| **condition** | **chip** | **series*** | **level, AU** | **level** |  |
|  |  |  |  |  |  |
| 1 | 1 | GSE42258 | 53,7 | 17,9 |  |
|  | 2 | GSE42258 |  |  |  |
|  | 3 | GSE42258 |  |  |  |
| 2 | 4 | GSE42258 | 611,0 | 118,5 |  |
|  | 5 | GSE42258 |  |  |  |
| 3 | 6 | GSE42258 | 386,5 | 5,7 | Kwon MJ, et al. The transcriptomic signature of RacA activation and |
|  | 7 | GSE42258 |  |  |  |
|  |  |  |  |  | inactivation provides new insights into the morphogenetic network of |
| 4 | 8 | GSE42258 | 481,5 | 47,1 |  |
|  |  |  |  |  | Aspergillus niger. PLoS One 2013;8(7):e68946. |
|  | 9 | GSE42258 |  |  |  |
|  |  |  |  |  |  |
| 5 | 10 | GSE42258 | 1219,8 | 786,8 |  |
|  | 11 | GSE42258 |  |  |  |
| 6 | 12 | GSE42258 | 29,2 | 0,5 |  |

13 GSE42258
14 GSE42258

| 7 | 15 |  | 42,6 | 18,9 |  |
| --- | --- | --- | --- | --- | --- |
|  | 16 |  |  |  |  |
| 8 | 17 |  | 29,9 | 5,9 | Additional own unpublished data to Kwon MJ, et al. 2013 |
|  | 18 |  |  |  |  |
|  |  |  |  |  |  |
| 9 | 19 |  | 30,1 | 0,3 |  |
|  | 20 |  |  |  |  |
| 10 | 21 | GSE17641 | 26,2 | 5,5 |  |
|  | 22 | GSE17641 |  |  | Meyer V, et al. Reconstruction of signaling networks regulating fungal |
|  | 23 | GSE17641 |  |  |  |
| 11 | 24 | GSE17641 | 27,0 | 0,9 | morphogenesis by transcriptomics. Eukaryot Cell 2009 Nov;8(11):1677-91. |

25 GSE17641
26 GSE17641

| 12 | 27 | GSE56471 | 27,0 | | 3,8 |  | |
| --- | --- | --- | --- | --- | --- | --- | --- |
|  | 28 | GSE56471 |  | |  |  | |
| 13 | 29 | GSE56471 | 26,7 | | 3,6 |  | |
|  | 30 | GSE56471 |  | |  |  | |
| 14 | 31 | GSE56471 | 25,1 | | 0,7 | Fiedler M, et al. The capacity of Aspergillus niger to sense and respond to | |
|  | 32 | GSE56471 |  | |  |  |  |
|  |  |  |  | |  | cell wall stress requires at least three transcription factors: RlmA, MsnA | |
| 15 | 33 | GSE56471 | 27,2 | | 3,7 |  |  |
|  |  |  |  |  |  | AND CrzA. Fungal Biol Biotechnol 2014, 1(5). | |
|  | 34 | GSE56471 |  | |  |  |  |
|  |  |  |  | |  |  | |
| 16 | 35 | GSE56471 | 26,8 | | 0,3 |  | |
|  | 36 | GSE56471 |  | |  |  | |
| 17 | 37 | GSE56471 | 24,3 | | 1,6 |  | |
|  | 38 | GSE56471 |  | |  |  | |
| 18 | 39 | GSE56471 | 26,4 | | 2,2 |  | |
|  | 40 | GSE56471 |  | |  |  | |
| 19 | 41 | GSE56471 | 26,2 | | 9,7 |  | |
|  | 42 | GSE56471 |  | |  |  | |
| 20 | 43 |  | 26,7 | | 1,1 | Additional own unpublished data to Fiedler M, et al. 2014; testing of AFP and | |
|  | 44 |  |  | |  |  |  |
| 21 | 45 |  | 31,7 | | 5,5 | caspofungin | |
|  | 46 |  |  | |  |  | |
| 22 | 47 |  | 257,2 | | 96,3 |  | |
|  | 48 |  |  | |  |  | |
|  | 49 |  |  | |  |  | |
| 23 | 50 |  | 209,8 | | 80,2 |  | |
|  | 51 |  |  | |  | Jørgensen TR, et al. Transcriptomic comparison of Aspergillus niger growing | |
|  | 52 |  |  | |  |  |  |
|  |  |  |  | |  | on two different sugars reveals coordinated regulation of the secretory | |
| 24 | 53 |  | 194,3 | | 56,9 |  |  |
|  |  |  |  |  |  | pathway. BMC Genomics 2009, 10:44. | |
|  | 54 |  |  | |  |  |  |
|  |  |  |  | |  |  | |
|  | 55 |  |  | |  |  | |
| 25 | 56 |  | 138,4 | | 18,4 |  | |
|  | 57 |  |  | |  |  | |
|  | 58 |  |  | |  |  | |
| 26 | 59 |  | 24,4 | | 1,4 |  | |
|  | 60 |  |  | |  |  | |
| 27 | 61 |  | 25,9 | | 0,6 |  | |
|  | 62 |  |  | |  |  | |
| 28 | 63 |  | 24,4 | | 1,4 |  | |
|  | 64 |  |  | |  | Yuan XL, et al. Identification of InuR, a new Zn(II)2Cys6 transcriptional | |
| 29 | 65 |  | 28,2 | | 3,8 |  |  |
|  |  |  |  |  |  | activator involved in the regulation of inulinolytic genes in Aspergillus niger. | |
|  | 66 |  |  | |  |  |  |
|  |  |  |  | |  | Mol Genet Genomics. 2008 Jan;279(1):11-26. | |
| 30 | 67 |  | 30,0 | | 5,1 |  |  |
|  |  |  |  |  |  |  | |
|  | 68 |  |  | |  |  | |
| 31 | 69 |  | 27,1 | | 2,0 |  | |
|  | 70 |  |  | |  |  | |
| 32 | 71 |  | 50,9 | | 26,1 |  | |
|  | 72 |  |  | |  |  | |
| 33 | 73 |  | 29,5 | | 0,7 | Guillemette T, et al. Genomic analysis of the secretion stress response in | |
|  | 74 | | | the enzyme-producing cell factory Aspergillus niger BMC Genomics 2007 | | |  |
|  |  | | |  |  |  |  |

| 34 | 75 |  | 31,1 | 1,7 | . | , |
| --- | --- | --- | --- | --- | --- | --- |
|  |  |  |  |  | 8:158. |  |
|  | 76 |  |  |  |  |  |
|  |  |  |  |  |  |  |
| 35 | 77 | GSE39559 | 4013,1 | 291,0 |  |  |
|  | 78 | GSE39559 |  |  | Nitsche BM, et al. The carbon starvation response of Aspergillus niger |  |
| 36 | 79 | GSE39559 | 2854,1 | 195,4 |  |  |
|  |  |  |  |  | during submerged cultivation: insights from the transcriptome and |  |
|  | 80 | GSE39559 |  |  |  |  |
|  |  |  |  |  | secretome. BMC Genomics 2012 Aug 8;13:380. |  |
| 37 | 81 | GSE39559 | 912,4 | 89,2 |  |  |
|  |  |  |  |  |  |  |
|  | 82 | GSE39559 |  |  |  |  |
| 38 | 83 |  | 30,1 | 2,6 |  |  |
|  | 84 |  |  |  |  |  |
| 39 | 85 |  | 16956,5 | 762,1 |  |  |
|  | 86 |  |  |  |  |  |
| 40 | 87 |  | 21320,8 | 152,2 |  |  |
|  | 88 |  |  |  | van Münster JM, et al. Systems approaches to predict the functions of |  |
| 41 | 89 |  | 20195,6 | 1007,9 |  |  |
|  | 90 |  |  |  | glycoside hydrolases during the life cycle of Aspergillus niger using |  |
| 42 | 91 |  | 752,4 | 15,4 | developmental mutants ∆brlA and ∆flbA. PLoS One. 2015 Jan |  |
|  | 92 |  |  |  | 28;10(1):e0116269. |  |
| 43 | 93 |  | 660,5 | 171,5 |  |  |
|  | 94 |  |  |  |  |  |
| 44 | 95 |  | 5601,0 | 393,2 |  |  |
|  | 96 |  |  |  |  |  |
| 45 | 97 |  | 2571,6 | 443,1 |  |  |
|  | 98 |  |  |  |  |  |
| 46 | 99 | GSE21752 | 15300,4 | 1362,4 |  |  |
|  | 100 | GSE21752 |  |  | Jørgensen TR, et al. Transcriptomic insights into the physiology of |  |
|  | 101 | GSE21752 |  |  |  |  |
|  |  |  |  |  | Aspergillus niger approaching a specific growth rate of zero. Appl Environ | |
| 47 | 102 | GSE21752 | 16471,3 | 2222,5 |  |  |
|  |  |  |  |  | Microbiol 2010 Aug;76(16):5344-55. |  |
|  | 103 | GSE21752 |  |  |  |  |
|  |  |  |  |  |  |  |
|  | 104 | GSE21752 |  |  |  |  |
| 48 | 105 |  | 28,2 | 0,9 |  |  |
|  | 106 |  |  |  |  |  |
|  | 107 |  |  |  |  |  |
| 49 | 108 |  | 28,3 | 2,0 |  |  |
|  | 109 |  |  |  |  |  |
|  | 110 |  |  |  | Own unpublished data; Secretion stress response to tunicamycin |  |
| 50 | 111 |  | 24,5 | 2,1 |  |  |
|  |  |  |  |  |  |  |
|  | 112 |  |  |  |  |  |
|  | 113 |  |  |  |  |  |
| 51 | 114 |  | 22,8 | 5,0 |  |  |
|  | 115 |  |  |  |  |  |
|  | 116 |  |  |  |  |  |
| 52 | 117 | GSE39070 | 35,6 | 6,8 |  |  |

118 GSE39070
119 GSE39070

| 53 | 120 | GSE39070 | 28,5 | 0,3 | Carvalho ND, et al. Genome-wide expression analysis upon constitutive |
| --- | --- | --- | --- | --- | --- |
|  | 121 | GSE39070 |  |  |  |
|  | 122 | GSE39070 |  |  | activation of the HacA bZIP transcription factor in Aspergillus niger reveals a |
| 54 | 123 | GSE39070 | 27,1 | 1,0 | coordinated cellular response to counteract ER stress. BMC Genomics 2012 |
|  | 124 | GSE39070 |  |  | Jul 30;13:350. |
|  | 125 | GSE39070 |  |  |  |
| 55 | 126 | GSE39070 | 26,6 | 0,5 |  |

127 GSE39070
128 GSE39070

| 56 | 129 | GSE14285 | 31,7 |  |
| --- | --- | --- | --- | --- |
| 57 | 130 | GSE14285 | 31,0 |  |
| 58 | 131 | GSE14285 | 29,7 |  |
| 59 | 132 | GSE14285 | 27,0 |  |
| 60 | 133 | GSE14285 | 38,2 |  |
| 61 | 134 | GSE14285 | 38,0 |  |
| 62 | 135 | GSE14285 | 38,8 | van den Berg RA, et al. Identification of modules in Aspergillus niger by |
| 63 | 136 | GSE14285 | 50,4 |  |
|  |  |  |  | gene co-expression network analysis. Fungal Genet Biol 2010 Jun;47(6):539 |
| 64 | 137 | GSE14285 | 34,9 |  |
|  |  |  |  | 50. |
| 65 | 138 | GSE14285 | 33,3 |  |
|  |  |  |  |  |
| 66 | 139 | GSE14285 | 32,4 |  |
| 67 | 140 | GSE14285 | 31,6 |  |
| 68 | 141 | GSE14285 | 36,3 |  |
| 69 | 142 | GSE14285 | 33,1 |  |
| 70 | 143 | GSE14285 | 29,5 |  |
| 71 | 144 | GSE14285 | 29,4 |  |
| 72 | 145 | GSE11405 | 30,8 | 1,5 |
|  | 146 | GSE11405 |  |  |
|  |  |  |  | van der Veen D, et al. Analysis of variance components reveals the |
|  | 147 | GSE11405 |  | contribution of sample processing to transcript variation. Appl Environ |
|  |  |  |  | Microbiol 2009 Apr;75(8):2414-22. |

148 GSE11405
149 GSE11405

| 73 | 150 | GSE11405 | 31,8 | 0,9 |
| --- | --- | --- | --- | --- |
|  | 151 | GSE11405 |  |  |

| 74 | 152 |  | 30,8 |  |  |
| --- | --- | --- | --- | --- | --- |
| 75 | 153 |  | 36,7 |  |  |
| 76 | 154 |  | 302,9 |  |  |
| 77 | 155 |  | 3904,7 |  |  |
| 78 | 156 |  | 144,7 |  |  |
| 79 | 157 |  | 48,3 |  |  |
| 80 | 158 |  | 29,2 |  |  |
| 81 | 159 |  | 62,8 |  |  |
| 82 | 160 |  | 79,7 |  |  |
| 83 | 161 |  | 28,0 |  |  |
| 84 | 162 |  | 30,5 |  |  |
| 85 | 163 |  | 28,6 |  |  |
| 86 | 164 |  | 32,8 |  | Martens-Uzunova ES, Schaap PJ. An evolutionary conserved d-galacturonic |
| 87 | 165 |  | 29,2 |  |  |
| 88 | 166 |  | 34,1 |  | acid metabolic pathway operates across filamentous fungi capable of pectin |
| 89 | 167 |  | 42,4 |  | degradation. Fungal Genet Biol 2008, 45(11):1449-1457. |
| 90 | 168 |  | 3217,3 |  |  |
| 91 | 169 |  | 61,2 |  |  |
| 92 | 170 |  | 202,1 |  |  |
| 93 | 171 |  | 164,5 |  |  |
| 94 | 172 |  | 3518,0 |  |  |
| 95 | 173 |  | 28,4 |  |  |
| 96 | 174 |  | 25,9 |  |  |
| 97 | 175 |  | 27,5 |  |  |
| 98 | 176 |  | 531,0 |  |  |
| 99 | 177 |  | 30,2 |  |  |
| 100 | 178 |  | 32,0 |  |  |
| 101 | 179 |  | 27,3 |  |  |
| 102 | 180 |  | 5963,0 |  |  |
| 103 | 181 | GSE17329 | 87,4 |  |  |
| 104 | 182 | GSE17329 | 27,4 |  |  |
| 105 | 183 | GSE17329 | 6391,4 |  |  |
| 106 | 184 | GSE17329 | 120,5 |  |  |
| 107 | 185 | GSE17329 | 7930,3 |  |  |
| 108 | 186 | GSE17329 | 25,1 |  |  |
| 109 | 187 | GSE17329 | 97,1 |  |  |
| 110 | 188 | GSE17329 | 6077,7 |  |  |
| 111 | 189 | GSE17329 | 7610,8 |  | van den Berg RA, et al. Identification of modules in Aspergillus niger by |
| 112 | 190 | GSE17329 | 4735,2 |  |  |
|  |  |  |  |  | gene co-expression network analysis. Fungal Genet Biol 2010 Jun;47(6):539 |
| 113 | 191 | GSE17329 | 9921,1 |  |  |
|  |  |  |  |  | 50. |
| 114 | 192 | GSE17329 | 70,6 |  |  |
|  |  |  |  |  |  |
| 115 | 193 | GSE17329 | 10266,5 |  |  |
| 116 | 194 | GSE17329 | 5895,2 |  |  |
| 117 | 195 | GSE17329 | 354,3 | 20,0 |  |
|  | 196 | GSE17329 |  |  |  |
| 118 | 197 | GSE17329 | 5512,7 | 336,7 |  |
|  | 198 | GSE17329 |  |  |  |
| 119 | 199 | GSE17329 | 79,8 |  |  |
| 120 | 200 | GSE17329 | 44,0 |  |  |
| 121 | 201 |  | 3051,0 | 409,7 |  |
|  | 202 |  |  |  | Kwon MJ, et al. The transcriptomic fingerprint of glucoamylase over- |
|  | 203 |  |  |  |  |
| 122 | 204 |  | 761,3 | 28,5 | expression in Aspergillus niger. BMC Genomics. 2012 Dec 13;13:701. |
|  | 205 |  |  |  |  |
|  | 206 |  |  |  |  |
| 123 | 207 |  | 1520,6 | 364,1 |  |
|  | 208 |  |  |  | Ram et al., unpublished data; SCLA mutant analysis |
| 124 | 209 |  | 741,6 | 116,3 |  |
|  |  |  |  |  |  |
|  | 210 |  |  |  |  |
| 125 | 211 | GSE50523 | 27,5 | 1,4 | Schachtschabel D, et al. The transcriptional repressor TupA in Aspergillus |
|  | 212 | GSE50523 |  |  | niger is involved in controlling gene expression related to cell wall |
| 126 | 213 | GSE50523 | 35,5 | 9,2 | biosynthesis, development, and nitrogen source availability. PLoS One |
|  | 214 | GSE50523 |  |  | 2013;8(10):e78102. |
| 127 | 215 | GSE25497 | 95,4 | 31,7 |  |
|  | 216 | GSE25497 |  |  | de Bekker C, et al. Single tip transcriptomics of neighboring hyphae of |
|  | 217 | GSE25497 |  |  |  |
|  |  |  |  |  | Aspergillus niger. Genome Biol 2011 Aug 4;12(8):R71. |
|  | 218 | GSE25497 |  |  |  |
|  |  |  |  |  |  |
|  | 219 | GSE25497 |  |  |  |
| 128 | 220 | GSE32123 | 1944,1 | 37,4 |  |
|  | 221 | GSE32123 |  |  | Bleichrodt R, et al. Cytosolic streaming in vegetative mycelium and aerial |
|  | 222 | GSE32123 |  |  |  |
| 129 | 223 | GSE32123 | 7023,9 | 1206,9 | structures of Aspergillus niger. Stud Mycol 2013 Mar 15;74(1):31-46. |

224 GSE32123
225 GSE32123

130	226	GSE36439	32,3	1,1

227 GSE36439
228 GSE36439

131	229	GSE36439	30,4	2,4

230 GSE36439
231 GSE36439

_132_	_232_	_GSE36439_	_31,5_	_4,4_	van Leeuwen MR, et al. Germination of conidia of Aspergillus niger is

|  | 233 | GSE36439 |  | accompanied by major changes in RNA profiles. Stud Mycol 2013 Mar |
| --- | --- | --- | --- | --- |
|  | 234 | GSE36439 |  | 15;74(1):59-70 |
| 133 | 235 | GSE36439 | 29,3 | 0,7 |

236 GSE36439
237 GSE36439

134	238	GSE36439	28,0	3,9

239 GSE36439
240 GSE36439

135	241	GSE36440	32,6	1,9

242 GSE36440
243 GSE36440

| 136 | 244 | GSE36440 | 27,3 | 1,8 |  |
| --- | --- | --- | --- | --- | --- |
|  | 245 | GSE36440 |  |  | van Leeuwen MR, et al. The effect of natamycin on the transcriptome of |
|  | 246 | GSE36440 |  |  |  |
| 137 | 247 | GSE36440 | 34,8 | 1,7 | conidia of Aspergillus niger. Stud Mycol 2013 Mar 15;74(1):71-85 |

248 GSE36440
249 GSE36440

| 138 | 250 | GSE36440 | 26,7 | 0,7 |  |
| --- | --- | --- | --- | --- | --- |
|  | 251 | GSE36440 |  |  |  |
|  | 252 | GSE36440 |  |  |  |
| 139 | 253 | GSE40219 | 722,9 | 585,8 | Gruben BS, et al. GalX regulates the D-galactose oxido-reductive pathway |
|  | 254 | GSE40219 |  |  |  |
| 140 | 255 | GSE40219 | 259,3 | 208,8 | in Aspergillus niger. FEBS Lett 2012 Nov 16;586(22):3980-5. |
|  | 256 | GSE40219 |  |  |  |
| 141 | 257 | GSE42480 | 34,0 |  | Novodvorska M, et al. Trancriptional landscape of Aspergillus niger at |
| 142 | 258 | GSE42480 | 35,5 |  |  |
| 143 | 259 | GSE42480 | 26,8 |  | breaking of conidial dormancy revealed by RNA-sequencing. BMC Genomics |
| 144 | 260 | GSE42480 | 23,8 |  | 2013 Apr 11;14:246. |
| 145 | 261 | GSE42480 | 26,9 |  |  |
| 146 | 262 | GSE44391 | 13659,2 | 8655,3 |  |
|  | 263 | GSE44391 |  |  |  |
| 147 | 264 | GSE44391 | 9540,5 | 8403,3 |  |
|  | 265 | GSE44391 |  |  |  |
| 148 | 266 | GSE44391 | 3488,1 | 3407,0 | Krijgsheld P, Wosten HAB. Transcriptome Analysis of Zones of Colonies of |
|  | 267 | GSE44391 |  |  |  |
|  |  |  |  |  | the flbA strain of Aspergillus niger. Fungal Genom Biol 3:109. doi: |
| 149 | 268 | GSE44391 | 14092,3 | 582,1 |  |
|  |  |  |  |  | 10.4172/2165-8056.1000109 |
|  | 269 | GSE44391 |  |  |  |
|  |  |  |  |  |  |
| 150 | 270 | GSE44391 | 15759,2 | 1735,0 |  |
|  | 271 | GSE44391 |  |  |  |
| 151 | 272 | GSE44391 | 7312,6 | 920,7 |  |
|  | 273 | GSE44391 |  |  |  |
| 152 | 274 | GSE51023 | 1447,1 | 353,7 | Gruben BS, et al. Aspergillus niger RhaR, a regulator involved in L- |
|  | 275 | GSE51023 |  |  |  |
|  |  |  |  |  | rhamnose release and catabolism. Appl Microbiol Biotechnol 2014 |
| 153 | 276 | GSE51023 | 536,9 | 58,0 |  |
|  |  |  |  |  | Jun;98(12):5531-40. |
|  | 277 | GSE51023 |  |  |  |
|  |  |  |  |  |  |
| 154 | 278 | GSE46187 | 22,0 | 1,2 |  |
|  | 279 | GSE46187 |  |  | Benoit I, et al. Bacillus subtilis attachment to Aspergillus niger hyphae |
|  | 280 | GSE46187 |  |  |  |
|  |  |  |  |  | results in mutually altered metabolism. Environ Microbiol 2015 |
| 155 | 281 | GSE46187 | 22,1 | 4,6 |  |
|  |  |  |  |  | Jun;17(6):2099-113. |
|  | 282 | GSE46187 |  |  |  |
|  |  |  |  |  |  |
|  | 283 | GSE46187 |  |  |  |

*If made available by the authors
